# Influence of Transition-Metal Ions on the Photoisomerization of a Pentadentate Azopyridine Ligand: Interplay of Charge-Transfer and Metal-Centered States

**DOI:** 10.1021/acs.inorgchem.6c02962

**Published:** 2026-08-19

**Authors:** Sebastian Megow, Lydia Adam, Niels Michaelis, Annika Prax, Henrike-Leonie Fitschen, Christian Näther, Friedrich Temps, Felix Tuczek

**Affiliations:** † Institut Für Physik, Freie Universität Berlin, Arnimallee 14, 14195 Berlin, Germany; ‡ Institut Für Anorganische Chemie, Christian-Albrechts-Universität zu Kiel, Max-Eyth-Straße 2, 24118 Kiel, Germany; § Institut Für Physikalische Chemie, Christian-Albrechts-Universität zu Kiel, Max-Eyth-Straße 1, 24118 Kiel, Germany

## Abstract

The influence of Zn^II^, Fe^II^, and Co^III^ metal centers on the photoisomerization of a pentadentate azopyridine ligand, a potential scaffold for ligand-driven light-induced spin change (LD-LISC) systems, is investigated. Steady-state UV–vis and NMR spectroscopy reveal a systematic increase in the *cis*-isomer fraction in the photostationary state from the Fe^II^ to the Co^III^ to the Zn^II^ complex. Time-resolved electronic absorption spectroscopy links these trends to distinct competing ultrafast relaxation pathways. While the Zn^II^ complex exhibits predominantly ligand-centered (LC) excited-state dynamics, the Fe^II^ analogue shows rapid population of low-lying metal-centered (MC) quintet states, as we reported previously. The isoelectronic Co^III^ complex exhibits an alternative relaxation pathway that partially restores the desired photoswitching behavior; however, weak signatures of a long-lived excited-state species remain detectable. Spectroelectrochemical measurements combined with quantum chemical calculations identify a ^3^MC state as the most likely origin of this residual signal. These findings further highlight the critical role of charge-transfer states in controlling photoswitching efficiency and underscore fundamental limitations in the design of LD-LISC systems.

## Graphical abstract

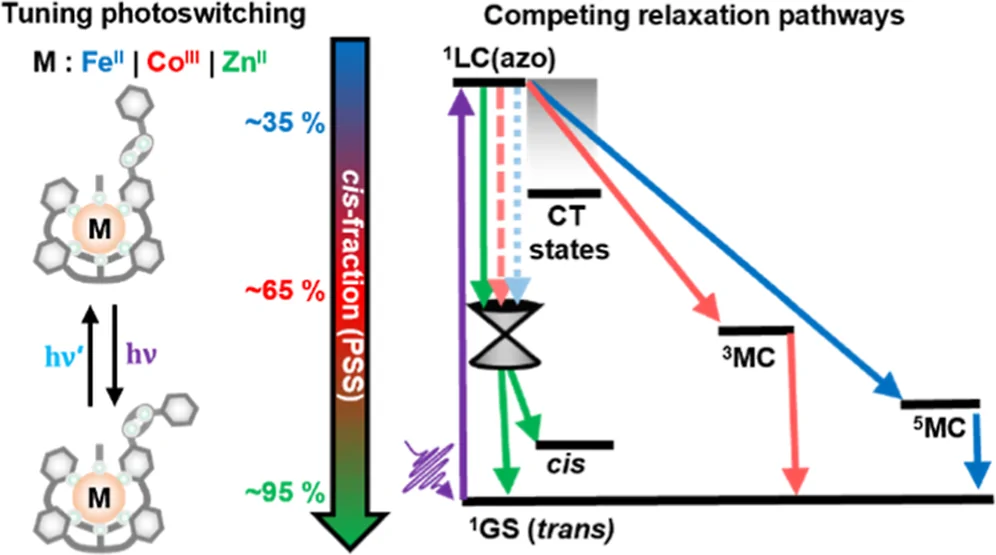

## Introduction

Transition-metal coordination compounds are promising candidates for the development of photoactive molecular materials, tailored to, e.g., energy conversion^1,2^ or photoredox catalysis.^3,4^ In both of these cases, suitable photosensitizers are characterized by photoexcited states with sufficiently long lifetimes to enable electron transfer/energy transfer reactions to outcompete other deactivation pathways.^5^ In view of their favorable redox properties, the development of photosensitizers based on first-row transition metals, such as Fe and Co, is an active area of research. While considerable advances have been made in recent years,^6–15^ achieving long-lived excited states and efficient photochemical function continues to represent a major challenge.^16^ Compared to their second or third row homologues, first-row transition metals exhibit significantly contracted *d*-orbitals originating from intrinsic wave function properties, often referred to as the “primogenic effect,” ^17,18^ which results in a high overlap of the radial distribution functions (RDFs) of the 3*s*, 3*p*, and 3*d* shells at a given distance *r*. The chemical consequence is a smaller ligand field splitting Δ_O_ for corresponding coordination compounds than encountered in 4*d* and 5*d* systems,^19,20^ inducing changes in relative excited-state energies for systems with different Δ_O_ values, especially those associated with metal-centered (MC) transitions. Since the energies of metal-to-ligand charge-transfer (MLCT) states are more or less invariant to Δ_O_ changes, the lowest lying excited state for 3*d*-metal-complexes is MC in nature, whereas MLCT states become the lowest excited states for 4*d*- and 5*d*-complexes.^17^ This change in order has a profound effect on photophysical properties: Whereas photoexcitation results in long living MLCT states for 4*d*- and 5*d*-metals capable to engage in bimolecular photochemistry, rapid nonradiative relaxation processes present in 3*d*-metals serve as efficient deactivation pathways, a direct result of the lower lying MC states.^21,22^ In particular, this rapid decay in excited MC states results in the observation of light-induced excited spin-state trapping (LIESST),^23,24^ as MC states with higher spin multiplicity compared to the ground state are formed during the relaxation.^25,26^ These excited states are undesired byproducts when it comes to photoredox chemistry but can become relevant for applications in fields that make use of the interesting magnetic properties of these materials; e.g., high-density optical data storage^27^ or medical imaging.^28^ However, increasing the lifetime of these excited high-spin (HS) states is crucial for further implementation.

Besides variation in combined σ- and π-donor strengths of supporting ligands, two concepts have been proposed to achieve magnetic bistability with improved excited-state lifetimes in third-row transition-metal complexes: light-driven coordination-induced spin state switching (LD-CISSS)^29,30^ and ligand-driven light-induced spin change (LD-LISC).^31,32^ Both concepts rely on the implementation of a photoswitchable chromophore, e.g., azobenzene or derivatives that either coordinate/decoordinate upon excitation (LD-CISSS) or indirectly induce a spin change due to different Δ_O_ values of the respective photoisomers (LD-LISC). Typically, Fe^II^-based systems have been targeted based on the drastic change of magnetic properties in its different spin states (^1^A_1_, S = 0 vs ^5^T_2_, S = 2). However, LD-LISC systems often suffer from poor conversion efficiencies and a lack of reversibility.^33,34^ In a previous study, we investigated the ultrafast photodynamics of the photoswitchable tetrapyridine-azopyridine (3Azo-N4Py) ligand **1** (Figure 1) and its Fe^II^ complex (**1-Fe** ^**II**^, cf. Figure 1) a potential candidate for future LD-LISC systems. We observed a rapid transfer of excitation energy from the photoexcited *trans*-azo-moiety into a metastable MC state. This relaxation pathway competes with the intended *trans*–*cis* isomerization of the azo unit, which was expected to promote the formation of a stable HS isomer of **1-Fe** ^**II**^.^35^

**Figure 1:**
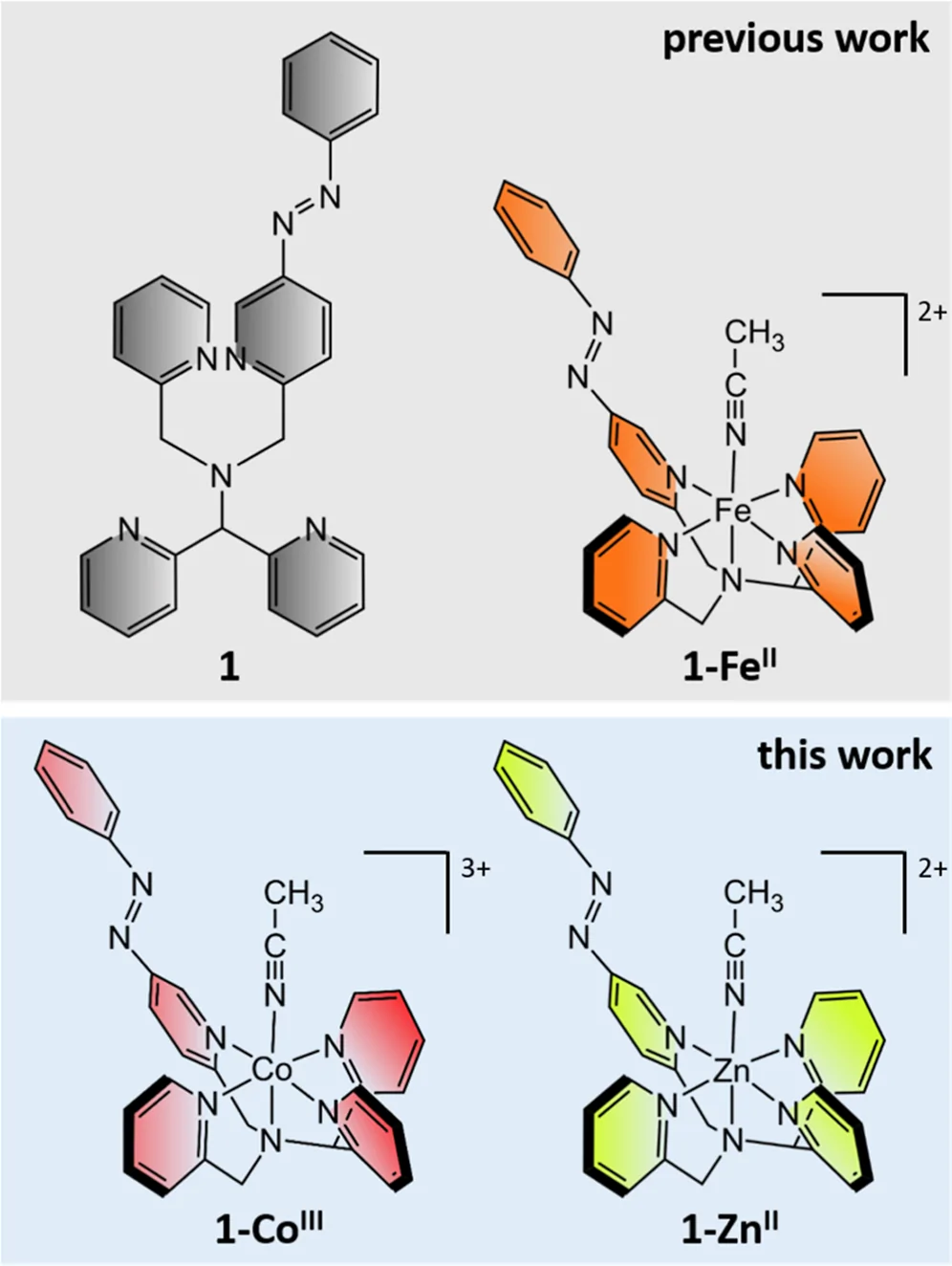
Schematic depiction of the 3Azo-N4Py ligand **1**, the previously studied complex **1-Fe** ^**II**^, and the new complexes **1-Co** ^**III**^ and **1-Zn** ^II^ investigated in this study.

These findings highlight a fundamental challenge of the LD-LISC concept. Efficient operation requires preservation of the intrinsic photochemical functionality of the ligand—in this case, azo isomerization—while simultaneously demanding sufficiently strong ligand–metal coupling to ensure that a structural change at the ligand can induce spin crossover (SCO) at the metal center. Excessive coupling, however, may redirect excitation energy into MC relaxation channels before productive ligand isomerization can occur.^36^ This intrinsic trade-off may explain why fully reversible and quantitative LD-LISC systems have so far remained elusive.

The dominant role of the described alternative relaxation pathway was attributed to strong coupling between ligand-centered (LC) and MLCT states. In Fe^II^ complexes, MLCT states typically lie at relatively low energies and efficiently funnel excitation energy into low-lying MC states. In addition, MLCT states exhibit pronounced absorption features in the visible region, with extinction coefficients on the order of ε ∼ 10^4^ L^–1^ M^–1^. These intense bands strongly overlap with ligand-centered absorptions, thereby limiting the spectroscopic accessibility of the ligand states. To further elucidate the interplay between ligand- and metal-centered excited states, we extended our investigation of **1** as a ligand scaffold for LD-LISC systems to the complexes **1-Co** ^**III**^ and **1-Zn** ^**II**^ (cf. Figure 1). The closed-shell d^10^-complex **1-Zn** ^**II**^ serves as an appropriate reference system for probing the intrinsic photochemical properties of the ligand within its coordination geometry, largely decoupled from metal-centered excited states. In contrast, the Co^III^ complex **1-Co** ^**III**^ allows us to examine the role of CT states in mediating ligand–metal coupling. This isoelectronic substitution is expected to leave the energies of the MC states largely unchanged while the altered redox properties of the metal center transform the character of the CT bands from MLCT in Fe^II^ to ligand-to-metal charge-transfer (LMCT) in Co^III^ complexes. Consequently, the CT absorption bands should significantly blue-shift into the ultraviolet region. This spectral separation is anticipated to reduce overlap with LC transitions and to modify the coupling between LC, CT, and MC states, thereby providing a more differentiated view of the competing relaxation pathways relevant to the LD-LISC concept. To this end, we investigated all compounds using a combination of quantum chemical calculations, steady-state UV–vis and NMR spectroscopy, femtosecond time-resolved electronic absorption spectroscopy, and UV–vis spectroelectrochemistry (UV–vis SEC). The transient absorption data for **1** and **1-Fe** ^**II**^ are reproduced from our previous work to facilitate direct comparison with the newly studied complexes **1-Co** ^**III**^ and **1-Zn** ^**II**^.

## Results and Discussion

### Synthesis and Characterization of 1-Co^III^ and 1-Zn^II^

The ligand 3Azo-N4Py (**1**) and the corresponding Fe^II^ complex **1-Fe** ^**II**^ are literature known and are synthesized accordingly.^35,37^ For **1-Zn** ^**II**^, this procedure could be adopted as well. However, instead of precipitating product **1-Zn** ^**II**^ in a concentrated cold solution, solubility differences were exploited for purification as ligand **1** was poorly soluble in acetonitrile and very soluble in dichloromethane and **1-Zn** ^**II**^ *vice versa*. **1-Co** ^**III**^ was synthesized following the procedure of Yamaguchi et al. for the preparation of a similar Co^III^ complex, producing the Co^III^ complex first and then oxidizing it with Ag^I^ salt.^38^ Unfortunately, **1-Co** ^**II**^ is highly unstable and cannot be isolated, even when the synthesis is carried out under an argon atmosphere. Therefore, *in situ* oxidation with AgClO_4_ was pursued first. Following this procedure, we were able to obtain **1-Co** ^**III**^ as a perchlorate salt, but the product contained significant amounts of unreacted silver perchlorate and decomposition products of **1-Co** ^**II**^. We then used silver triflate (AgOTf) as an oxidant. However, both AgClO_4_ and AgOTf are almost insoluble in dichloromethane, which results in long reaction times. This was incompatible with our system because of the quick decomposition of the **1-Co** ^**II**^ intermediate. Using acetonitrile, capable of dissolving all reactants, was not possible either due to the poor oxidation strength of Ag^I^ salts in acetonitrile.^39^ At last, we found that the triflimide (N­(CF_3_SO_2_)_2_^–^) salts of Co^II^ and Ag^I^ are soluble in diethyl ether. Intermediate complex **1-Co** ^**II**^ is soluble in dichloromethane, and **1-Co** ^**III**^ is insoluble in both solvents. Using the increased solubility of Ag^I^ triflimide compared to other Ag^I^ salts, we thus were able to first synthesize **1-Co** ^**II**^ in a mixture of dichloromethane and acetonitrile. After removal of most of the solvent, the oxidation step was carried out in a mixture of diethyl ether with a minimum amount of acetonitrile to dissolve **1-Co** ^**II**^. Product **1-Co** ^**III**^ was obtained as an orange solid after filtration and extensive washing with diethyl ether and dichloromethane to remove all of the remaining reactants. **1-Co** ^**III**^ is air stable but may not be dried under reduced pressure, leading to decomposition.

Crystals suitable for single-crystal analysis were obtained via vapor diffusion of diethyl ether into a solution of **1-Co** ^**III**^ in a 1:10 mixture of acetonitrile/dichloromethane. The **1-Co** ^**III**^ complex cation is the first structural characterization of an 3AzoN4Py system. It exhibits a distorted octahedral coordination of four pyridines, one tertiary amine-N and an acetonitrile ligand around the Co^III^ center (cf. Figure 2). The 3AzoN4Py ligand is a derivative of the N4Py ligand that contains the same amine and pyridine donors, but lacks the azo unit. Its Fe^II^ ^40^ and Co^III^ ^41^ complexes can be used for a structural comparison with **1-Co** ^**III**^. Across all six donor atoms, the M-N bonds are longer in the Fe^II^(N4Py) complex than in both Co^III^ complexes. The two Co^III^ complexes differ only in the bond length of the nitrogen of the pyridine carrying the azo unit in **1-Co** ^**III**^. Its bond length is 0.016 Å larger than in the corresponding Co^III^(N4Py) complex, while the other M–N bond lengths show no significant difference (cf. Table S1). The crystal structure of the **1-Co** ^**III**^ complex cation shows only the *trans*-isomer of the azo unit.

**Figure 2:**
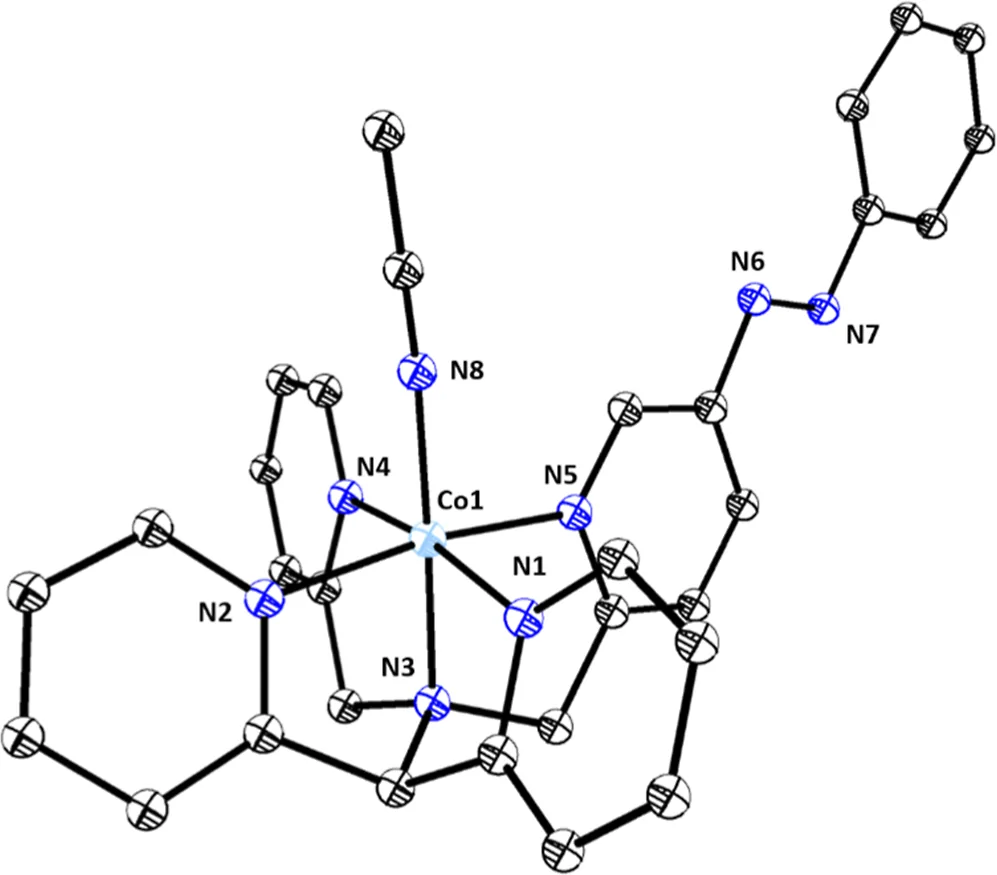
Molecular structure of **1-Co** ^**III**^ determined by single-crystal X-ray diffractometry. Displacement ellipsoids are drawn at the 50% probability level. Hydrogen atoms have been omitted for clarity.

### Electronic Structure and UV/Vis Absorption Spectra

The stationary UV–vis absorption spectra of **1**, **1-Zn** ^**II**^, **1-Fe** ^**II**^, and **1-Co** ^**III**^ measured in acetonitrile are displayed in Figure 3 alongside the TDDFT-calculated singlet transitions. For all compounds, the spectra are scaled by an arbitrary factor of 0.9 to better reproduce the experimental spectra. Additionally, results of the transition density matrix (TDM) analysis are given in the panel below the spectrum. This fragment-based analysis allows us to characterize each of the transitions as ligand-centered (LC), metal-centered (MC), ligand-to-ligand-charge transfer (LLCT), ligand-to-metal charge transfer (LMCT), or metal-to-ligand charge transfer (MLCT). The initial spectrum of **1** (Figure 3A) exhibits an intense absorption band with a maximum at λ = 325 nm and a less intense feature at 450 nm that can be assigned to the ππ* state and the nπ* state of the *trans*-isomer, respectively. These transitions are also predicted by the calculations. However, an additional transition appears in the calculations at around 470 nm, which originates from an intraligand CT state in which electron density is shifted from the pyridyl moieties to the azopyridine substituent of **1**. The TDM analysis of **1**, of course, only comprises one fragment and all transitions are consequently of LC character.

**Figure 3:**
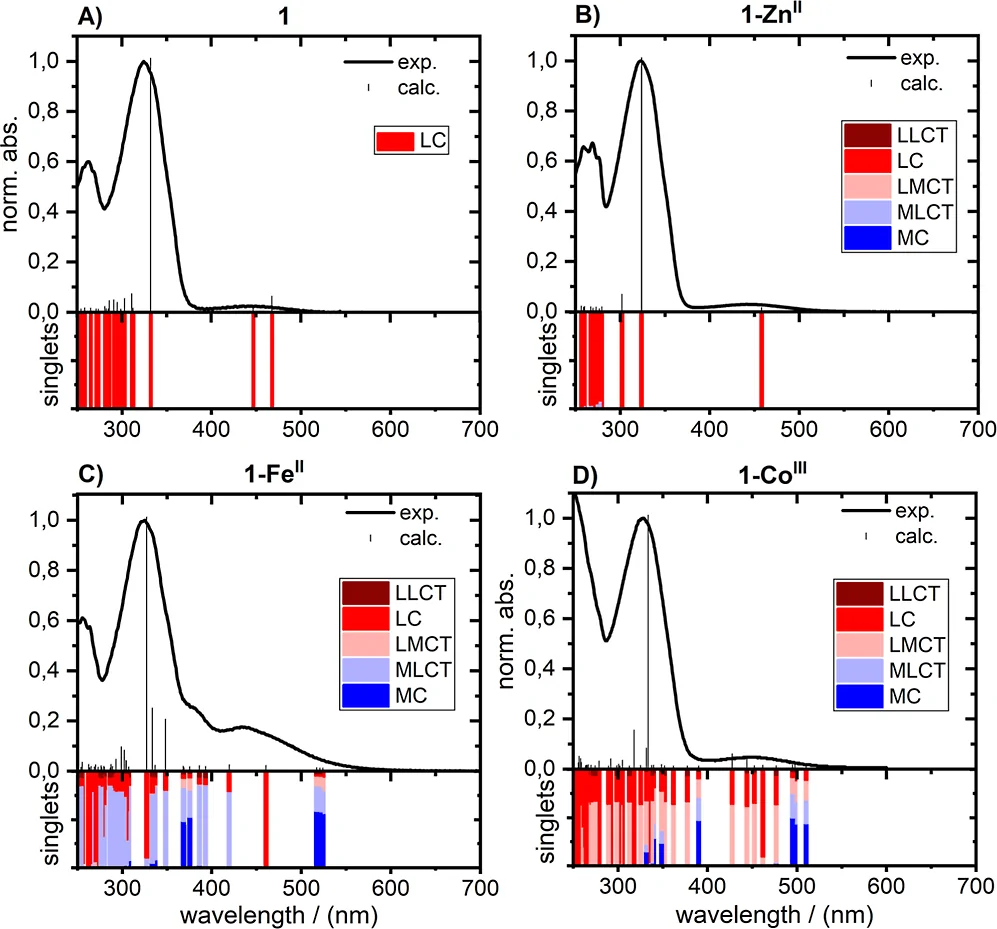
Experimental UV—vis absorption spectra and calculated electronic transitions of (A) **1**, (B) **1-Zn** ^**II**^, (C) **1-Fe** ^**II**^, and (D) **1-Co** ^**III**^ with an assigned character of the transitions based on a transition density matrix (TDM) analysis.

Complex **1-Zn** ^**II**^ (Figure 3B) exhibits a virtually identical absorption spectrum compared with **1**. The TDM analysis shows that all transitions in the relevant spectral range are LC in nature, which can be anticipated from the closed-shell nature of the Zn^2+^ ion (d^10^-configuration). Still, this complex represents a valuable reference, as it allows us to study the influence of the coordination geometry on ligand **1**, which, due to its decreased structural flexibility, is devoid of the CT transition at 470 nm that showed up in the calculations for **1**.

For **1-Fe** ^**II**^ (Figure 3C), a pronounced increase in absorption intensity is observed in the visible region. TDDFT predicts several transitions in this range, with the most intense bands assigned to MLCT excitations from Fe 3d to ligand π* orbitals. The calculated MLCT bands are shifted to slightly higher energies relative to the experiment, consistent with the known limitations of standard hybrid functionals for charge-transfer states.^42^ Despite this quantitative deviation, the TDDFT results combined with TDM analysis provide a reliable qualitative description of the excited-state manifold in **1-Fe** ^**II**^, clearly confirming the MLCT character of the dominant visible transitions. The LC nπ* state, predicted around 450 nm, is largely masked by the intense MLCT absorption. In addition, metal-centered (MC) excitations between the occupied t_2g_ and unoccupied e_g_ orbitals of the Fe^2+^ center give rise to the lowest singlet excited state (^1^T_1_). In the UV region (λ < 350 nm), the spectrum is dominated by a strong absorption band assigned to a LC ππ* transition, which is well reproduced by the calculations.

The spectrum of the isoelectronic complex **1-Co** ^**III**^ (Figure 3D) shows higher similarity to those of **1** and **1-Zn** ^**II**^, with dominant LC ππ* and nπ* transitions at approximately 325 and 450 nm, respectively. However, the UV region shows a noticeably increased absorption, which can be attributed to the presence of LMCT states in the high-energy range. The MC d–d transitions are again predicted as the lowest singlet excited states, appearing at energies comparable to those in **1-Fe** ^**II**^. This intended selective energetic modulation makes **1-Co** ^**III**^ a suitable model system for probing the influence of the CT energies on the energy transfer between ligand- and metal-centered excited states.

### Stationary Photoswitching Experiments

The stationary UV–vis absorption spectra of **1**, **1-Zn** ^**II**^, **1-Fe** ^**II**^, and **1-Co** ^**III**^ in their respective initial and photoswitched states, measured in acetonitrile, are displayed in Figure 4. The blue spectra were taken directly after preparing the solution, where the portion of the thermally favored *trans*-isomer is highest. The respective spectra in units of molar extinction are given in Figure S6 in the SI. The red spectra were taken after irradiation with a high-power LED at λ = 340 nm (43 mW) into a photostationary state (PSS). The gray spectra were taken in the PSS obtained after irradiation with a high-power LED at λ = 435 nm (440 mW). Each sample was irradiated for approximately 5 min. However, additional measurements indicated that the PSS was already reached within 1 min or less at the employed concentrations.

**Figure 4:**
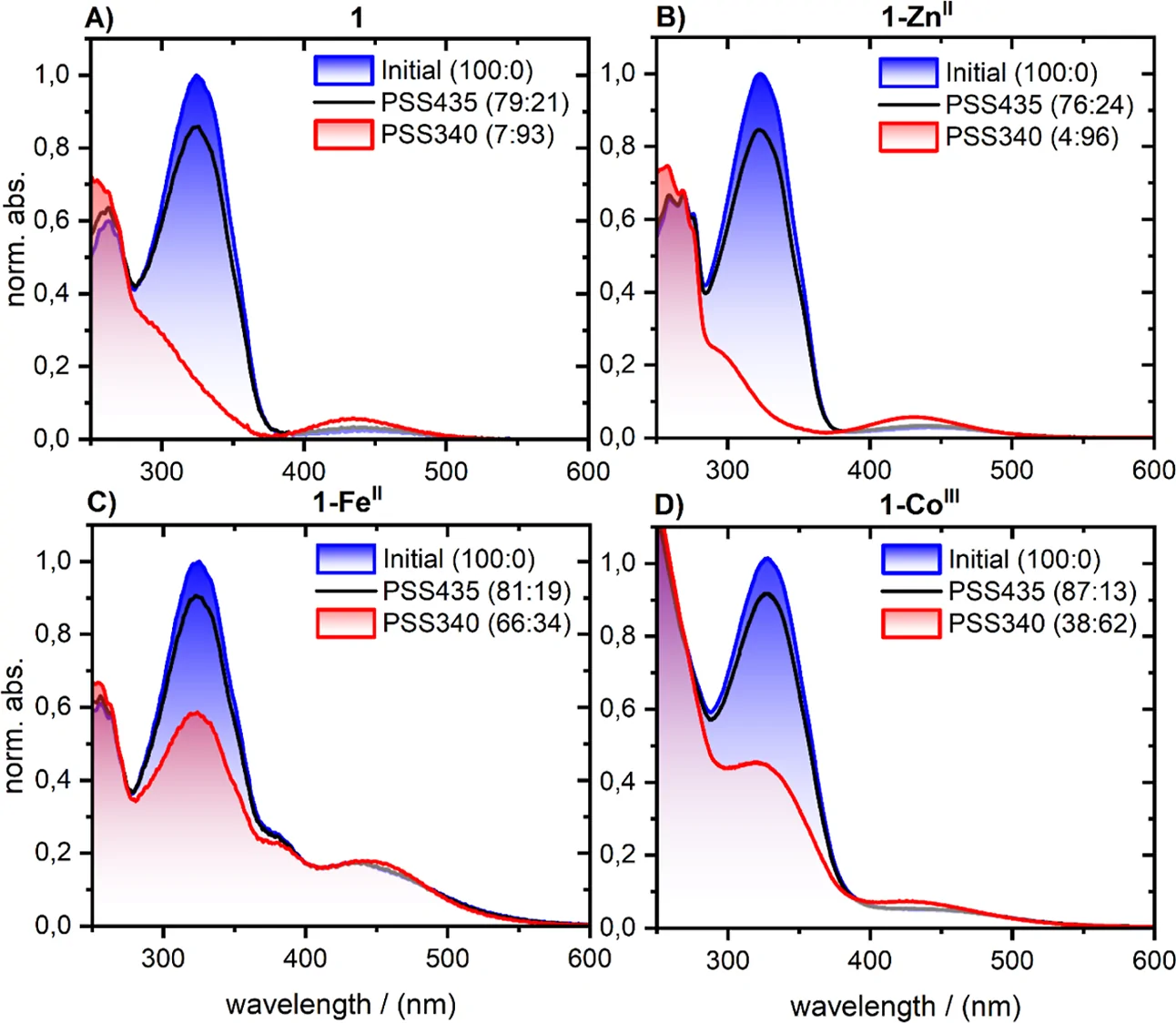
Stationary UV—vis absorption spectra recorded in acetonitrile of (A) **1**, (B) **1-Zn** ^**II**^, (C) **1-Fe** ^**II**^, and (D) **1-Co** ^**III**^ in the *trans*-configuration as well as the photostationary states (PSS) obtained upon irradiation with LEDs at 340 and 435 nm. The *trans*:*cis* ratios determined from NMR spectroscopy experiments are given in parentheses.

^1^H NMR spectroscopy was performed to determine the ratio between the *trans*-and the *cis*-isomers in the respective PSS (cf. Table S3, Figures S1–S4). This procedure is in more detail described in the SI. The thus determined *trans*:*cis* ratios are given in parentheses in Figure 4.

The initial spectrum of **1** (Figure 4A) exhibits an intense absorption band with a maximum at λ = 325 nm and a less intense feature at λ = 450 nm that can be assigned to the ππ* state and the nπ* state of the *trans*-isomer (see above). Since the planarity of the aromatic rings in *cis*-form of the azo-moiety is lifted, the intensity of the ππ* state decreases markedly. Consequently, upon continuous irradiation at 340 nm, the conversion to the *cis*-form is almost quantitative with only 7% of *trans*-isomer remaining in the PSS. On the other hand, continuous irradiation of the nπ* band, which has a slightly higher intensity in the *cis*-isomer allows one to obtain photostationary equilibria in favor of the *trans*-isomer. In fact, the PSS435 of **1** consists of 79% *trans*-isomer and only 21% *cis*-isomer.

Complex **1-Zn** ^**II**^ (Figure 4B) shows virtually identical spectra compared to those of **1**, since the closed-shell Zn^2+^ ion does not participate directly in the photochemistry of the complex and the relevant excited states retain their LC character. As a result, the stationary photoswitching is also very similar to that of **1**, with a PSS340 containing 96% *cis*-isomer and a PSS435 containing 76% *trans*-isomer.

For **1-Fe** ^**II**^ (Figure 4C), in addition to the apparently different absorption spectra, also a somewhat different photoswitching behavior is observed. Continuous irradiation of **1-Fe** ^**II**^ at 340 nm causes a much less pronounced decrease of the LC ππ* band around 350 nm indicating significantly less *trans*-to-*cis-*isomerization of coordinated ligand **1**. In fact, the determined *trans*:*cis* ratio shows that the PSS340 still contains an excess of 66% of *trans*-isomer. Interestingly, the portion of *trans*-isomer in the PSS435 of 81% is similar to the other compounds, suggesting that the photoisomerization process at smaller excitation energies is not affected.

The original photoswitching behavior is partially restored in isoelectronic complex **1**-**Co** ^**III**^ (Figure 4D). The spectra of **1-Co** ^**III**^ resemble those of **1-Zn** ^**II**^, except for the influence of the LMCT states that cause an increased absorption and a loss of structure in the UV part of the spectrum. The PSS340 of **1-Co** ^**III**^ is enriched with the *cis*-isomer, however, with only 62%, it still possesses a much smaller fraction than **1** and **1-Zn** ^**II**^, indicating again an impact of the central ion on the isomerization at these wavelengths, whereas the PSS435 shows a slightly higher amount of the *trans*-isomer (87%) compared to the other molecules.

### Ultrafast Transient Absorption Spectroscopy

Isomerization processes in azo-compounds occur on ultrafast time scales and are accompanied by large changes in the electronic absorption spectra of the concerned molecules. Therefore, femtosecond time-resolved electronic absorption spectroscopy (fs-TEAS) was employed in order to link the different photoisomerization ratios of ligand **1** in the three transition-metal complexes **1-Zn** ^**II**^, **1-Fe** ^**II**^, and **1-Co** ^**III**^, observed in the stationary experiments, to the respective photodynamic processes of these molecules on the ultrafast time scale. To induce the *trans*-to-*cis* isomerization, an excitation wavelength of λ_pump_ = 315 nm was used to excite **1** and the three derived complexes into the LC ππ* state of the azopyridine chromophore. Additional control experiments were carried out for **1, 1-Fe** ^**II**^, and **1-Co** ^**III**^ following excitation of their visible absorption bands at λ_pump_ = 460 nm (cf. Figure S9). The recorded two-dimensional spectro-temporal absorption maps showing the change in optical density (ΔOD) as a function of probe wavelength (λ_probe_) and pump–probe time delay (Δ*t*) are displayed in Figure 5 alongside with the transient absorption spectra at selected delay times. The evolution-associated difference spectra (EADS) and time constants, obtained from a global analysis of the experimental fs-TEAS data using the python-based software package KiMoPack,^43^ are given in the bottom row. These represent the individual spectral species required to model the experimental data in a consecutive kinetic scheme with increasing global lifetimes (i.e., $0\overset{h\nu }{\to }A\overset{\tau_{1}}{\to }B\overset{\tau_{2}}{\to }C\overset{\tau_{3}}{\to }D\overset{\tau_{4}}{\to }0$ ).

**Figure 5:**
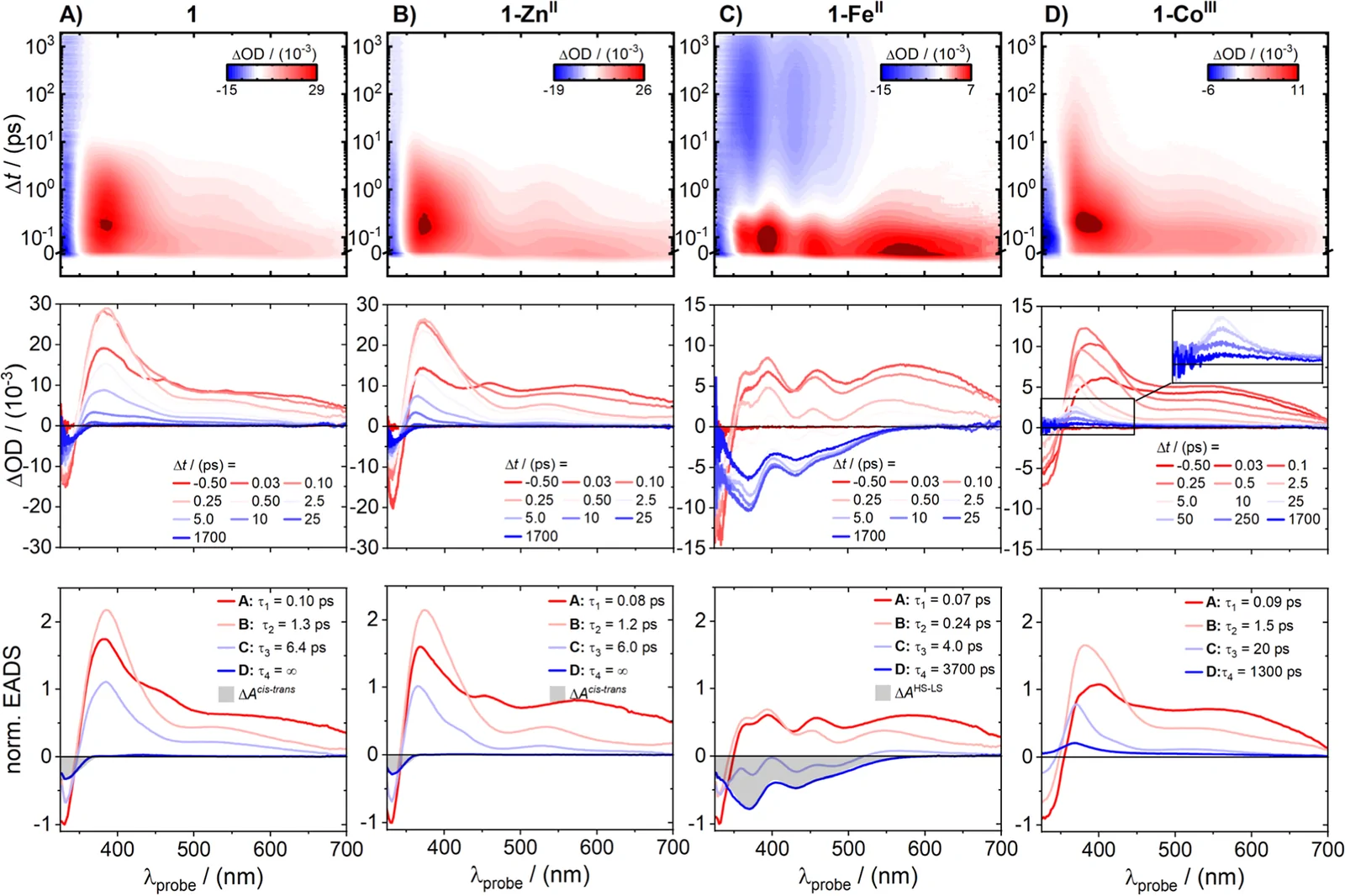
Two-dimensional spectro-temporal absorption maps, transient absorption spectra and evolution-associated difference spectra (EADS) with the corresponding time constants obtained from a global analysis of (A) **1**, (B) **1-Zn** ^**II**^, (C) **1-Fe** ^**II**^, and (D) **1-Co** ^**III**^. The data were recorded in acetonitrile following photoexcitation at λ_pump_ = 315 nm. Inset in the transient spectra in the middle row of column D displays the signature of the long-lived excited state observed for **1-Co** ^**III**^.

Selected time traces together with their corresponding global fits are given in Figure S7 and S9 of the SI. The experimental instrument response functions (IRFs), determined from the global fitting procedure, were ∼30 fs for excitation at 460 nm and ∼90 fs for excitation at 315 nm.

For **1** (Figure 5A), the initially observed broad excited-state absorption (ESA) with a slight peak at λ_probe_ = 450 nm is a characteristic for the photoproduced azopyridine ππ* state.^44–46^ The rapid conversion to the azopyridine nπ* state that features a prominent ESA band at λ_probe_ = 380 nm is completed within the first 250 fs and is not accompanied by a recovery of the ground-state bleach (GSB) at λ_probe_ = 330 nm. The nπ* state subsequently decays within 10 ps which is accompanied by a partial recovery of the GSB indicating a conversion to the ground state of the *trans*-isomer. The remaining permanent GSB feature indicates the formation of the *cis*-isomer. The global analysis yielded four components. The lifetime of the ππ* state, represented by the first EADS, was determined to τ_1_ = 100 fs, which is close to the experimental time resolution. This state internally converts to the nπ* state. Consequently, the two ensuing EADS with time constants τ_2_ = 1.3 ps and τ_3_ = 6.4 ps are assigned to evolution on the nπ* potential energy hypersurface (PEHS) and to the passing of the conical intersection (CI) to the ground state, respectively. The latter process is not only responsible for the generation of the *cis*-photoproduct but also recovery of the original (*trans*) ground state. The final EADS with infinite lifetime represents the fraction *cis*-photoproduct, which becomes clear from the matching of this spectrum with difference of stationary *cis*- and *trans*-spectra (ΔA^*cis–trans*^) displayed as the gray area. The quantum yield of this process can be estimated from the ratio of the initial to the final GSB signal, which is ∼25%. However, this value only represents an upper limit as the magnitude of initial bleaching signal remains uncertain due to the limited time resolution and overlapping ESA contributions. Overall, the photodynamics observed for **1** are in line with most studies carried out on azobenzenes and related compounds.^44,45,47^

Complex **1-Zn** ^**II**^ (Figure 5B) exhibits virtually identical photodynamics as **1**, with the initial conversion of the LC ππ* to the LC nπ* state and the subsequent recovery of the ground state, which is accompanied by the isomerization process leading to the formation of the *cis*-isomer. Again, an upper limit for the quantum yield of ∼25% can be estimated. Furthermore, the EADS and time constants are identical to those obtained for **1** and can consequently be linked to the same processes. This proves that coordination of ligand **1** does not exert a significant effect on its photodynamics.

The data recorded following photoexcitation of **1-Fe** ^**II**^ (Figure 5C) show major differences in its photodynamics. The initial spectrum features a broad positive absorption band with a peak at λ_probe_ = 450 nm that points at the photoproduced LC ππ* state. The ensuing spectra, however, rapidly evolve toward negative amplitudes. After 2.5 ps, the spectrum features negative absorption signals which are associated with the GSB of the MLCT absorption bands. The GSB of the LC ππ* state at λ_probe_ = 330 nm has, in contrast, recovered mostly, indicating an energy transfer between the two states, evidenced by the isosbestic point at λ_probe_ = 340 nm. The GSB of the MLCT bands further intensifies until it ultimately starts to recover after 10 ps. The complete recovery occurs on time scales beyond the 1.7 ns recorded in our measurements. The global analysis yields 4 species. We assign the first EADS to the initially excited LC ππ* state that decays with a time constant of τ_1_ = 0.07 ps, which is close to the time resolution of our experiment and thus contains a strong admixture of the successor state. The ensuing EADS is assigned to an MLCT state with a lifetime of τ_2_ = 0.24 ps. This assignment is based on a control experiment, in which **1-Fe** ^**II**^ was excited at λ_pump_ = 460 nm, i.e., directly into the MLCT state, which produces similar EADS with almost identical time constants (cf. Figure S8B). The MLCT state then converts to a MC quintet state (^5^MC) in which the Fe^2+^ ion adopts the high-spin (HS) electron configuration. This ^5^MC state undergoes vibrational cooling with a time constant of τ_3_ = 4 ps and relaxes back to the low-spin (LS) singlet ground state (^1^GS) with τ_4_ = 3.7 ns. The transient absorption spectrum of the ^5^MC state is well reproduced by the difference between the steady-state spectra of the HS and LS forms (ΔA^HS–LS^) of **1-Fe**. The spectrum of the HS form was obtained from measurements in methanol.^35^ This light-induced SCO is well reported as the major relaxation pathway for numerous Fe^II^ polypyridyl complexes following the excitation of the visible MLCT states.^48–53^ Reports on this behavior following excitation of LC states are, however, rare.^54,55^ In the case of **1-Fe** ^**II**^, initial excitation energy placed in the LC ππ* state of the azo unit is effectively funneled into the ^5^MC state of iron. This energy transfer process is itself ultrafast and competes with the intended photoisomerization of the azo unit. In the case of **1-Fe** ^**II**^, a time constant of 70 fs was extracted for initial LC → MLCT conversion from the global analysis, which must be viewed as an upper limit for this process. A more detailed discussion of the phenomenon can be found in our previous paper.^35^

For **1-Co** ^**III**^ (Figure 5D), the recorded transient absorption data show similarity to those obtained for **1** and **1-Zn** ^**II**^ before, indicating a predominant involvement of LC states of the azo group in the de-excitation process. However, careful inspection of the data reveals fundamental differences: In the case of **1-Co** ^**III**^, the initially observed transient spectrum exhibits a much broader spectral shape, lacking any distinct ESA peaks which can be observed for the other compounds; although according to calculation and the stationary UV–vis spectra at λ_pump_ = 315 nm, a LC ππ* state of the azo is predominantly excited. The ensuing transients, again, feature an intense ESA with a peak at λ_probe_ = 380 nm, indicating the presence of the LC nπ* state, but with a more pronounced broad absorption tailing into the visible region. Surprisingly, after ∼10 ps, the GSB signal has completely vanished while a weak ESA band with a peak at λ_probe_ = 370 nm and a broad tail extending in the visible region is still present, indicating the formation of an excited state species with exclusively positive absorption in the detected spectral range. Furthermore, this species possesses a long excited-state lifetime as it still can weakly be observed in the final transient recorded at 1700 ps. This becomes even clearer looking at the extracted EADS in Figure 5D: Owing to their spectral shape and similar lifetimes EADS A, B, and C are most likely associated with the photoproduced LC ππ* and subsequently populated LC nπ* state, respectively. However, the time constant τ_3_ = 13 ps is slightly longer compared to the τ_3_ found in **1** and **1-Zn** ^**II**^, potentially containing some admixed contributions. Ultimately, EADS D shows the weak but broad positive absorption of the final species with a long lifetime of τ_4_ = 1.3 ns. Very similar excited-state dynamics are observed upon excitation of **1-Co** ^**III**^ at λ_pump_ = 460 nm (cf. Figure S5C). At this wavelength, excitation predominantly populates the LC nπ* state of the azo unit. However, weak direct excitation of the metal-centered ^1^T_1_ ligand-field state cannot be excluded, as recently demonstrated by McCusker and co-workers.^56^ The origin of the long-lived species thus remains unclear. Based on the previous electronic structure calculations for **1-Co** ^**III**^ (see above), both LMCT and MC states must be considered as possible candidates. In addition, the spin–orbit coupling imposed by the Co^3+^ ion facilitating ISC processes also gives rise to different spin multiplicities of these states.

An earlier work by Nishihara et al. on tris­(bipyridine)­cobalt complexes bearing multiple azobenzene units reported a reduced fraction of *cis*-ligands in the photostationary state (PSS) upon coordination to a Co^III^ center, as observed for **1-Co** ^**III**^.^57^ This behavior was attributed to an electron-transfer process from the photoexcited azo moiety to the metal center, leading to reduction to Co^II^, i.e., population of a LMCT state. This assignment was supported by transient absorption spectra exhibiting a broad and long-lived absorption feature in the 500–700 nm range. However, their measurements did not extend below 400 nm, which is the spectral region where we observe the most pronounced signature of the present species. On the other hand, more recent investigations on a number of Co^III^ polypyridyl complexes have reported long-lived excited states, which were instead assigned to the Co^III 3^MC (^3^T_1_) state.^58–60^ Consequently, both LMCT and ^3^MC states remain viable candidates for the species observed here and require further discrimination. To this end, we performed spectroelectrochemical measurements complemented by additional electronic structure calculations.

### Electrochemistry

To gather information on possible transient electronic states formed during the relaxation process for photoexcited **1-Co** ^**III**^, cyclic voltammetry (CV) and UV–vis spectroelectrochemistry (UV–vis SEC) were conducted on **1** and **1-Co** ^**III**^. Notably, a combination of both methods has been used to successfully identify charge-transfer states for Fe and Ru systems.^61^ A cyclovoltammogram of **1** in abs. MeCN (50 mM TBA-NTf_2_, Pt WE, Pt CE) vs Fc/Fc^+^ is presented in Figure 6A. The survey scan of **1** revealed two redox systems at *E*_1/2_ = −1.68 V (ΔE_c‑p_ = 90 mV) and E_pa_ = 0.85 V, corresponding to the [N = N]^0/–2^ redox-couple and oxidation of the ligand, respectively (see SI cf. Figures S10–S13, Table S4 for a detailed discussion). The small oxidative features present in the −0.7 to −1.4 V range are due to a byproduct of the irreversible oxidation at 0.85 V.

**Figure 6:**
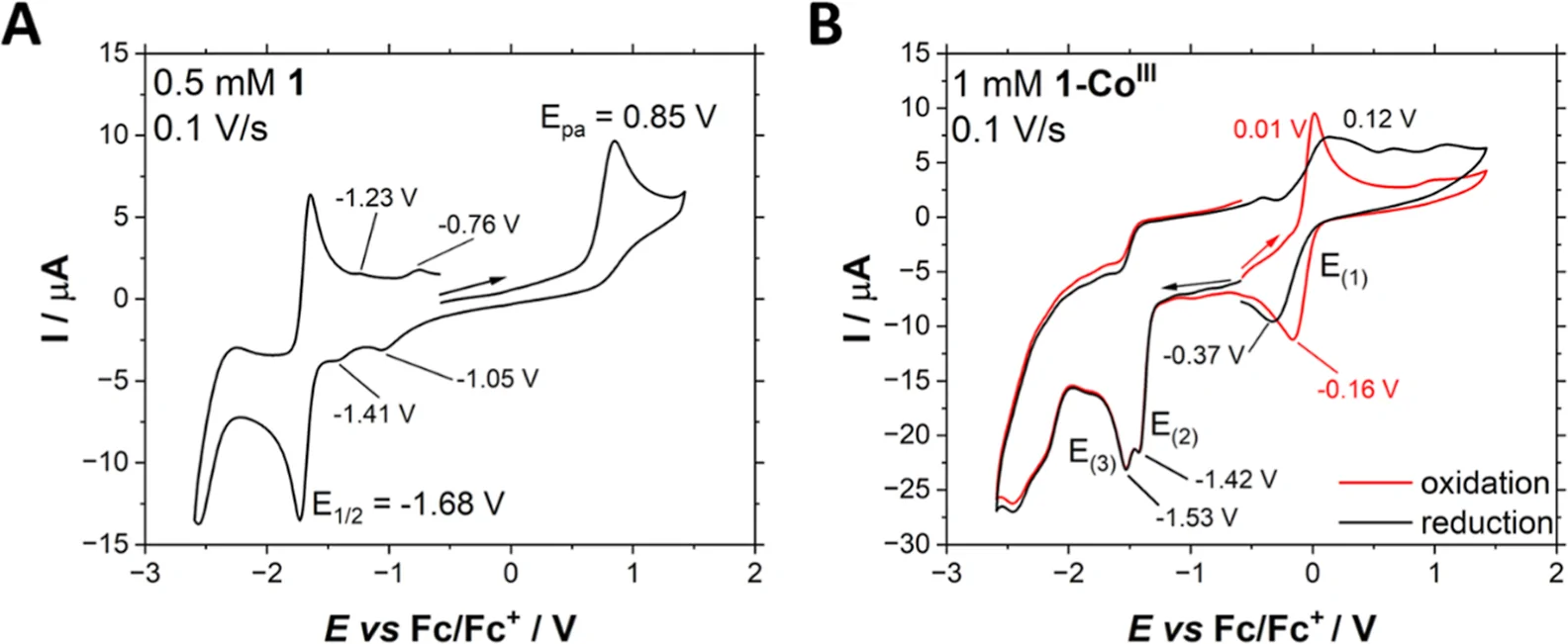
**A:** Survey scan of 0.5 mM **1** in 50 mM TBA-NTf_2_/MeCN at 0.1 V/s. **B:** Oxidative (red) and reductive (black) scans of 1 mM **1-Co** ^**III**^ in 50 mM TBA-NTf_2_/MeCN.

The electrochemical investigation of **1-Co** ^**III**^ was conducted in 50 mM TBA-NTf_2_ to prevent counterion exchange reactions with the electrolyte (Figure 6B). Starting just below the open-circuit potential (OCP), only one major anodic peak at *E*_a_ = 0.01 V appears inside the potential range of the cell. Upon reversion of the scan direction, the corresponding cathodic peak is detected at E_c_ = −0.16 V (cf. Figure 6B, E_(1)_). Further reduction leads to two closely spaced cathodic peaks at −1.42 V (E_(2)_) and −1.53 V (E_(3)_), with additional small reductive waves during the onset of MeCN reduction below −2.0 V. No additional anodic peaks are observed upon reversal of the scan direction, rendering processes E_(2)_ and E_(3)_ irreversible. This has a pronounced effect on E_(1)_, as the peak-to-peak separation increases with additional loss of peak current. These observations indicate possible structural changes or rearrangement during processes E_(2)_ and E_(3)_. This is especially visible when performing square-wave voltammetry (cf. Figure S15), where scanning from −1.75 to 1.0 V gives rise to two peaks at −0.068 and 0.168 V, contrary to the single reduction peak at −0.054 V when performing the experiment in the reversed direction. On the basis of a similar redox response of the azo-free Co^3+^-complex reported by Loet al., E_(1)_ can be assigned to the quasi-reversible Co^3+/2+^ redox couple.^62^ This indicates that the **1-Co** ^**II**^-redox state is stable on an electrochemical time scale but inaccessible during synthesis due to its high reactivity (cf. Figure S16, and SI for additional discussion).

Interpretation of E_(2)_ and E_(3)_ is less straightforward. Notably, controlled-potential electrolysis (CPE) of a 1.03 mM solution of orange **1-Co** ^**III**^ at −1.6 V turned deep blue and gave a transferred charge of −1.023 C after 3.5 h, corresponding to three total electrons involved in the reduction (cf. Figure S17). The first one-electron reduction corresponds to the Co^III^ to Co^II^ reduction (E_(1)_; see above), with the second and third electrons being consumed at E_(2)_ and E_(3)_, respectively. Elucidating the true nature of the fully three-electron-reduced species of **1-Co** ^**III**^ is beyond the scope of this work, but it likely involves a mixed {Co^I^[N = N]^−1^}^0^ complex where the second electron is consumed to reduce the metal center to Co^I^, while the third is transferred into the azo moiety (cf. Figures S16 and S18 for additional discussion).

### UV–vis Spectroelectrochemistry

To gather *in situ* information on the electronic structure of the electrochemically generated species, UV–vis SEC was conducted on 0.5 mM **1** and 1 mM **1-Co** ^**III**^ in 50 mM TBA-NTf_2_ in abs. MeCN under a N_2_-atmosphere. The two-electron reduction of the azo moiety of **1** initially leads to an increase for the ππ* absorption band at 325 nm (Figure 7, left). Maximum intensity is reached around −1.6 V, which is around *E*_1/2_ of the [N=N]^0/‑2^ redox couple (cf. Figure 6A). Further reduction triggers a decrease in intensity with a simultaneous rise in absorption for the band at 430 nm, corresponding to the [N=N]^−2^-redox state. The original spectrum is restored after reversion and subsequent reduction to the initial [N=N]^0^-redox state. This spectroscopic fingerprint is very similar to the PSS experiments of **1** (cf. Figure 3), although the band at 430 nm is much more intense under UV–vis SEC conditions. These spectroscopic similarities indicate reversible *trans-*to *cis-*isomerization of the azo moiety upon reduction, similar to azobenzene derivates.^63^ On the other hand, oxidation of **1** around 0.8 V (cf. Figure 5A) does not lead to any significant spectroscopic changes, indicating that this irreversible oxidation is indeed amine based (cf. Figure S19),^62^ unfortunately limiting the capability to identify specific spectral features of an oxidized azo-moiety.

**Figure 7:**
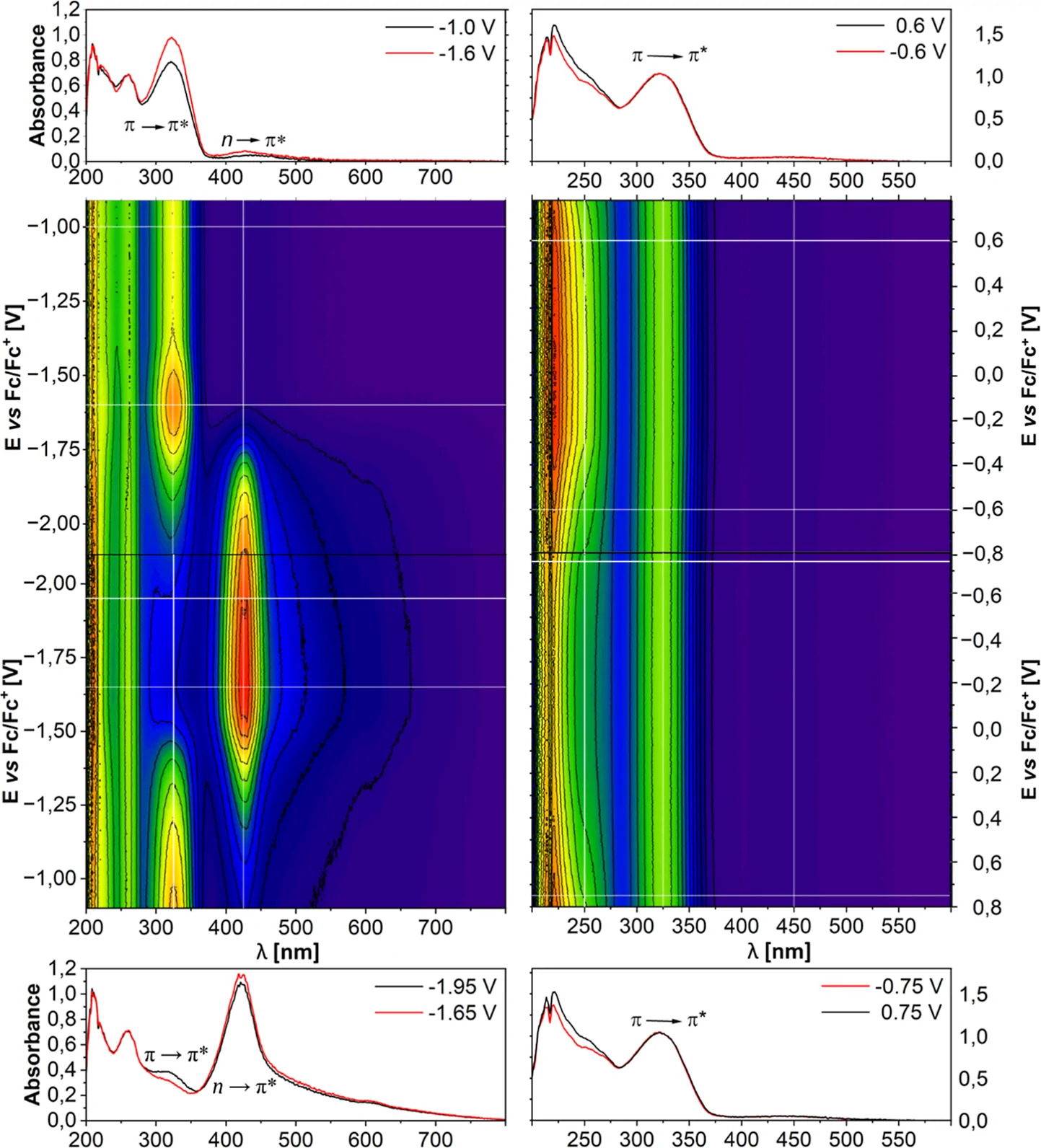
*Left:* UV–vis SEC of 0.5 mM **1** in 50 mM TBA-NTf_2_ in abs. MeCN at 0.02 V/s with a focus on the [N=N]^0/‑2^ redox-couple. *Right:* UV–vis SEC of 1.0 mM **1-Co** ^**III**^ in 50 mM TBA-NTf_2_ in abs. MeCN at 0.02 V/s with focus on the Co^3+/2+^ redox-couple (scan direction top to bottom). Top and bottom panel: Electronic absorption spectra at selected potentials.

UV–vis SEC of **1-Co** ^**III**^ in the potential range of the Co^3+/2+^ redox-couple reveals a small decrease of intensity in the 200–250 nm range upon reduction to the Co^2+^ redox state (cf. Figures 7 right, S20, S21). An additional UV–vis SEC experiment under CPE conditions at −1.0 V was conducted on **1-Co** ^**III**^ in the 300–600 nm range. In this measurement, only a very small decrease in intensity at around 425–450 nm could be detected (cf. Figure S22, S23). Importantly, these changes in absorbance are distinctively different from the spectral changes observed in the fs-TEAS data of **1**-**Co** ^**III**^ (cf. Figure 5D).

Performing UV–vis SEC over the full potential range of **1-Co** ^**III**^ (cf. Figure S21) leads to a decrease of the 325 nm band when reduced at −1.5 V; i.e., at E_(3)_, similar to the reduction of **1**. On the other hand, further reduction of **1-Co** ^**III**^ at potentials negative to E_(3)_ gives rise to a new absorption band between 500–550 nm. This not only differs from **1**, the electronic spectrum also is in stark contrast to our CPE results of **1-Co** ^**III**^ at this potential range (cf. Figure S14, right).

The original spectrum of **1-Co** ^**III**^ is restored upon reversing to 0.8 V, which is in good agreement with our previous results obtained by CV (cf. Figure 6B). While this further indicates a more complicated electronic structure for fully reduced **1-Co** ^**III**^, it also highlights the limitations of UV–vis SEC in this context: With the UV—vis spectra of an [N=N]^+^ intermediate unattainable due its high oxidation potential, and likewise, Co^2+^ transitions being masked by more prominent transitions, the observed UV—vis spectra can neither confirm nor disprove an assignment of the observed fs-TEAS data to a LMCT state of **1-Co** ^**III**^. However, the lack of luminescence (cf. Figure S24) from **1-Co** ^**III**^ would be highly uncommon for a populated LMCT state. Thus, the formation of a Co^2+^ species by population of an LMCT state during relaxation is rather unlikely and relaxation via a MC state remains as a plausible alternative.

### Simulation of the ^3^MC Excited-State Absorption Spectrum of 1-Co^III^

Photoexcitation of Co^III^ systems can lead to the formation of highly photoactive ^3^MC excited states with lifetimes in the nanosecond range.^60^ An early study by McCusker on hexadentate [Co­(tppn)]­(ClO_4_)_3_ (tppn = *N,N,Ń,Ń-*tetra­(pyridylmethyl)­1-methyldiaminoethane) reported a “very weak transient absorption” at 385 nm “with a long tail extending into the mid-visible region” following excitation of the LMCT states at 314 nm.^64^ More recently, UV—vis TA spectroscopy on [Co­(dqp)_2_]^3+^ (dqp = 2,6-di­(quinolin-8-yl)­pyridine) by Yaltseva et al.^58^ and co-workers revealed similar spectroscopic features. The authors proposed that, after photoexcitation of [Co­(dqp)_2_]^3+^, an ^1^ILCT (*intra ligand charge transfer*) state is populated that rapidly converts into the ^3^MC state. They also observe an ESA feature with a distinct peak at ∼400 nm and a broad tail extending into the visible region as well as an overall lifetime of the ^3^MC state in the nanosecond range. These observations are remarkably similar to our observations for **1-Co** ^**III**^. The authors’ interpretation was supported by TDDFT calculations simulating the transient absorption spectrum of the ^3^MC state. Following this approach, we modeled the TA spectrum of the lowest lying ^3^MC state of **1-Co** ^**III**^. The results are shown in Figure 8. The geometry and electronic absorption spectrum of the lowest lying triplet state (T_1_) of **1-Co** ^**III**^ were calculated at the same level of theory as the corresponding singlet ground state (S_0_), using an unrestricted Kohn–Sham formalism (UB3LYP) for the triplet state. The calculated transitions of the S_0_ (gray bars) and T_1_ (black bars) states were broadened using Gaussian functions with a full width at half-maximum (fwhm) of 0.2 eV. The simulated difference spectrum was generated by subtracting the S_0_ spectrum (GSB; dotted gray line) from the T_1_ spectrum (ESA; dotted black line). The resulting spectrum (blue curve) is in excellent agreement with the experimentally derived EADS of the long-lived (1.3 ns) excited-state species of **1-Co** ^**III**^. The calculated spin density of the T_1_ state further confirms its MC character. The simulation indicates that the characteristic transient feature of the ^3^MC state originates primarily from a subtle broadening and spectral shift of the LC UV absorption band, whereas the most intense transitions of the ground and excited states largely cancel each other. This explains both the absence of pronounced negative GSB features in the late-time transient absorption spectra and the comparatively weak overall signal intensity.

**Figure 8:**
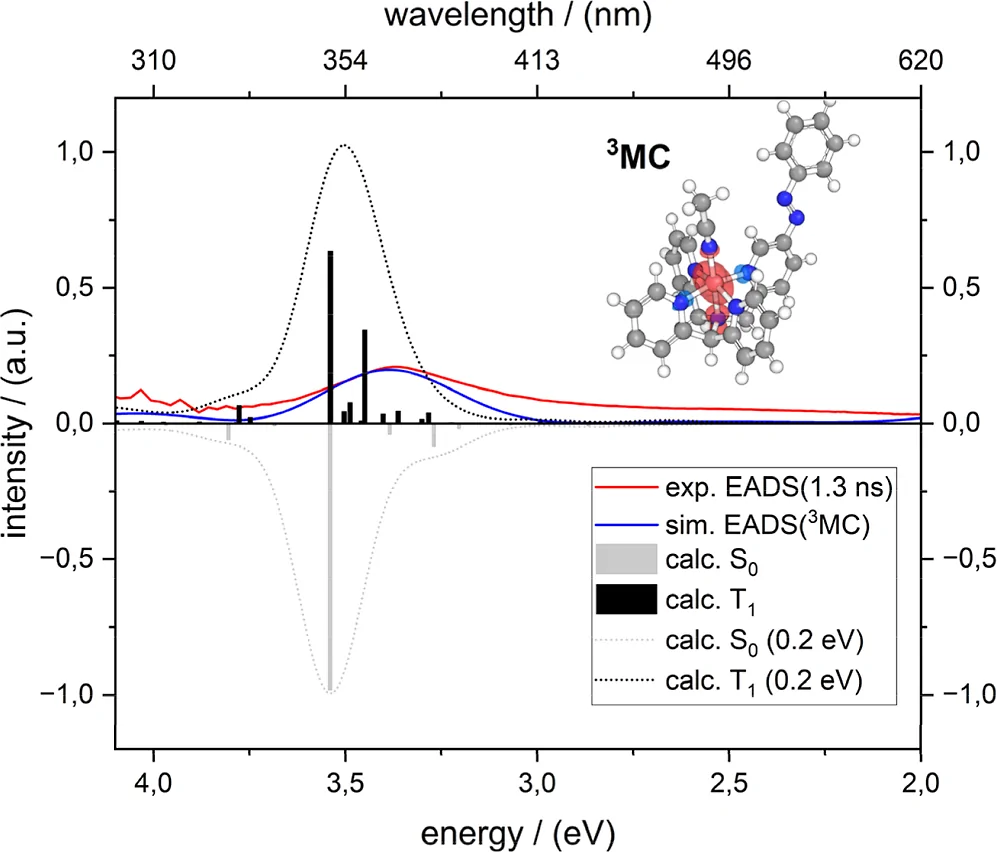
Experimental evolution-associated difference spectrum (EADS) of the 1.3 ns species observed for **1-Co** ^**III**^ (red curve) and simulated EADS for the ^3^MC state (blue curve). The simulated spectrum was obtained by the subtraction of the TDDFT calculated singlet ground state (S_0_; gray dotted) and triplet state (T_1_; black dotted) spectra. The calculated transitions (black and gray bars) were broadened with Gaussian functions (fwhm = 0.2 eV) and shifted by +0.2 eV to better match the experimental spectrum. The calculated spin density of the triplet state is also displayed, highlighting its metal-centered character.

Taken together with the SEC results, the lack of fluorescence, and the literature discussed above, these findings support assignment of the long-lived excited state of **1-Co** ^**III**^ to a ^3^MC state. Remarkably, this relaxation pathway would be analogous to that previously identified for **1-Fe** ^**II**^.^35^ Whether the population of the ^3^MC state proceeds directly from excited states of the azopyridine unit or involves intermediate charge-transfer (CT) states cannot be conclusively determined from the experimental data. In the case of **1-Co** ^**III**^, such CT states would possess a predominantly LMCT character, in contrast to the MLCT states identified in **1-Fe** ^**II**^. Nevertheless, the reduced density of low-lying CT states in **1-Co** ^**III**^ relative to that in **1-Fe** ^**II**^ likely diminishes competitive energy funneling into metal-centered states and is thus consistent with the enhanced *trans*–*cis* isomerization observed for **1-Co** ^**III**^.

## Conclusion

In the preceding sections, the *trans*-to-*cis* photoisomerization of the azo group in the 3Azo-N4Py ligand in its free form (**1**) as well as coordinated to Fe^II^, Zn^II^, and Co^III^ centers (cpds **1-Fe** ^**II**^, **1-Zn** ^**II**^, and **1-Co** ^**III**^) has been studied. Importantly, our investigations on **1-Co** ^**III**^ showed an increase of the *cis* fraction of the attached azopyridine unit after photoexcitation relative to that of the corresponding iron complex **1-Fe** ^**II**^; *i.e*., this fraction improved from 30% *cis*-isomer in **1-Fe** ^**II**^ to 60% *cis*-isomer in **1-Co** ^**III**^. This observation supports the hypothesis that low-lying CT states promote ultrafast energy transfer from LC azopyridine states to MC states, thereby suppressing *trans*-to-*cis* isomerization in the **1-Fe** ^**II**^ system. Despite this improvement, a substantial fraction of excitation energy is still transferred toward MC states during relaxation, as ultrafast transient absorption spectroscopy of **1-Co** ^**III**^ shows the formation of the ^3^MC ligand-field state with a lifetime of 1.3 ns. Whereas the coupling of LC and MC excited states is clearly influenced by fundamental properties of the metal center like *d-*electron count and ligand-field splitting, transition metals targeted for SCO are usually first-row *d* ^6^ or *d* ^7^ metal centers, where this coupling is strong. Triggering SCO by photoexcitation of azopyridine-related systems thus favors relaxation through MC states with very short lifetimes under ambient conditions, similar to the LIESST effect. This suggests a re-evaluation of the LD-LISC approach using azopyridine, as its underlying design concepts are opposed by fundamental excited-state dynamics. However, having shed light on these–at first glance–unfavorable relaxation processes, they also point in a direction where this field can evolve. While the relative energies of LC azo and MLCT states are primarily influenced by the choice of metal, MC state energies are drastically influenced by Δ_O_ and thus, overall ligand design. We propose here that future ligand designs should view the switching unit as a source for efficient energy capture instead of the driving force for SCO. Instead of tuning the ligand to support traditional SCO, Δ_O_ should be altered to increase the gap between ^1^MC ground state and excited^3,5^ MC states. A promising strategy for this would be to decrease the nonradiative decay by exploring SCO within the inverted Marcus region.^59^

## Experimental Section

### Sample Preparation and Characterization

Static UV–vis absorption spectra of all compounds were measured in spectroscopic grade acetonitrile (Uvasol) in a 1 mm quartz cuvette using a Shimadzu UV-2410 desktop spectrometer.

### Computational Methodology

Density functional theory (DFT)^65^ calculations were performed in order to gain a qualitative understanding on the electronic transitions of the investigated compounds. First, the singlet ground-state geometries were optimized using the Gaussian 09 revision D012 ^66^ software package with the B3LYP^67,68^ functional on a def2-TZVP basis set^69^ and polarizable continuum model (PCM)^70–72^ for acetonitrile. Second, the local minima of the optimized geometries were confirmed by frequency calculations on the previously optimized structures by obtaining no imaginary frequencies. Third, single point calculations on the first 20 for **1** and. 40 for **1-Zn** ^**II**^, **1-Fe** ^**II**^ and **1-Co** ^**III**^ vertical singlet transitions were performed on the optimized ground-state geometries using time-dependent DFT (TD-DFT),^73^ which yielded the vertical excitation energies and the corresponding oscillator strength. The resulting calculated transitions, ground-state absorption spectra are plotted alongside with the experimental spectra in Figure 3. For **1-Zn** ^**II**^, **1-Fe** ^**II**^, and **1-Co** ^**III**^, an additional transition density matrix (TDM) analysis was performed based on the single point calculations using the TheoDORE software package.^74^

### Femtosecond Time-Resolved Electronic Absorption Spectroscopy

The femtosecond transient electronic absorption spectroscopy (fs-TEAS) setup has in more detail been described elsewhere.^75^ In brief, the experiment consists of a Ti:Sa regenerative amplifier (Clark MXR CPA 2001) that delivers 120 fs fwhm (full width at half-maximum) pulses with 1 kHz repetition rate at a center wavelength of λ = 775 nm. Most of the 775 nm beam is used to run a home-built noncollinear optical parametric amplifier (NOPA) with a subsequent prism pulse compressor. Excitation pulses in the visible range, as used for the control experiments in the 450–460 nm range, were obtained directly from the compressed NOPA output. Excitation pulses in the UV at 315 nm were obtained from second harmonic generation (SHG) of the compressed NOPA output at 630 nm in a Type I BBO crystal. For broadband detection, a small fraction of the laser fundamental is sent through a CaF_2_ plate to generate supercontinuum probe pulses in the range of 325 nm < λ_probe_ < 700 nm, which are split into probe and reference beam, dispersed by a prism spectrograph and detected using fast frame-transfer CCD cameras. The pump and probe beams are focused to a spot size of ∼250 μm and ∼150 μm, respectively, and overlapped in a flow-cell with 1 mm optical path length and 0.2 mm thick CaF_2_ windows. The experimental instrument response function (IRF) parameter obtained from the global fit of the data is ∼30 fs if the compressed NOPA output is used directly, i.e., for excitation at 460 nm and ∼90 fs if the SHG of the NOPA output is used, i.e., for excitation at 315 nm. The samples were excited at an optical density (OD) of 0.4 with pulse energies of 250 nJ in the UV (OD 0.1 and 500 nJ in the visible). An optical chopper in the probe beam path, operated at half the repetition rate, blocks every second probe pulse, while another chopper in the pump beam path operated a quarter the repetition rate allows two consecutive pump pulses to pass while blocking the next two. This way the recorded transient absorption difference spectra are corrected for background contributions such as thermal drifts of the camera pixels and scattered pump light on a shot-to-shot basis.

### Electrochemistry and UV–vis SEC

Electrochemical studies were performed in a Inertec AG glovebox (ITA 14 Spez.) under N_2_–Atm. (O_2_ < 1 ppm) using a custom-made 3-electrode cell. (WE: Pt, RE: Ag-wire in a 1 mM Fc+/Fc, 0.1 M TBANTf_2_ in MeCN solution, CE: Pt-wire). Ferrocene was added at the end of the experiments to determine the exact redox potential values via SWV. The cell was operated using an AUTOLAB PGSTAT204 (Metrohm), monitored by the Metrohm NOVA © software. The working electrodes (d = 3 mm) were polished over a 1 μm alumina slurry, rinsed with water, sonicated in H_2_O and acetone, and then dried in a stream of N_2_. Electrodes were transferred directly into the glovebox. UVvis-SEC measurements were performed using a special SEC-C05T thin layer quartz glass cuvette cell (d = 0.5 mm, ALS) equipped with a Pt-mesh WE and Pt CE. The cell was put in an Avantes CUV-ALL-UVVIS cuvette holder connected to an Avantes AvaLight-DH-S-BAL source and AvaSpec-UL2048CL-EVO spectrometer. UVVis-measurements were operated by the AvaSoft 8 software package. During measurements, optical triggers were using to obtain spectra at every nth potential step at 1.25 ms integration time. All measurements were performed under N_2_ atm. inside a glovebox.

### Single-Crystal Structure Determination

Data collection was performed with a XtaLAB Synergy, Dualflex, HyPix diffractometer. The structure was solved with SHELXT and refined with SHELXL using least-squares minimization. All non-hydrogen atoms were refined anisotropically. The H atoms of C–H groups were positioned with an idealized geometry (methyl H atoms allowed to rotate but not to tip) and were refined isotropic with U_iso_(H) = 1.2 U_eq_(C) (1.5 for methyl H atoms) using a riding model. One of the two crystallographically independent anions is disordered and is refined by using a split model. The position of the dichloromethane solvate molecule was not fully occupied. After structure refinement, there is one residual electron density maximum indicating for one additional water molecule that is disordered and for which no reasonable structure model and no H atoms were found. Therefore, its contribution to the electron density map was removed.

CCDC-2545155 contains the supplementary crystallographic data for this paper. These data can be obtained free of charge from the Cambridge Crystallographic Data Centre via https://www.ccdc.cam.ac.uk/data_request/cif.

### Synthesis

The ligand (2,2′-dipyridyl)­methyl-(2-pyridyl)­methyl-(3-phenylazo-6-pyridyl)­methylamine (3AzoN4Py, **1**) and the iron complex [Fe­(3AzoN4Py)­(MeCN)]­(BF_4_)_2_ (**1-Fe** ^**II**^)^35^ as well as the salts (cobalt­(II)­bistrifluoromethylsulfonyl)­imide (**2**)^76^ and (silver­(I)­bistrifluoromethylsulfonyl)­imide (**3**)^77^ were synthesized according to literature procedures. The synthesis of the complexes **1-Zn** ^**II**^ and **1-Co** ^**III**^ are detailed in the Supporting Information.

## Supplementary Material



## Notes

Synthetic procedures, crystallographic data, spectroscopic and electrochemical data, and DFT calculations (PDF)

## Abbreviations

TDDFT: Time-dependent density functional theory.
ESA: Excited-state absorption.
GSB: Ground-state bleach.
EADS: Evolution-associated difference spectra.
TEAS: Time-resolved electron absorption spectroscopy.
MC: Metal-centered.
LC: Ligand-centered.
LMCT: Ligand to metal charge transfer.
MLCT: Metal to ligand charge transfer.
CV: Cyclic voltammetry.
LD-LISC: Ligand-driven light-induced spin change.
LD-CISSS: Light-driven coordination-induced spin-state switching.
IRF: Instrument response function.
