# Supplementary material for: Influence of Transition-Metal Ions on the Photoisomerization of a Pentadentate Azopyridine Ligand: Interplay of Charge-Transfer and Metal-Centered States
Source: Inorg Chem. 2026 Aug 19;65(34):19918–31. doi: 10.1021/acs.inorgchem.6c02962 (PMC13536887; doi:10.1021/acs.inorgchem.6c02962)
Supplement: Supplementary file 1 [file ic6c02962_si_001.pdf]

Supporting Informtation

Influence of Transition Metal Ions on the Photoisomerization of a Pentadentate Azopyridine Ligand: Interplay of Charge-Transfer and Metal-Centered States

Sebastian Megow,,†# Lydia Adam,‡# Niels Michaelis,‡# Annika Prax⁑, Henrike-Leonie Fitschen,‡ Christian Näther,‡ Friedrich Temps⁑ and Felix Tuczek*,‡*

‡Institut für Anorganische Chemie, Christian-Albrechts-Universität zu Kiel, Max-Eyth-Straße 2, 24118 Kiel, Germany

†Institut für Physik, Freie Universität Berlin, Arnimallee 14, 14195 Berlin, Germany

⁑ Institut für Physikalische Chemie, Christian-Albrechts-Universität zu Kiel, Max-Eyth-Straße 1, 24118 Kiel, Germany

AUTHOR INFORMATION

# These authors contributed equally to the manuscript.

Corresponding Authors

*E-mail: ftuczek@ac.uni-kiel.de

*E-mail: sebastian.megow@fu-berlin.de

Supporting Information

Content

Synthesis 2
Complex [Co(3AzoN4Py)(MeCN)](NTf)$_3$ (1-Co^{III}) 4
Complex [Zn(3AzoN4Py)(MeCN)](BF$_4$)$_2$ (1-Zn^{II}) 6
Analytical data 9
Crystal data 9
^{1}H-NMR spectroscopy to determine the *trans:cis*-ratios before and after irradiation 10
Transient Absorption Spectroscopy 13
Electrochemistry 16
UV/vis-Spectro Electrochemistry 25
Steady-State Emission Spectra 29
DFT calculations 29
References 31

Synthesis

The syntheses were performed under nitrogen atmosphere, unless mentioned otherwise. When working under nitrogen atmosphere, standard Schlenk techniques were used, for working under argon atmosphere a M. Braun Labmaster 130 glovebox was used. All solvents and starting materials used were bought from commercial sources and used without purification, except when the solvents were marked with abs., then they were purified and dried by distillation using following drying reagents: Et_2O was dried over $LiAlH_4$ under Argon, Acetonitrile and DCM were dried over CaH_2 under N_2. All solvents were distilled prior to use.

NMR spectra were recorded at 298 K on a Bruker Avance 400 Pulse Fourier Transform spectrometer in deuterated solvents operating at a ^{1}H frequency of 400.1 MHz and ^{13}C frequency of 100.6 MHz.

All NMR spectra were referenced to the residual protonated solvent signal (^{1}H: δ($CHCl_3$) = 7.26 ppm; δ (CH_3CN) = 1.94 ppm), the solvent signal (^{13}C: δ ($CDCl_3$) = 77.16 ppm; δ (CH_3CN) = 1.32 ppm). With the help of DEPT-135 and two-dimensional correlation spectra ($^{1}H,^{1}H$-COSY, $^{1}H,^{13}C$-HSQC, $^{1}H,^{13}C$-HMBC) Signals were assigned. Signal multiplicities are shortened as s (singlet), d (doublet), t (triplet), m (multiplet) and br. (broad signal).

Infrared spectra were recorded with neat samples on a Bruker Alpha FT-IR Spectrometer with a Platinum ATR module in a range of 4000 to 400 cm^{-1} with a resolution of 1.5 cm^{-1}. Signal intensities are marked as s (strong), m (medium), w (weak), and br (broad).

Elemental analyses were measured using a Euro Vector CHNS-O element analyser (Euro EA 3000) from HEKAtech by burning the samples in an oxygen stream in a tin pot. High resolution ESI mass spectra were recorded on a ThermoFisher Orbitrap spectrometer.

The UV/vis spectra were recorded using the Cary 5000 UV/vis spectrometer from Varian in double-slit mode. The spectra were recorded in quartz cuvettes with a layer thickness of 1 cm in the specified solvents and with these as reference. The spectra were recorded in the range from 800 to 200 nm.

The ligand (2,2'-Dipyridyl)methyl-(2-pyridyl)methyl-(3-phenylazo-6-pyridyl)methylamine (3AzoN4Py, **1**) and iron complex [Fe(3AzoN4Py)(MeCN)](BF_4)$_2$ (**1-Fe^{II}**)[1] as well as the salts Cobalt(II)bistrifluoromethylsulfonyl)imide (**2**)[2] and Silver(I)bistrifluoromethylsulfonyl)imide (**3**)[3] were synthesized according to literature procedures.

Complex [Co(3AzoN4Py)(MeCN)](NTf)3 (1-Co^{III})

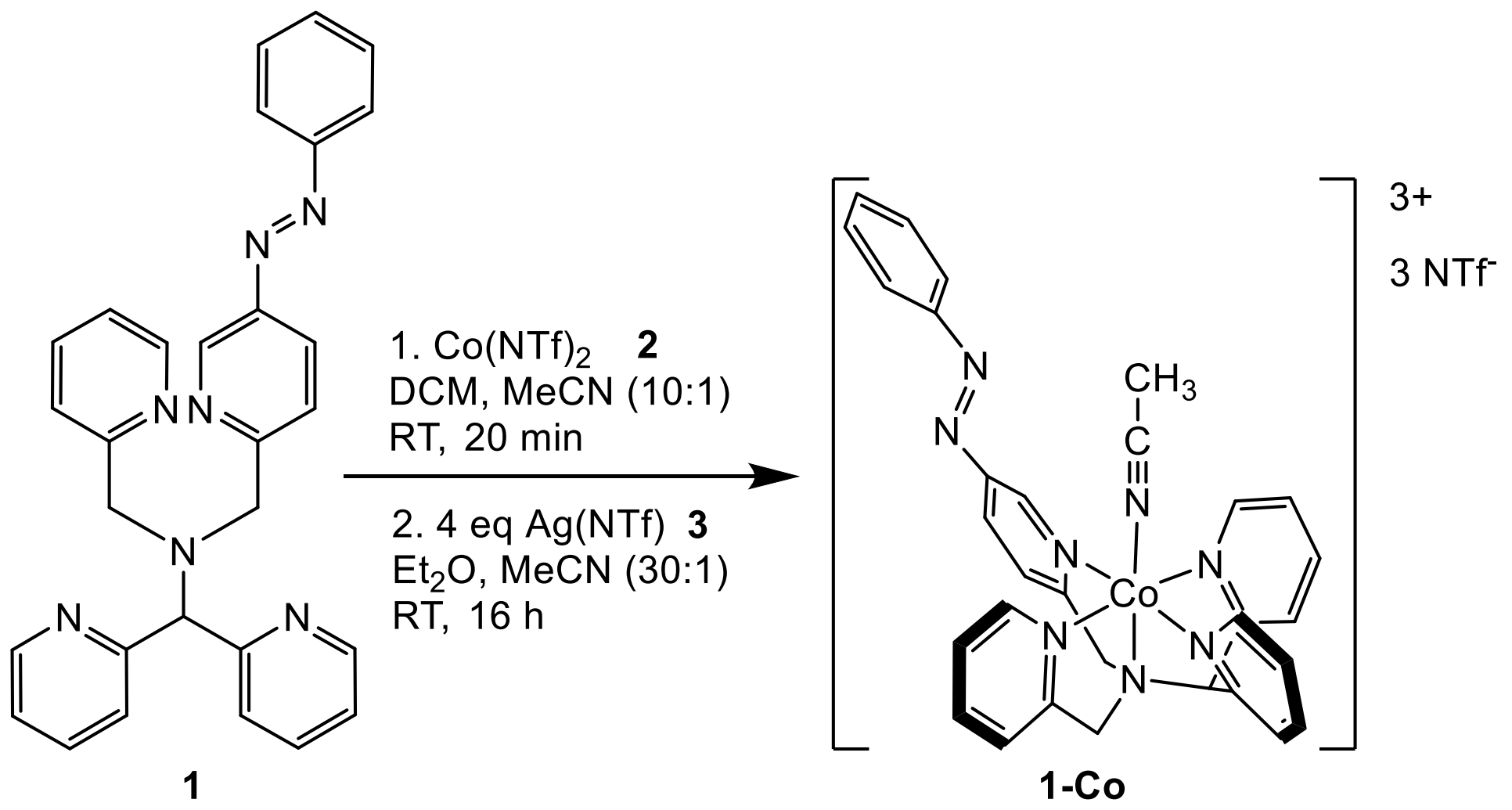

The complex [Co(3AzoN4Py)(MeCN)](NTf)3 **1-Co^{III}** was synthesized based on a publication of GOLDBERG *et al*[4] with modifications from a publication from YAMAGUCHI *et al*[5]. The synthesis was performed under N_2 inert gas atmosphere.

Cobalt(II)-triflimide (**2**, 61.9 mg, 100 µmol) was dissolved in 0.5 mL abs. acetonitrile. (2,2'-Dipyridyl)methyl-(2-pyridyl)methyl-(3-phenylazo-6-pyridyl)methylamine (3AzoN4Py, **1**, 47.2 mg, 100 µmol) was dissolved in 2 mL abs. dichloromethane, added to the solution of **2** and rinsed with 3 mL abs. dichloromethane. The red solution was stirred at room temperature for 20 min. after which the solvent was reduced to 1 mL, to which then 0.3 mL of abs. acetonitrile was added. Silver(I)-triflimide (**3**, 155 mg, 400 µmol) was solved in 5 mL of abs. diethylether, added to the reaction mixture and rinsed with another 5 mL of abs. diethylether. A yellow suspension formed and the reaction mixture was stirred at room temperature for 2 days.

Under air the solution was filtered through a syringe filter with a pore size of 0.45 µm. The solvent was evaporated in vacuum, but not removed to dryness. The resulting red oil was diluted with 10 mL. The solution was allowed to evaporate under standard pressure.

A dark orange solid was obtained, which was washed with 20 mL diethylether to remove excess Silver(I)-triflimide **3**. Next the raw product was washed with 150 mL dichloromethane until the filtrate came out colourless. The product **1-Co^{III}** was obtained as an orange solid. Crystals suitable for single-crystal-analysis were obtained by vapour diffusion of diethyl ether to a solution of 5 mg of the product **1-Co^{III}** in a mixture of 0.1 mL of acetonitrile and 1 mL of dichloromethane.

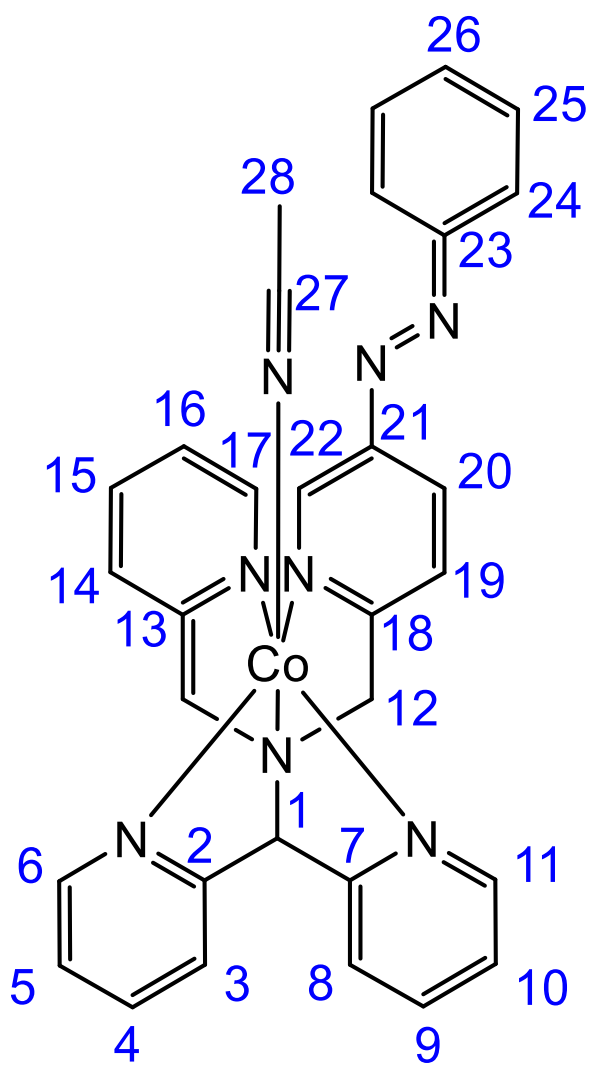

Yield: 80 mg (56.6 µmol, 57 %).

^{1}H-NMR (400 MHz, CD_3CN, 300 K): δ =9.44 (d, ^{4}J = 2.0 Hz, 1 H, *H*-22), 9.07 (d, ^{3}J = 6.0 Hz, 1 H, *H*-17), 8.78 (d, ^{3}J = 5.8 Hz, 1 H, *H*-6/11), 8.69 (d, ^{3}J = 5.8 Hz, 1 H, *H*-6/11), 8.38 (dd, ^{3}J = 8.5 Hz, ^{4}J = 2.0 Hz, 1 H, *H*-20), 8.29 (tt, ^{3}J = 7.7, ^{4}J = 1.5 Hz, 2 H, *H*-4, *H*-9), 8.25 – 8.21 (m, 2 H, *H*-3, *H*-8), 8.09 – 8.02 (m, 3 H, *H*-15, *H*-24.), 7.77 – 7.72 (m, 1 H, *H*-16), 7.71 – 7.65 (m, 5 H, *H*-5, *H*-10, *H*-25, *H*-26), 7.57 (d, ^{3}J = 8.6 Hz, 1 H, *H*-19), 7.43 (d, ^{3}J = 7.9 Hz, 1 H, *H*-14), 6.58 (s, 1 H, *H-1*), 5.12 – 4.90 (m, 4 H, *H*-12), 3.03 (s, 3 H, *H*-28) ppm.

^{13}C-NMR (101 MHz, CD_3CN, 300 K): δ = 165.78 (1 C, *C*-18), 164.58 (1 C, *C*-13), 158.53 (2 C, *C*-2, *C*-7), 155.93 (1 C, *C*-6/11), 155.62 (1 C, *C*-6/11), 152.96 (1 C, *C*-23), 152.21 (1 C, *C*-17), 148.97 (1 C, *C*-22), 144.80 (2 C, *C*-4, *C*-9), 142.90 (1 C, *C*-15), 137.68 (1 C, *C*-28), 134.42 (1 C, *C*-26), 133.14 (1 C, *C*-20), 130.67 (2 C, *C*-25), 129.35 (2 C, *C*-5, *C*-10), 129.16 (1 C, *C*-16), 127.37 (2 C, *C*-3, *C*-8), 126.45 (1 C, *C*-19), 125.83 (1 C, *C*-14), 124.25 (2 C, *C*-24), 81.94 (1 C, *C*-1), 68.74 (1 C, *C*-12), 7.22 (1 C, *C*-28) ppm.

HR-ESI ms (ESI+): m/z = 190.39183 (calculated: 190.39175) [M -3 NTf^{-}]$^{3+}$.

FT-IR (ATR): $\tilde{\upsilon}$ = 3125 (w), 3095 (w), 3062 (w), 3001 (w), 2938 (w), 2326 (w), 2305 (w), 1613 (w), 1486 (m), 1469 (m), 1454 (m), 1346 (s), 1327 (s), 1178 (s), 1131 (s), 1054 (s), 914

(m), 860 (w), 840 (w), 788 (s), 765 (s), 740 (m), 717 (w), 689 (m), 652 (m), 614 (s), 596 (s), 570 (s), 509 (s), 469 (m), 449 (m), 404 (m), 359 (w), 286 (w), 224 (m), 208 (m) cm^{-1}.

Raman (KBr): $\tilde{\upsilon}$ = 3092 (w), 3080 (w), 2935 (w), 2333 (m), 2307 (w), 1615 (m), 1593 (m), 1571 (w), 1488 (m), 1472 (s), 1445 (s), 1411 (m), 1316 (w), 1240 (w), 1185 (s), 1153 (s), 1129 (m), 1067 (w), 1052 (w), 1041 (w), 1003 (w), 743 (w) cm^{-1}.

Complex $[Zn(3AzoN4Py)(MeCN)](BF_4)_2$ (1-Zn^{II})

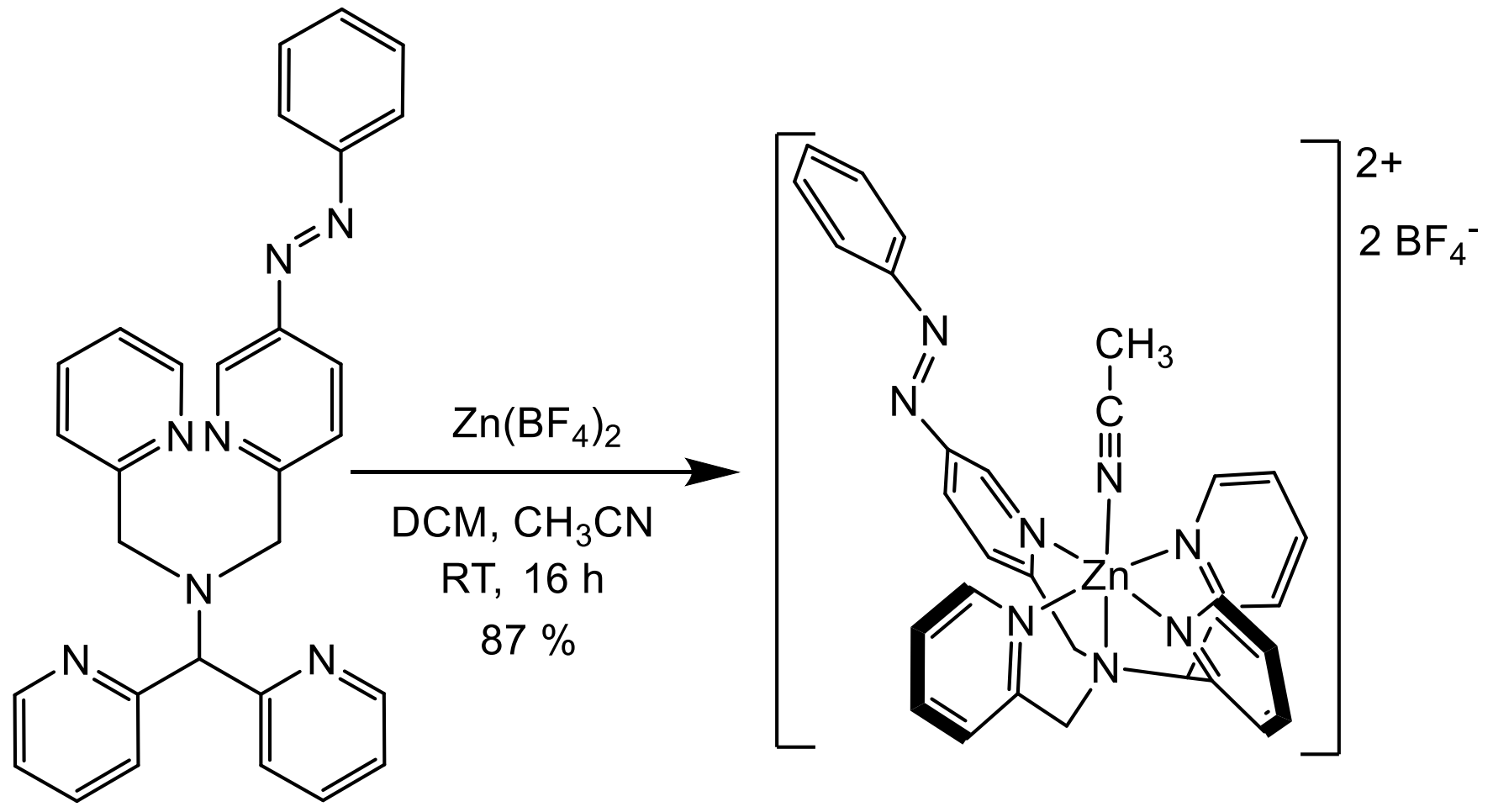

The complex $[Zn(3AzoN4Py)(MeCN)](BF_4)_2$ **1-Zn^{II}** was synthesized based on a publication of GOLDBERG *et al* [4], it was carried out under air atmosphere.

Zinc(II)-tetrafluoroborate hexahydrate was dried under vacuum. Then 25.1 mg (105 µmol) of the dried zinc(II)-tetrafluoroborate **(4)** were solved in 5 mL of acetonitrile and added to a solution of the ligand **1** (49.2 mg, 104.33 µmol) in 2 mL dichloromethane. The solution was stirred for 17 hours. Then under air atmosphere the solvents were allowed to evaporate under standard pressure, the resulting red oil was solved in 10 mL of dichloromethane and filtered through a syringe filter with a pore size of 0.45 µm. The solvent was allowed to evaporate under standard pressure again before the raw product was solved in 2 mL of acetonitrile, filtered through a syringe filter with a pore size of 0.45 µm. The solvent was evaporated under reduced

pressure at 30 °C resulting in a red oil to which 15 mL diethyl ether were added. The diethyl ether was again allowed to evaporate under standard pressure and the product **1-Zn^{II}** was obtained as an orange solid.

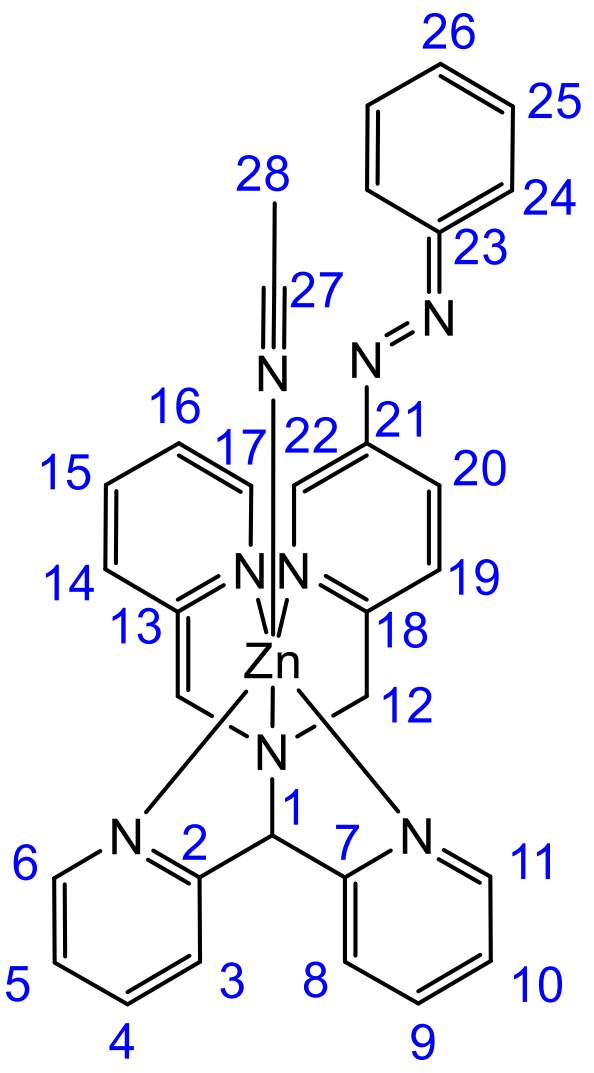

Yield: 64.2 mg (90.4 µmol, 87 %).

^{1}H-NMR (400 MHz, CD_3CN, 300 K): δ = 9.27 (^{4}d, J = 2.2 Hz, 1 H, *H*-22), 8.79 (ddd, ^{3}J = 5.3 Hz, ^{4}J =1.7 Hz, ^{5}J = 0.9 Hz, 1 H, *H*-17), 8.60 (dt, ^{3}J = 5.1, ^{4}J = 1.2 Hz, 1 H, *H*-6/11), 8.57 (dt, ^{3}J = 5.1, ^{4}J = 1.4 Hz, 1 H, *H*-6/11), 8.16 (dd, ^{3}J = 8.5, ^{4}J = 2.2 Hz, 1 H, *H*-20), 8.01 – 7.96 (m, 2 H, *H*-4, *H*-9), 7.96 – 7.93 (m, 2 H, *H*-24), 7.83 – 7.79 (m, 3 H, *H*-3, *H*-8, *H*-15), 7.64 – 7.60 (m, 3 H, *H*-25, *H*-26), 7.48 – 7.43 (m, 3 H, *H*-5, *H*-10, *H*-16), 7.37 (d, ^{3}J = 8.4 Hz, 1 H, *H*-19), 7.23 (d, ^{3}J = 7.8 Hz, 1 H, *H*-14), 6.11 (s, 1 H, *H*-1), 4.52 – 4.20 (m, 4 H, *H*-12). ppm.

^{13}C-NMR (101 MHz, CD_3CN, 300 K): δ = 157.10, 155.10, 154.73, 153.06, 149.57, 149.48, 147.53, 144.95, 141.92 (d, J = 2.7 Hz), 140.96, 133.59, 130.71, 130.49, 126.68, 126.00, 125.62, 125.50, 124.96, 123.87, 71.00, 59.23, 59.19.ppm.

HR-ESI ms (ESI+): m/z = 267.57242 (calculated: 267.57260) [M -CH_3CN -2 BF_4^-]$^{2+}$.

FT-IR (ATR): $\tilde{\upsilon}$ = 3075 (w), 2929 (w), 2861 (w), 1605 (m), 1572 (w), 1481 (w), 1471 (w), 1442 (m), 1400 (w), 1377 (w), 1356 (w), 1282 (w), 1157 (w), 1038 (br. s), 913 (m), 871 (m), 843 (m), 762 (s), 688 (s), 646 (m), 583, (m), 563 (m), 516 (s), 410 (s) cm^{-1}.

Raman (KBr): $\tilde{\upsilon}$ = 3072 (w), 2940 (w), 1593 (m), 1488 (m), 1468 (s), 1447 (s), 1405 (w), 1382 (w), 1312 (w), 1185 (s), 1153 (s), 1052 (w), 1022 (w), 1000 (w) cm^{-1}.

Tab. S1: Comparison of the M-N bond lengths in Fe(II)(N4Py), Co(III)(N4Py) and **1-Co^{III}**.

Complex cation	Metal-nitrogen donor bond lengths/Å					
	M-N_3 (N_{Amine})	M-N_1 ($N_{Py\text{-}CH}$)	M-N_2 ($N_{Py\text{-}CH}$)	M-N_4 ($N_{Py\text{-}CH2}$)	M-N_5 ($N_{Py\text{-}CH2,\ Azo\ carrying}$)	M-N_8 ($N_{Py\text{-}MeCN}$)
Fe(II)(N4Py)	1.961(3)	1.976(3)	1.967(3)	1.968(3)	1.975(3)	1.915(3)
Co(III)(N4Py)	1.918(3)	1.936(3)	1.929(3)	1.922(3)	1.918(3)	1.910(3)
**1-Co^{III}**	1.924(2)	1.940(3)	1.930(3)	1.923(3)	1.934(3)	1.908(2)

Analytical data

Crystal data

Tab. S2: Crystal data and structure refinement for **1-Co^{III}.**

Empirical formula	$C_{37.92}H_{29.84}Cl_{1.84}CoF_{18}N_{11}O_{12}S_6$
Formula weight	1490.12
Temperature/K	100.00(10)
Crystal system	triclinic
Space group	P-1
a/Å	11.86526(16)
b/Å	13.3248(2)
c/Å	19.56604(14)
α/°	90.6614(9)
β/°	102.3286(9)
γ/°	112.2284(14)
Volume/Å^3	2782.89(7)
Z	2
ρ_{calc}g/cm^3	1.778
μ/mm^{-1}	6.514
F(000)	1493.0
Crystal size/mm^3	0.25 × 0.25 × 0.04
Radiation	Cu Kα (λ = 1.54184)
2Θ range for data collection/°	7.204 to 161.708
Index ranges	-15 ≤ h ≤ 15, -17 ≤ k ≤ 16, -24 ≤ l ≤ 18
Reflections collected	37452
Independent reflections	11742 [R_{int} = 0.0278, R_{sigma} = 0.0226]
Reflections with [I>=2σ (I)]	11291
Data/restraints/parameters	11742/156/840
Goodness-of-fit on F^2	1.046
Final R indexes [I>=2σ (I)]	R_1 = 0.0589, wR_2 = 0.1680
Final R indexes [all data]	R_1 = 0.0599, wR_2 = 0.1689
Largest diff. peak/hole / e Å^{-3}	1.78/-0.97

^{1}H-NMR spectroscopy to determine the *trans:cis*-ratios before and after irradiation

General procedure:

The ligand 1 and the complexes **1-Zn^{II}**, **1-Fe^{II}** and **1-Co^{III}** were solved in deuterated acetonitrile. The ^{1}H-NMR spectra for the PSS without irradiation was measured first. Next the NMR-tube with the respective solution was transferred to a setup that allows for the NMR-tube to be rotated and cooled with an air flow while being irradiated by either four high-power LED at 340 nm or four high-power LED at 440 nm. While transferring the probe to the NMR-spectrometer, the NMR-tube was covered with aluminium foil to suppress influence of other light sources.

First the ^{1}H-NMR spectra for the PSS at 340 nm were recorded after irradiation for 60 minutes. Immediately after the measurement was finished, the probe was brought to the PSS at 440 nm via irradiation for 60 minutes and the respective ^{1}H-NMR spectra were recorded. To determine the ratio of *trans:cis*-isomer the integrals of the signal corresponding to the *H*-1 atom was compared. This is the aliphatic C-*H*-group of the ligand, which was chosen because there are no overlapping signals of the isomers at the ppm-range of this signal in any of the investigated molecules. The integrals were measured in the same ppm-range for all the spectra of the corresponding molecule.

Tab.S3: Integral Specifications for the trans- and cis-isomers of the four molecules **1**, **1-Zn^{II}**, **1-Fe^{II}** and **1-Co^{III}**.

molecule	*trans*-isomer integral start (ppm)	*trans*-isomer integral end (ppm)	*cis*-isomer integral start (ppm)	*cis*-isomer integral end (ppm)
**1**	5.350	5.310	5.240	5.190
**1-Zn**	6.130	6.100	6.060	6.030
**1-Fe**	6.387	6.360	6.300	6.260

**1-Co**	6.612	6.551	6.510	6.488

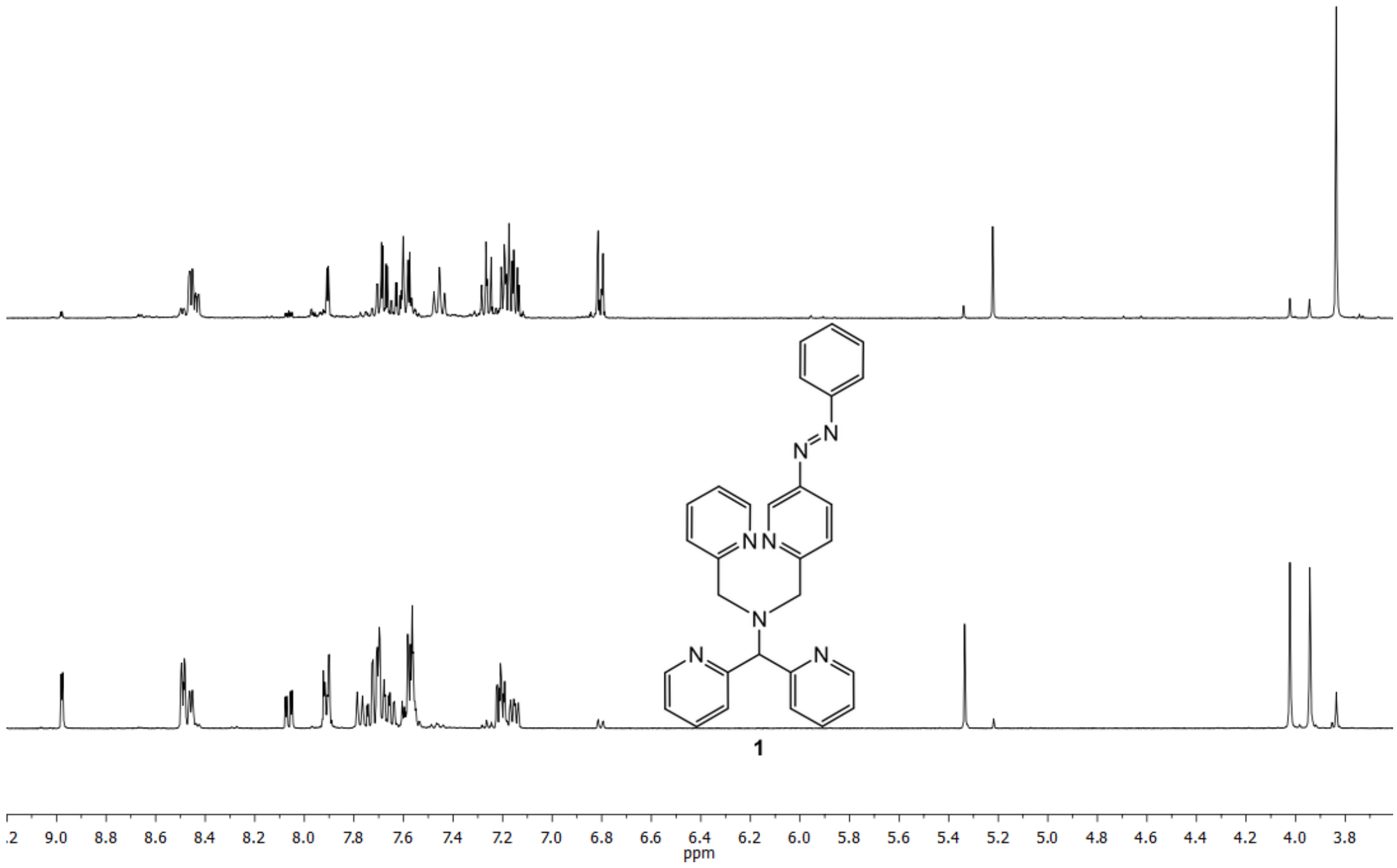

Fig. S1: ^{1}H-NMR spectra of the ligand **1** without irradiation (top) after 1 h irradiation with 340 nm (bottom).

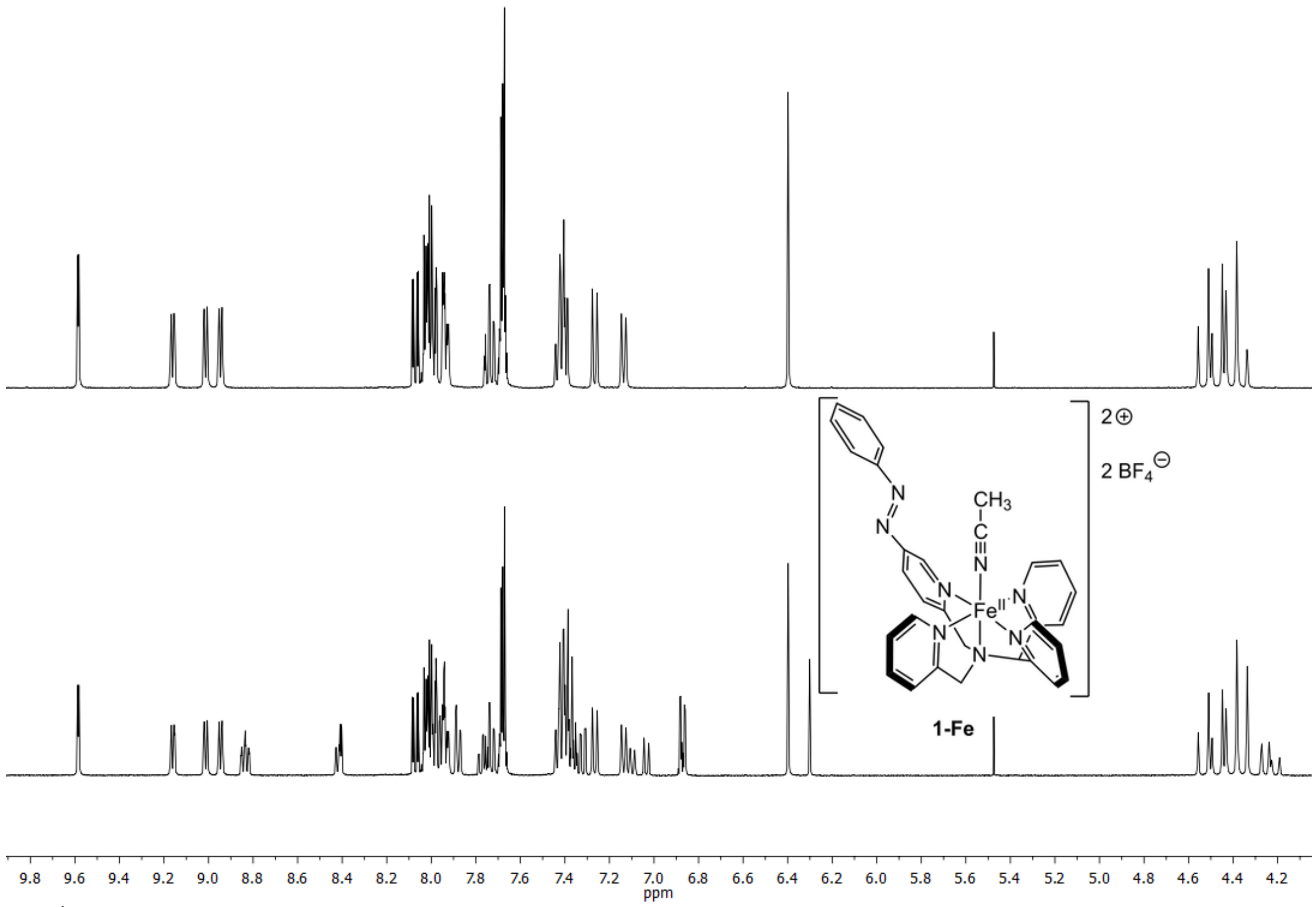

Fig. S2: ^{1}H-NMR spectra of the iron complex **1-Fe** without irradiation (top) after 1 h irradiation with 340 nm (bottom).

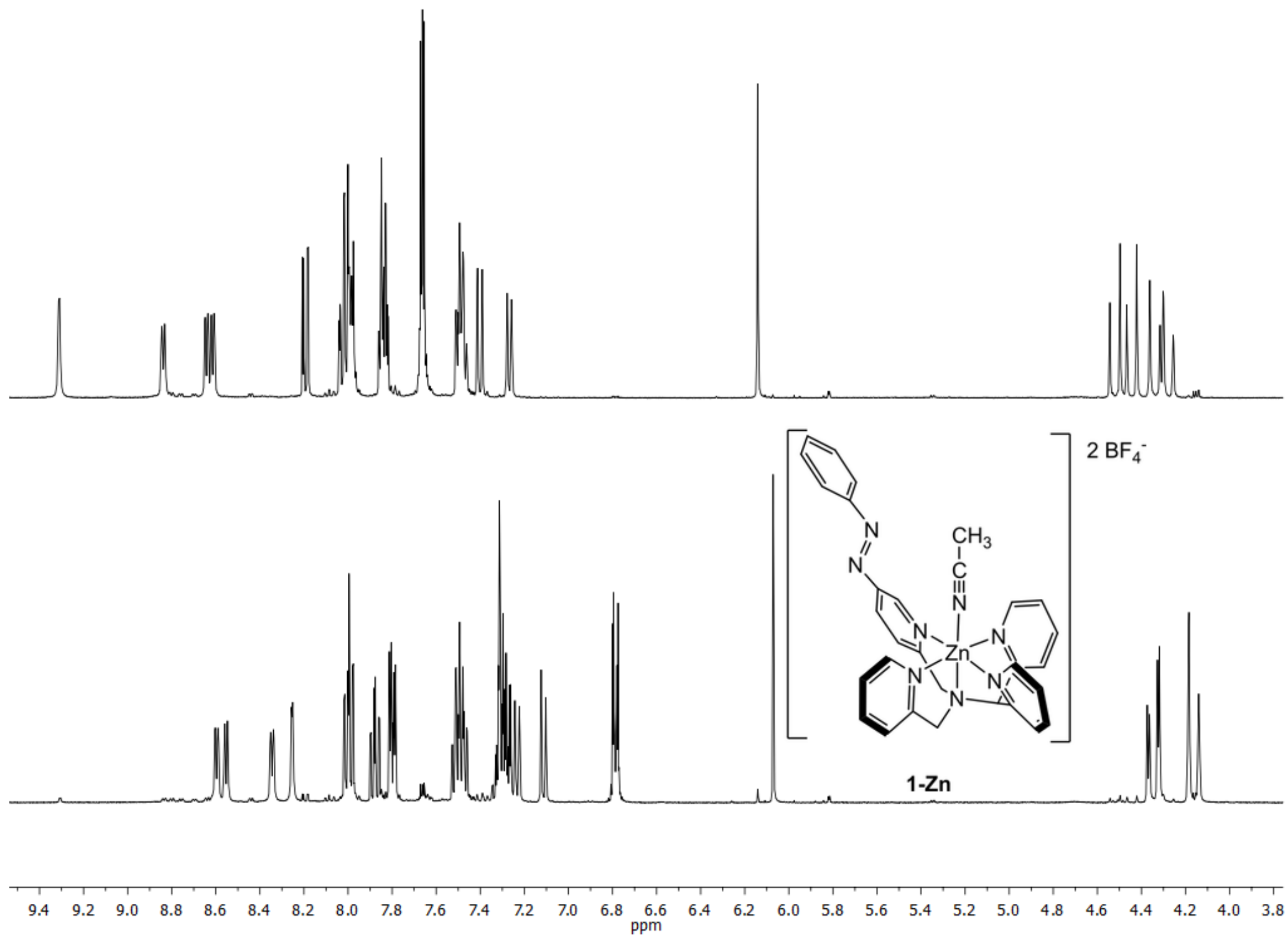

Fig. S3: ^{1}H-NMR spectra of the zinc complex **1-Zn** without irradiation (top) after 1 h irradiation with 340 nm (bottom).

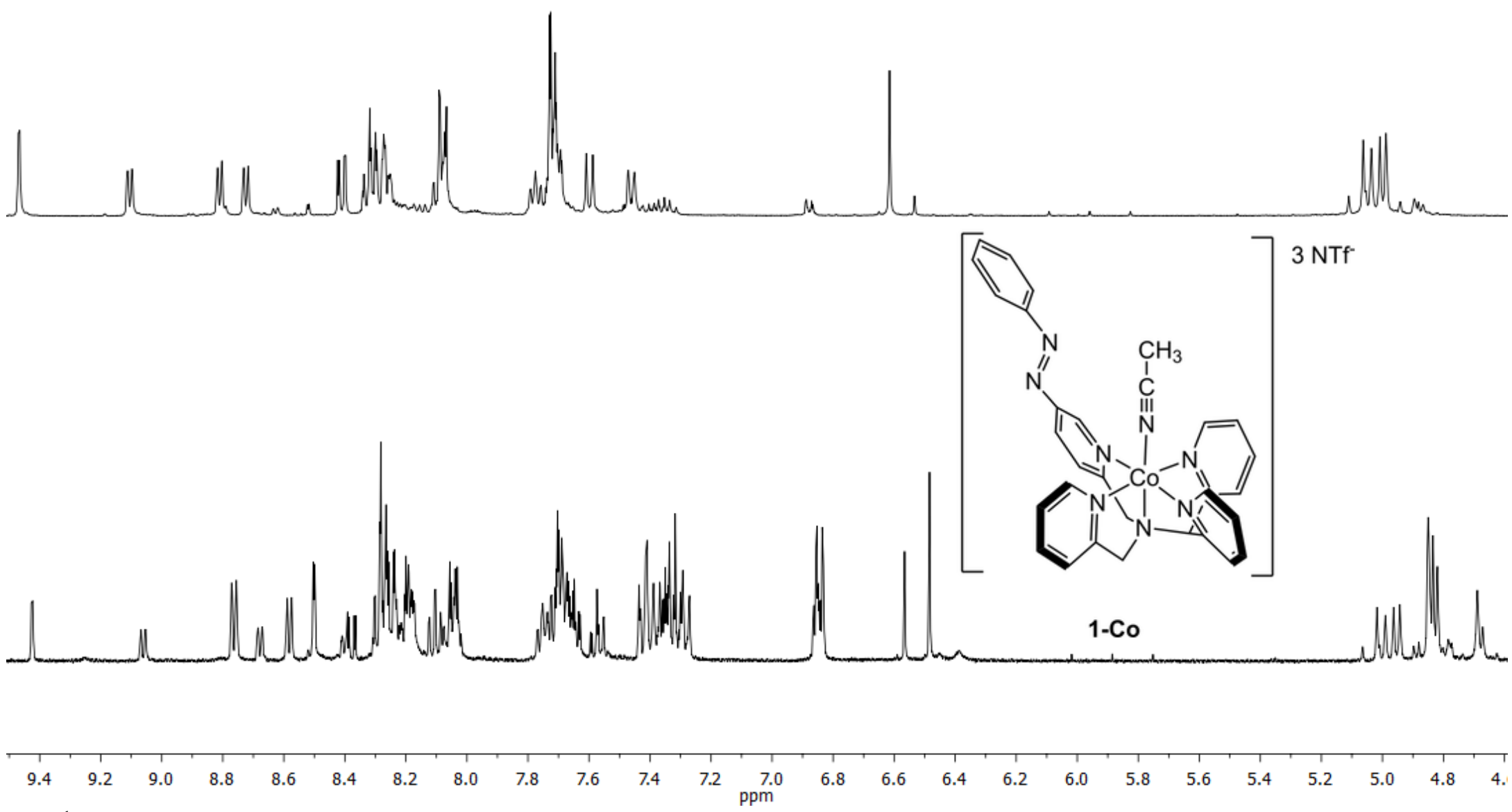

Fig. S4: ^{1}H-NMR spectra of the cobalt complex **1-Co** without irradiation (top) after 1 h irradiation with 340 nm (bottom).

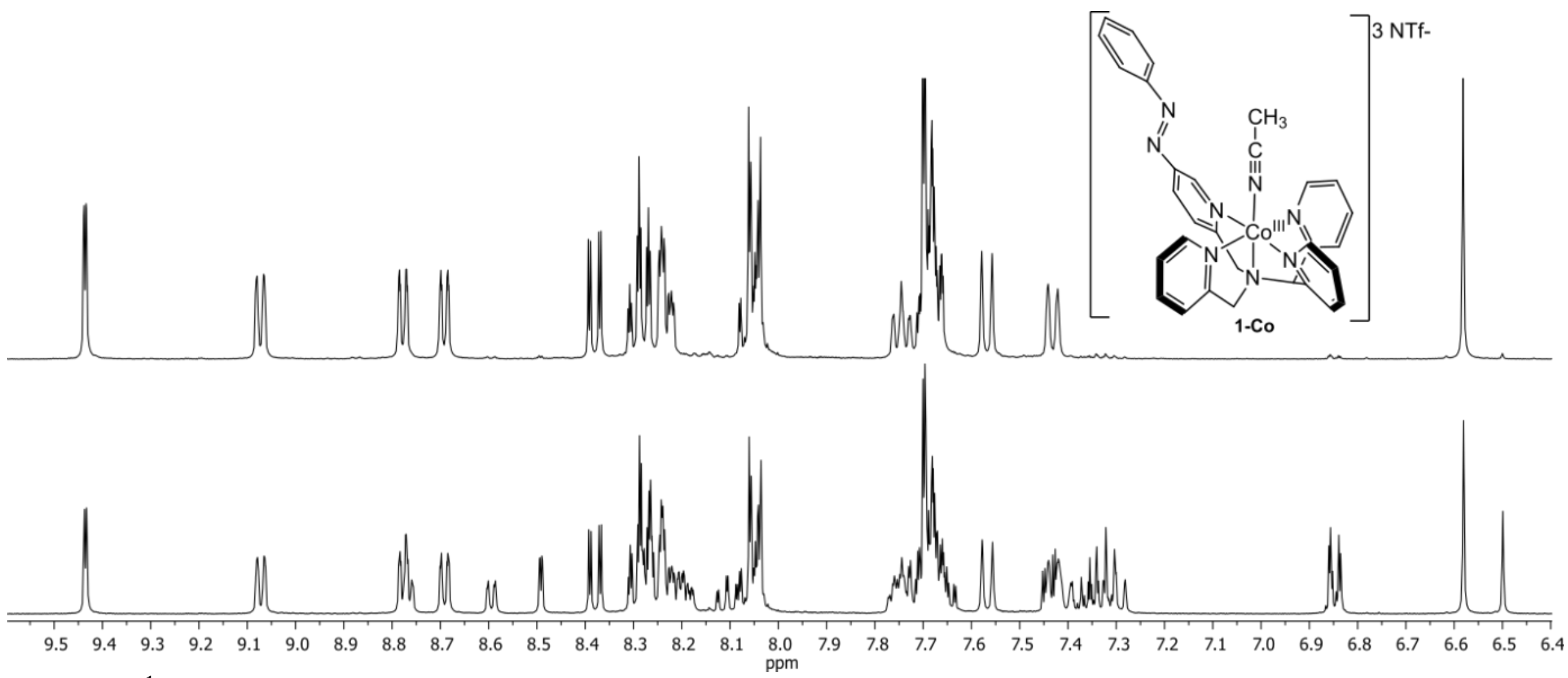

Fig. S5: ^{1}H-NMR spectra of the cobalt complex **1-Co** without irradiation (top) after 0.5 h irradiation with 365 nm (bottom). The *trans:cis*-ratios are 1:0.02 before and 1:0.50 after irratiation.

Molar extinction Coefficients

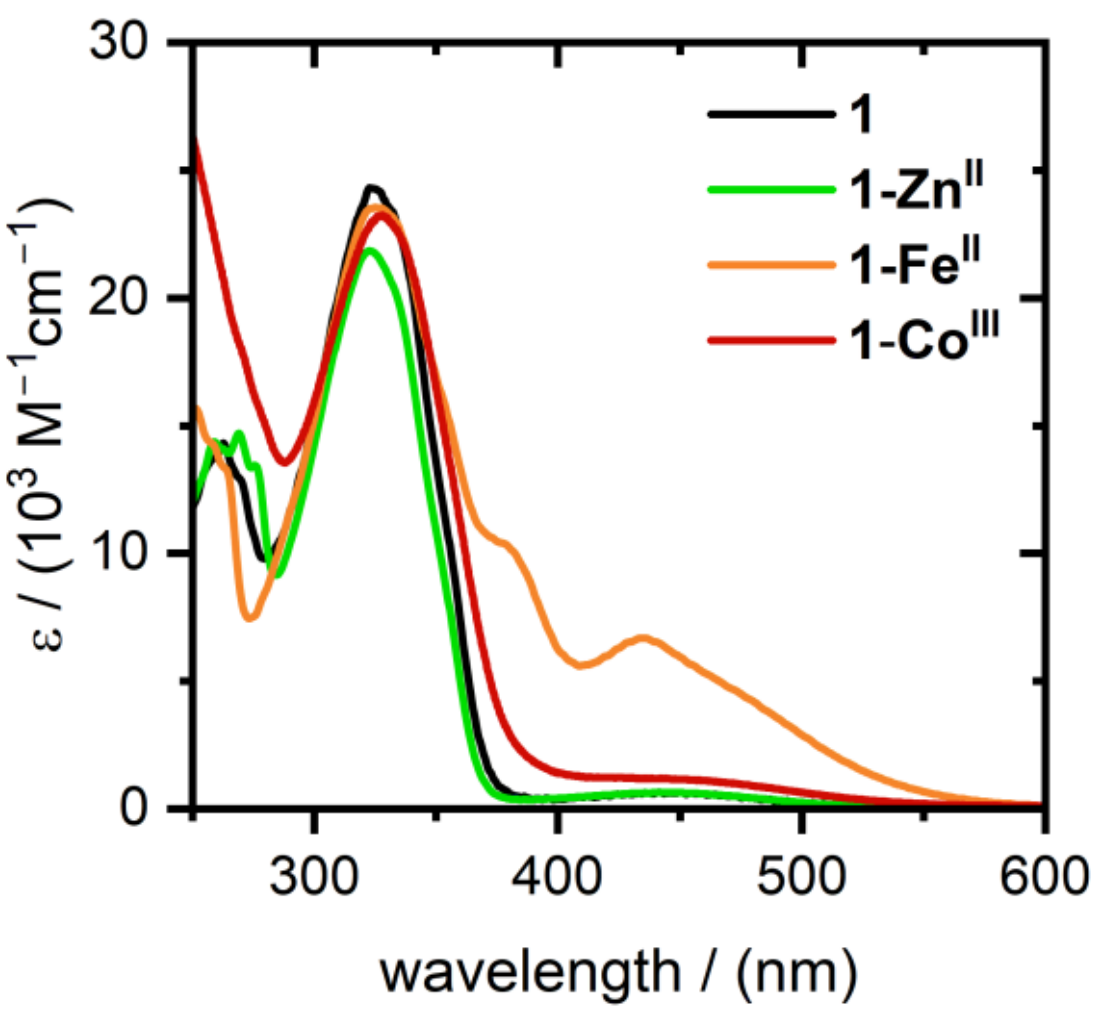

Fig. S6: Molar extinction coefficients of **1**, **1-Zn^II^**, **1-Fe^II^** and **1-Co^III^** in acetonitrile. Spectra were recorded immediately after preparation of the solutions under dark conditions to minimize the fraction of the *cis*-azopyridine isomer.

Transient Absorption Spectroscopy

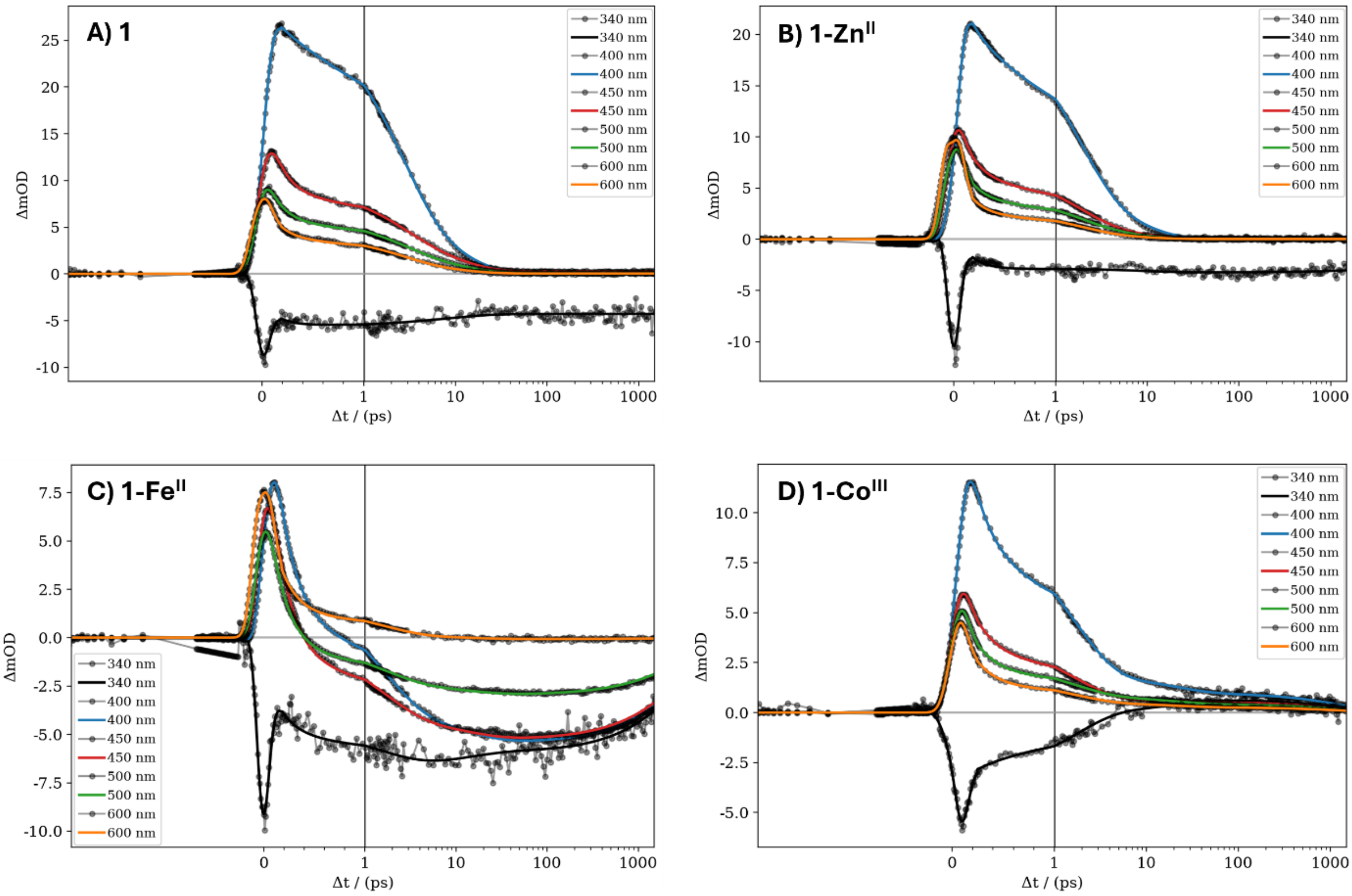

Fig. S7: Kinetic traces (dots) and global fits (solid lines) for selected probe wavelengths of A) **1**, B) **1-Zn**$^{\mathbf{II}}$, C) **1-Fe**$^{\mathbf{II}}$ and D) **1-Co**$^{\mathbf{III}}$ after photoexcitation at λ_{pump} = 315 nm in acetonitrile. The globally fitted IRF parameter σ_{IRF} is ~0.09 ps for all measurements.

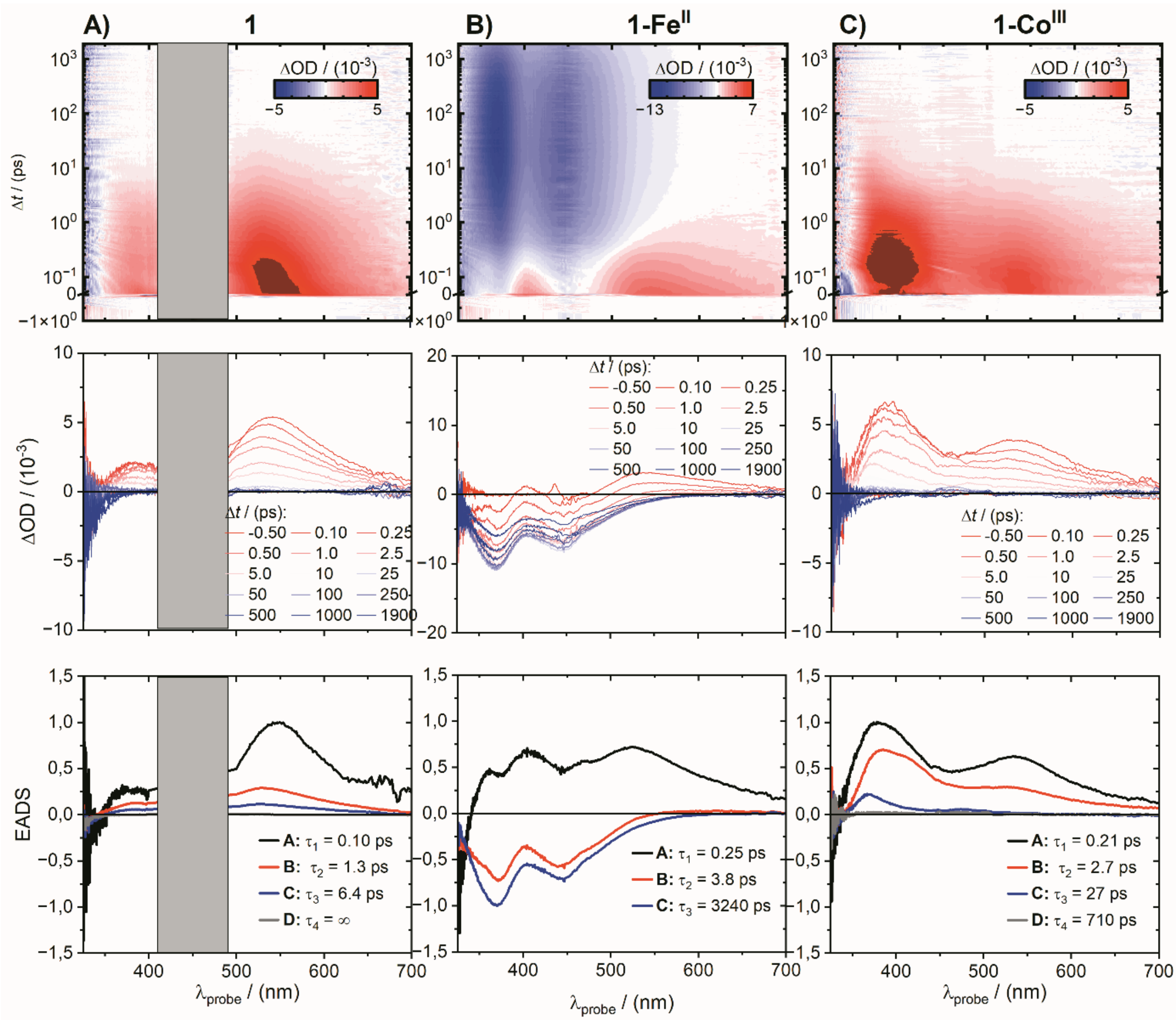

Fig. S8: Two-dimensional spectro-temporal absorption maps, transient absorption spectra and evolution-associated difference spectra (EADS) with the corresponding time constants obtained from a global analysis of a) **1**, b) **1-Fe^{II}** and c) **1-Co^{III}**. The data were recorded in acetonitrile following photoexcitation at λ_{pump} = 460 nm. For **1** the gray region was excluded from data analysis due to excessive pump scattering.

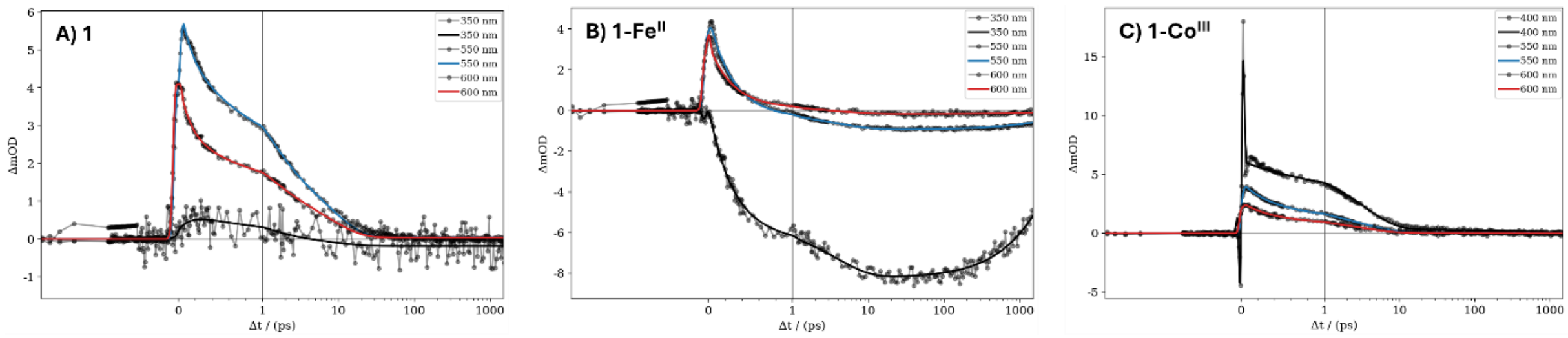

Fig. S9: Kinetic traces (dots) and global fits (solid lines) for selected probe wavelengths of A) **1**, B) **1-Fe^{II}** and C) **1-Co^{III}** after photoexcitation at λ_{pump} = 460 nm in acetonitrile. The globally fitted IRF parameter σ_{IRF} is ~0.03 ps for all measurements.

Electrochemistry

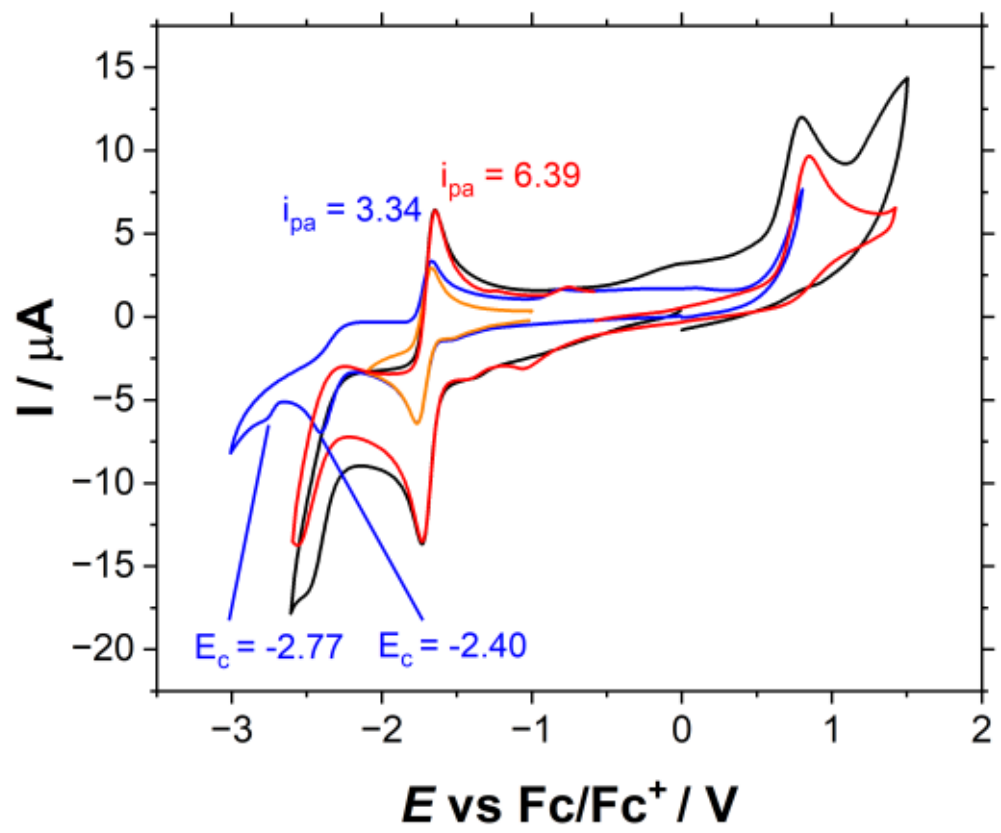

Fig. S10: Survey scans of 1 in different electrolytes and solvents: 1 in 50 mM TBANTf2 in abs. MeCN (red), 1 in 100 mM TBAPF6 in abs. MeCN (black), 1 in 100 mM TBAPF6 in abs. THF (blue, orange).

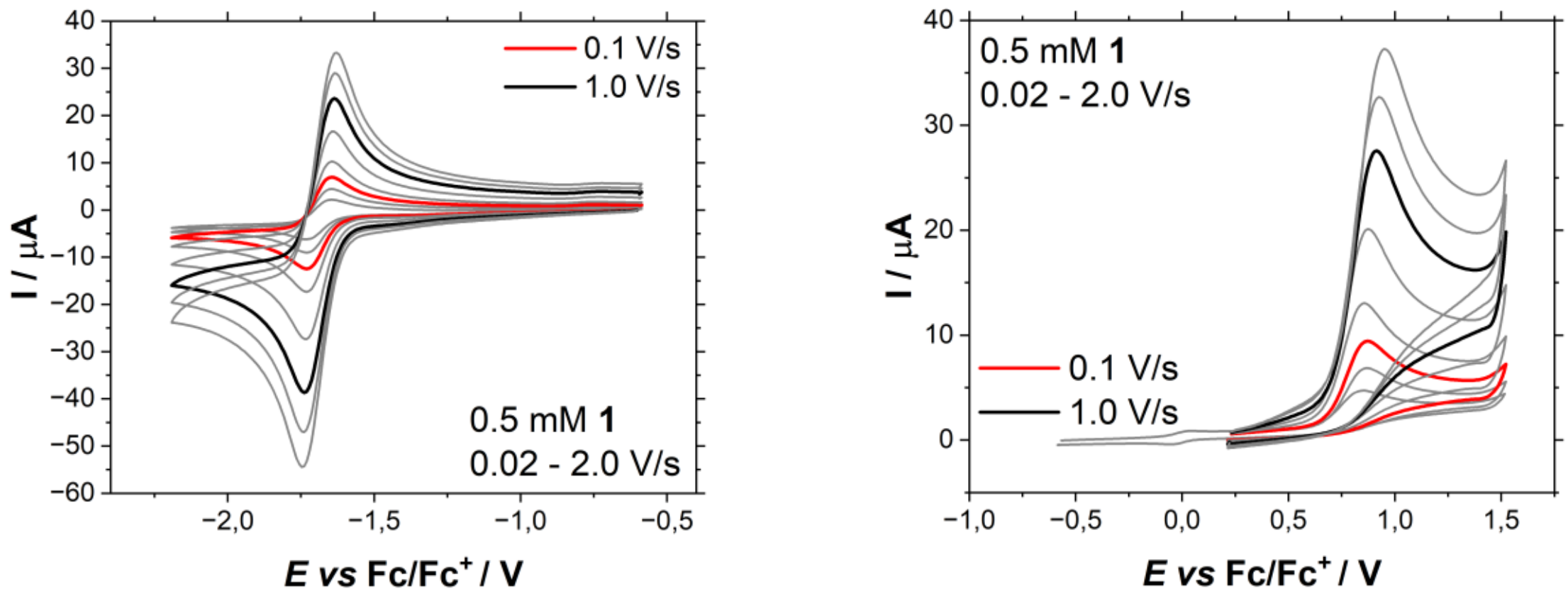

Fig. S11: Cyclic voltammetry of **1** at different scanrates 0.02 V/s ≤ v ≤ 2.0 V/s for both redoxsystems.

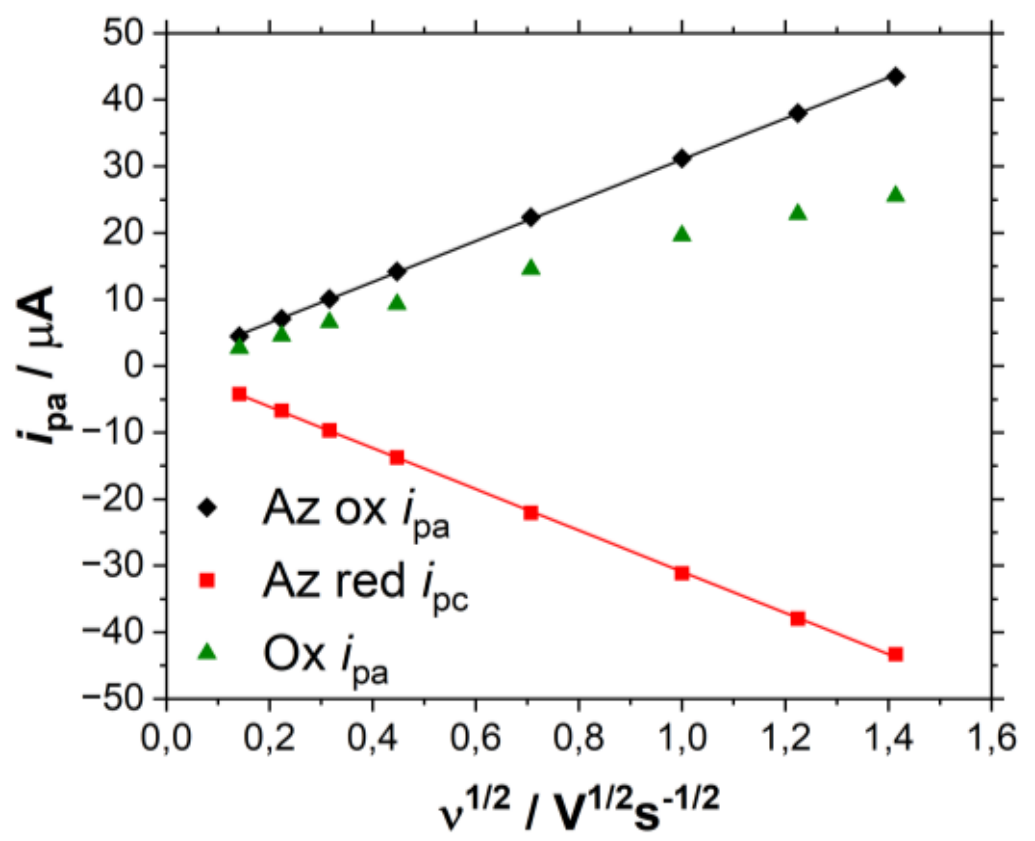

Fig. S12: Plots of i_{pa} and i_{pc} vs $v^{1/2}$ for both redox events of **1**.

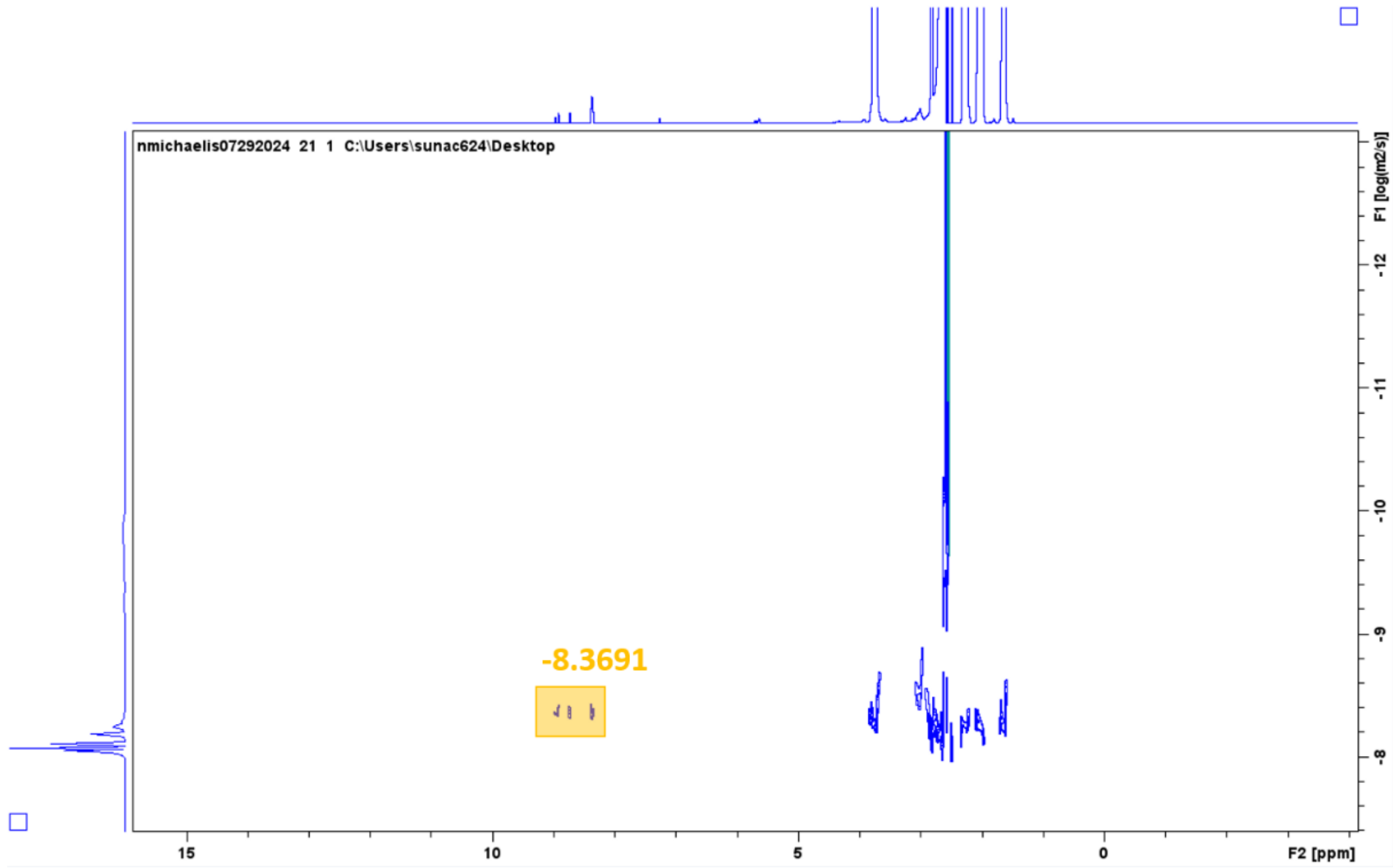

Fig. S13: ^{1}H-DOSY-NMR of **1** in 50 mM TBANTf$_2$ giving a diffusion coefficient of $D = 4.495 \cdot 10^{-9}$ m^2/s in MeCN.

Scan-rate variation between 0.02 to 2.0 V/s and subsequent plotting of i_{pa} and i_{pc} vs $v^{1/2}$ revealed a diffusion-controlled process for both major redox events (cf. Figure S11, S12). Using the Randles-Ševčik-equation[6] ($D = 4.495 \cdot 10^{-9}$ m^2/s, cf. Figure S13), we were able to determine the number of transferred electrons for the reversible redox-system at -1.68 V to *two*. Azo-compounds are known to undergo two one-electron reductions which are tied to the $[N{=}N]^{0/-1}$ and $[N{-}N]^{-1/-2}$ redox-states.[7] A direct two electron reduction to the $[N{-}N]^{2-}$ state can be coupled to a protonation step yielding a $[NH{-}N]^{-}$ - hydrazo-species and is favored in protic solvents.[8]

However, reversible one-step two electron reduction has been reported for the chemical similar azopyridine-family in abs. MeCN.[9] To probe whether the observed redox activity is coupled to the electrolyte anion NTf_2^- promoting protonation of the [N=N]-moiety, additional experimental conditions were tested, with CVs of **1** in 100 mM $TBAPF_6$/abs. MeCN showing no change of the electrochemical response of the system (cf. Figure S10). Changing the solvent from abs. MeCN to abs. THF not only widened the potential window to more negative potentials, the observed i_{pc} and i_{pa} currents were roughly two times lower compared to our results obtained in abs. MeCN. Additionally, a reduction peak at -2.40 V appeared, which has previously been masked in MeCN. These results indicate that MeCN supports the one-step/two-electron reduction of the azo-moiety, whereas a one-electron reduction is preferred in THF. Based on these experimental results we can identify the $[N{=}N]^{0/-2}$ redox-couple at $E_{1/2}$ = -1.68 V, separating the electrochemical response of **1** from comparable ligand designs by a more anodic $E_{1/2}$ and the capability to undergo reversible one-step/two-electron reduction. [5]

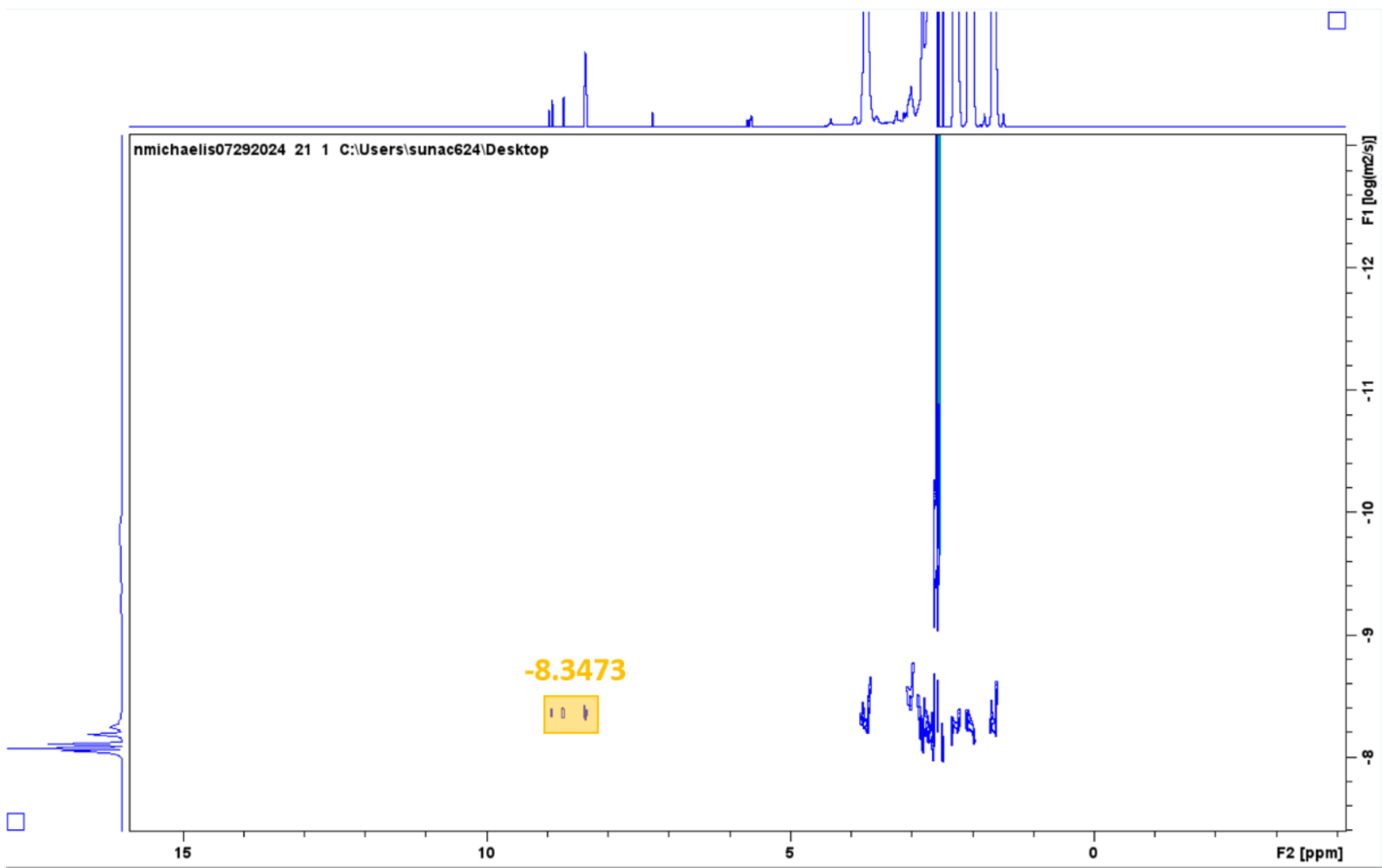

Fig. S14: ^{1}H-DOSY-NMR of **1-Co^{III}** in 50 mM $TBANTf_2$ giving a diffusion coefficient of $D = 4.275 \cdot 10^{-9}$ m^2/s in MeCN.

Tab. S4: Linear regressions for i_{pc} and i_{pc} fort he $[N{=}N]^{0/-2}$ redox-couple of **1**. Equations are based on y = ax + b.

	I_{pc} $[N{=}N]^{0/-2}$	I_{pa} $[N{=}N]^{0/-2}$
a	-3.097E-5 ± 1.869E-7	3.071E-5 ± 1.599E-7
b	8.459E-8 ± 1.531E-7	3.469E-7 ± 1.310E-7
P. R	-0.99989	0.99992
R^2 (COD)	0.99975	0.99984

Randles-Ševčik-equation at T = 300 K, c = 0.5 mM and electrode diameter r = 1.5 mm:

$$i_p = 0.4463\, n\, F\, A\, c \left(\frac{nFvD}{RT}\right)^{1/2}$$

$n(i_{pa}) = 2.26$; $n(i_{pc}) = 2.28$

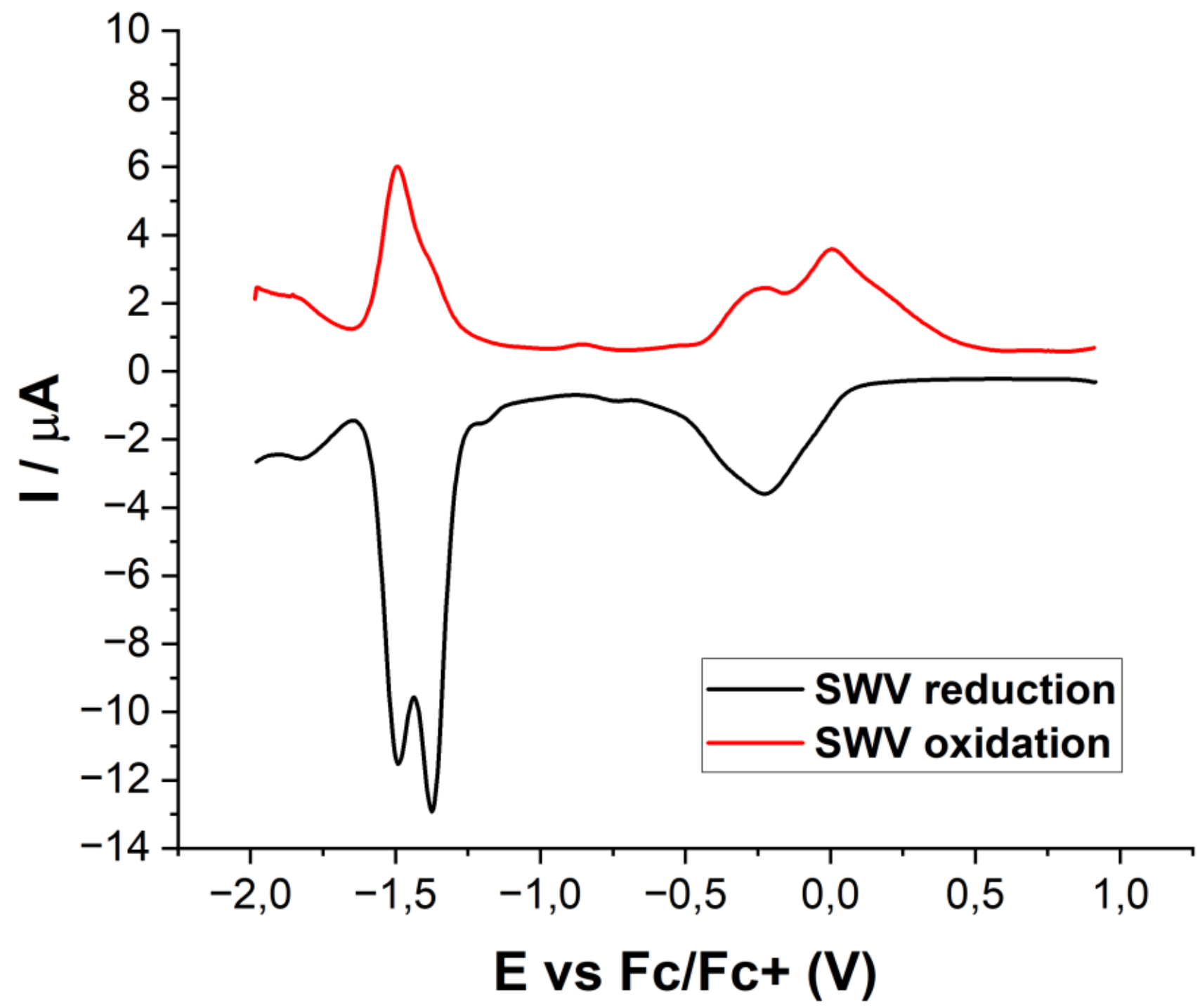

Fig. S15: Square-wave-voltammetry of 1 mM **1-Co**^{III} in 50 mM $TBANTf_2$ scanning from -2.0 V to 1.0 V (red) and vice versa (black).

We conducted measurements at different scan rates 0.02 V/s ≤ v ≤ 2.0 V/s with separate focus on $E_{(1)}$ and $E_{(2)}$, $E_{(3)}$ respectively. All data was corrected for the corresponding scan rate v and plotted as $iv^{-1/2}$ vs v in figure S16. The increase in v leads to a decrease in both i_{pc} values whith a cathodic shift for $E_{(2)}$ and $E_{(3)}$ of 77 mV and 35 V from -1.39 V to -1.47 V and -1.51 V to -1.55 V. Plots of $i_{pc}v^{-1/2}$ vs v revealed that the current response becomes diffusion limited at v ≥ 0.2 V/s for all three reduction peaks with approx. horizontal alignment of the corresponding peak maxima (cf. Figure S16-C).

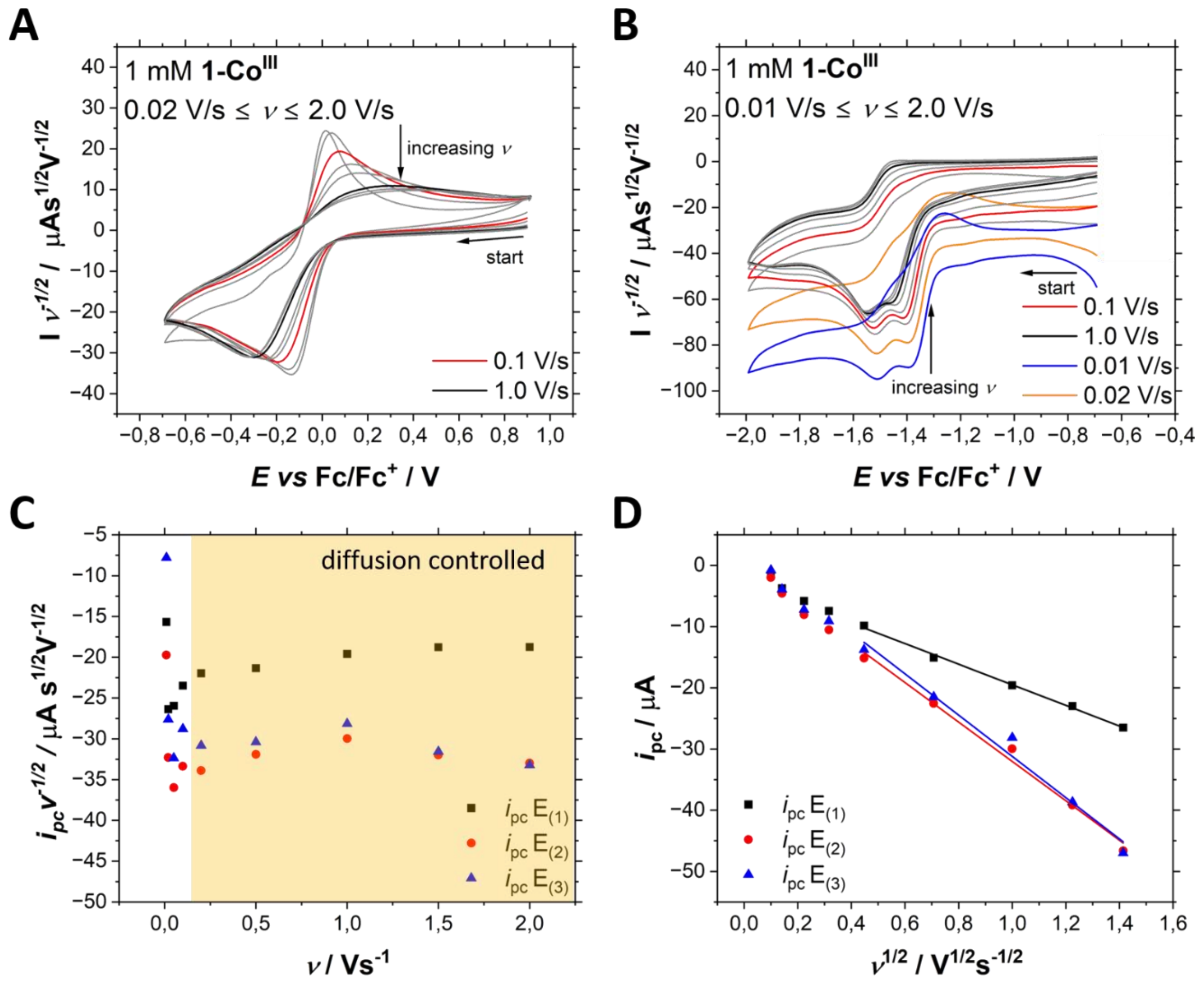

Figure S16: A: Plots of CVs at different scanrates for 0.02 V/s ≤ v ≤ 2.0 V/s between 0.8 and -0.6 V for $E_{(1)}$ of **1-Co^{III}**. Current has been corrected for $v^{-1/2}$. **B:** Plots of CVs at different scanrates for 0.02 V/s ≤ v ≤ 2.0 V/s with focus on $E_{(2)}$ and $E_{(3)}$ of **1-Co^{III}**. Current has been corrected for $v^{-1/2}$. **C:** Plots of the scanrate corrected current i_{pc} for $E_{(1)}$, $E_{(2)}$ and $E_{(3)}$ vs v for **1-Co^{III}**. **D:** Plots of i_{pc} vs $v^{1/2}$ for $E_{(1)}$, $E_{(2)}$ and $E_{(3)}$ of **1-Co^{III}**. Linear regressions for $E_{(1)}$ where applied in the reversible range for 0.02 V/s ≤ v ≤

0.1 V/s (grey dots) and the complete 0.02 V/s ≤ v ≤ 2.0 V/s range (black line). Linear regression for $E_{(2)}$ and $E_{(3)}$ was restricted to the diffusion controlled range of 0.5 V/s ≤ v ≤ 2.0 V/s.

Tab. S5: Linear regressions for i_{pc} of $E_{(1)}$, $E_{(2)}$ and $E_{(3)}$ of **1-Co^{III}**. Equations are based on y = mx + a.

	$E_{(1)}$ (0.2 – 2.0 V/s)	$E_{(2)}$ (0.2 – 2.0 V/s)	$E_{(3)}$ (0.2 – 2.0 V/s)
a	-2.6657E-6 ± 5.3877E-7	-1.73518-7 ± 1.9571E-6	-2.51355E-6 ± 2.8504E-6
m	-1.68325E-5 ± 5.28317E-7	-3.21994E-5 ± 1.9191E-6	-3.37211E-5 ± 2.79503E-6
P. R	-0.99853	-0.99471	-0.98985
R^2 (COD)	0.99705	0.98946	0.97981

Reversible Randles-Ševčik-equation at T = 300 K, c = 1.0 mM and electrode diameter r = 1.5 mm:

$$i_p = 0.446\, n\, F\, A\, c\, \left(\frac{nFvD}{RT}\right)^{1/2}$$

$E_{(1)}$ [i_{pc}] = 0.97; $E_{(2)}$ [i_{pc}] = 1.50; $E_{(3)}$ [i_{pc}] = 1.54

Irreversible Randles-Ševčik-equation at T = 300 K, c = 1.0 mM, α = 0.5 and electrode diameter r = 1.5 mm:

$$i_p = 0.496\, \sqrt{\propto n'}\, n\, F\, A\, c\, \left(\frac{FvD}{RT}\right)^{1/2}$$

$E_{(1)}$ [i_{pc}] = 1.14; $E_{(2)}$ [i_{pc}] = 1.76; $E_{(3)}$ [i_{pc}] = 1.81

Tab. S6: Comparison of Co-N-distances for the $Co^{3+/2+}$ redox couple based on single crystal measurements of **1-Co^{III}** and DFT calculations for **1-Co^{II}.**

	Co^{3+} (from SC-X-Ray)	Co^{2+} (from DFT)
Co-$^{a}N_{eqA}$	1.9302 pm<br>1.94069 pm	1.996 pm<br>1.999 pm
Co-$^{b}N_{eqB}$	1.92308 pm<br>1.93375 pm	1.983 pm<br>1.975 pm
Co-N_{ax}	1.92410 pm	2.189 pm
Co-MeCN	1.90797 pm	2.218 pm

^{a}Pyridyl-rings connected to CH-bridge. ^{b}Pyridyl-rings connected to CH_2-bridge.

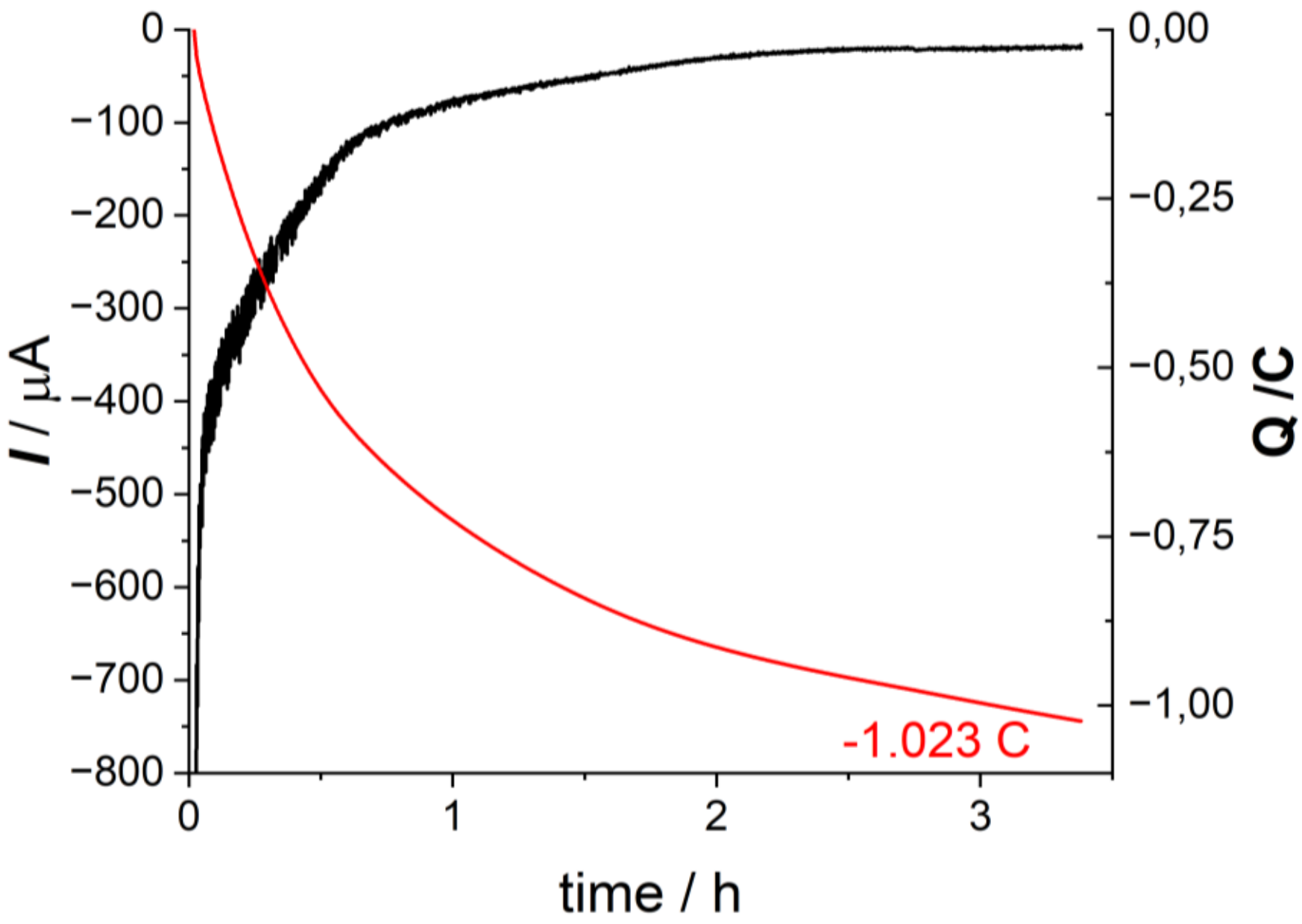

Fig. S17: CPE of 5.8 mg **1-Co^{III}** in 4 mL 50 mM TBANTf$_2$ (1.03 mM) at -1.6 V. The total charge of 1.023 C is within 86 % of the theoretical maximum of 1.189 C for a three electron reduction.

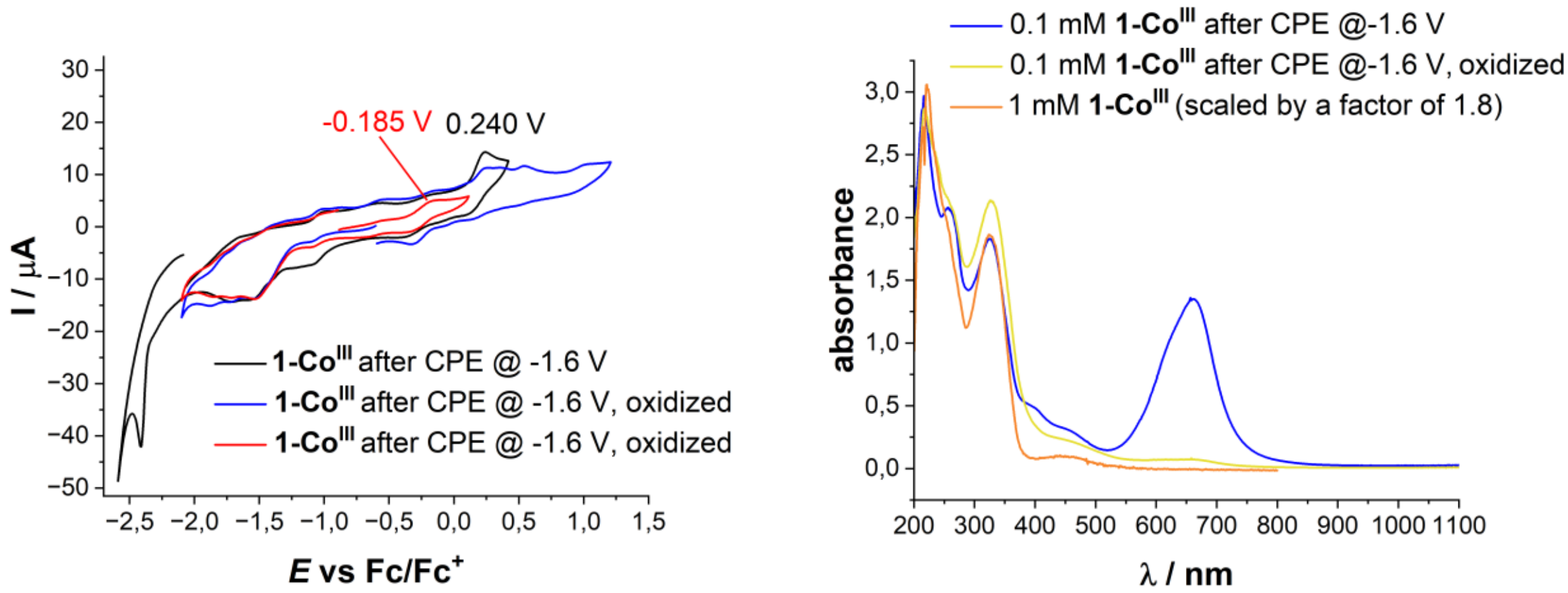

Fig. S18: Left: Measured CVs of 1 mL 1.03 mM solution of **1-Co^{III}** after CPE at -1.6 V: directly after CPE (black) and several hours later (blue, red). Right: Measured UV/Vis spectra of 0.1 mM solution of **1-Co^{III}** after CPE at -1.6 V directly after CPE (blue) and after several hours (yellow). A spectra of pure **1-Co^{III}** (orange) is shown for comparison.

Focussing on $E_{(1)}$, CVs measured at 0.02 V/s ≤ v ≤ 0.1 V/s display quasi-reversible behavior with a $\Delta E_{pc\text{-}a}$ = 0.27 V (0.1 V/s) before the current response of E_{pa} vanishes at higher scan rates (cf. Figure S16A). This is accompanied by a slight decrease in i_{pc} and a subsequent shift in E_{pc} from -0.135 V (0.02 V/s) to -0.323 V (2.0 V/s). A similar, albeit more pronounced behavior, can be seen for $E_{(2)}$ and $E_{(3)}$, with both redox-events exhibiting irreversible characteristics even at low scan rates (cf. Figure S16B). Small anodic responses can be estimated at -1.5 V and -1.1 V at v = 0.01 V/s, 0.02 V/s, but both peaks vanish at v < 0.02 V/s. We applied the Randles-Ševčik-equation (D = $4.275 \cdot 10^{-9}$ m^2/s, cf. Figure. S14) on plots of i_{pc} vs $v^{1/2}$ for diffusion controlled v to calculate the number of transferred electrons n per reduction step, giving $n[E_{(1)}]$ = 0.97 (cf. Figure S16D). This - combined with a stable OCP at -0.08 V and previous studies on the azo-free system[10] - assigns $E_{(1)}$ to the $Co^{3+/2+}$ redox-couple which undergoes one-electron reduction based on an EC_{rev}-type mechanism, as indicated by the data.[11] The rather large peak-to-peak separation is based on inner rearrangement of the Co^{n+}-L distances as evident by

comparison of crystal data obtained for **1-Co^{III}** in the Co^{3+} redox state with its calculated Co^{2+} congener (cf. tab. S5).

Due to the strictly irreversible character of both $E_{(2)}$ and $E_{(3)}$ reduction processes, leading to conflicting results for the calculated number of electrons involved (cf. tab. S4, ff). Trying to identify the new species, we conducted CV and UVvis of the deep blue (cf. Figure S17). CV revealed multiple smaller reduction peaks in the range between -1.4 and -1.6 V, indicating small residues of unreduced **1-Co^{III}**. A small irreversible oxidation at E_{pa} = 0.240 V is the only characteristic redox event in the direct aftermath of the electrolysis (cf. Figure S18-left, black). However, this system is not stable as control measurements after a few hours led to different voltammograms with a new redox system at -0.185 V (cf. Figure S18-left, red and blue). UVvis spectra of the reduced species revealed an intense absorption band at 675 nm with a small shoulder at ca. 610 nm leading to the characteristic blue color of the solution (cf. Figure S18-right, blue). The band decreases after a few hours, presumably due to decomposition, evolving into an absorption spectrum with some similarities to an authentic sample of **1-Co^{III}** (cf. Figure S18-right, yellow, orange). Although, we are unable to unambiguously identify the nature of the three-electron reduced species of **1-Co^{III}**, our data indicates two possible reduction mechanisms: Based on previous results, both reduction peaks $E_{(2)}$ and $E_{(3)}$ of **1-Co^{III}** are in the range for the reduction of Co^{2+} to Co^{1+},[5,10] together with the reduction of the azo-moiety of **1** itself. In scenario (1), initially Co^{3+} reduction is preceded by a one-electron reduction of the azo-moiety, a stark contrast compared to the two-electron reduction of free **1**. The third electron is consumed to produce a Co^{1+}-center, overall generating an $\{Co^{I}[N{=}N]^{-1}\}^{0}$ complex species. Alternatively, the reduction of the azo-moiety is split into two separate steps for **1-Co^{III}.** Thus $E_{(2)}$ and $E_{(3)}$ would correspond to the $[N{=}N]^{-}$ and $[N{=}N]^{-2}$ state of the complex, implying that the azo reduction suppresses Co^{II} to Co^{I} reduction. This scenario (2) would generate a $\{Co^{II}[N{=}N]^{-2}\}^{0}$ complex species.

UV/vis-Spectro Electrochemistry

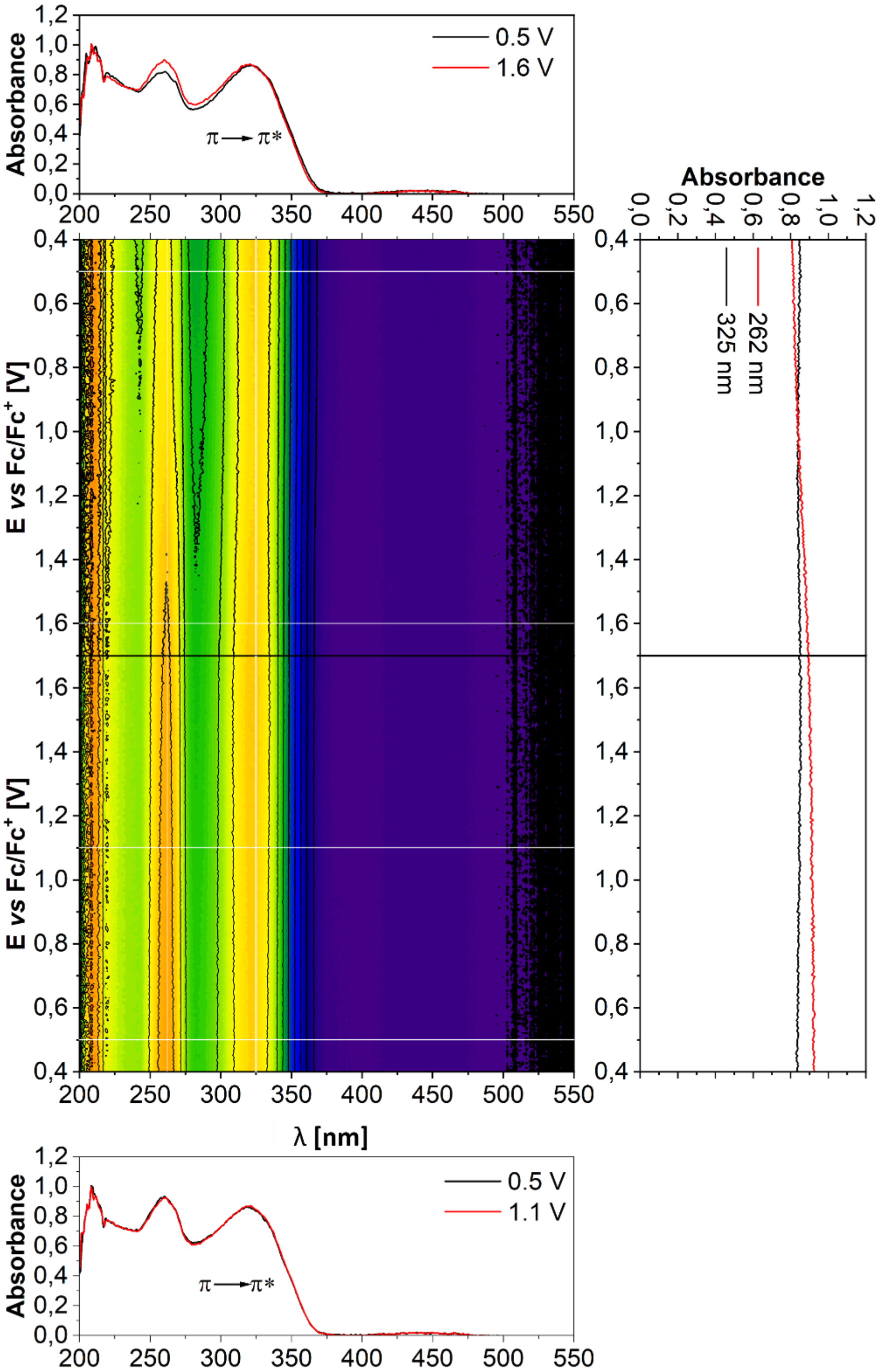

Fig. S19: UV/Vis-SEC of 0.5 mM **1** in 50 mM TBANTf$_2$ in abs. MeCN at 0.02 V/s in the oxidative range.

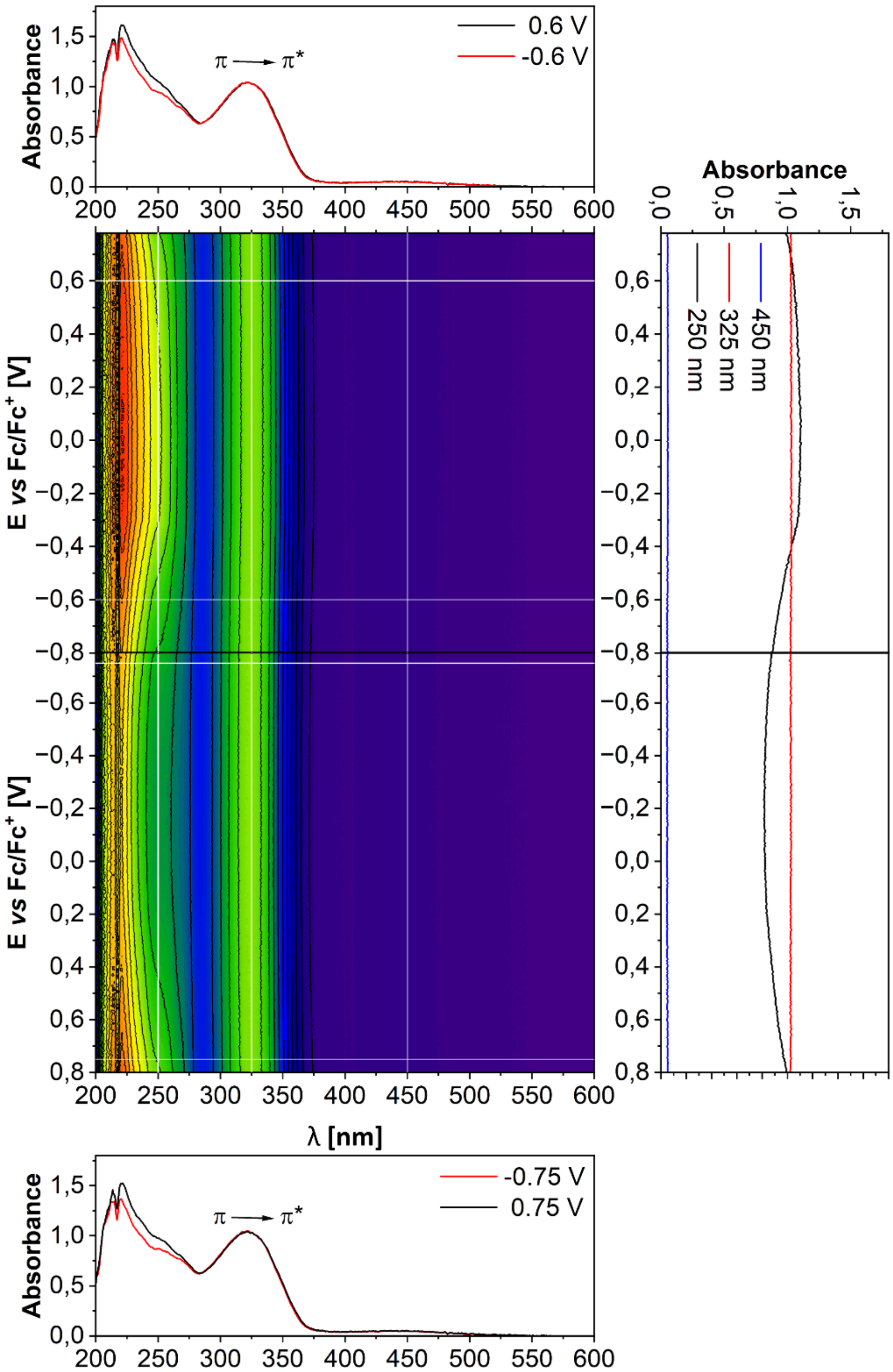

Fig. S20: UVvis-SEC of 1 mM **1-Co^{III}** in 50 mM TBA-NTf$_2$ in abs. MeCN at 0.02 V/s at the Co(III/II) redox-couple range.

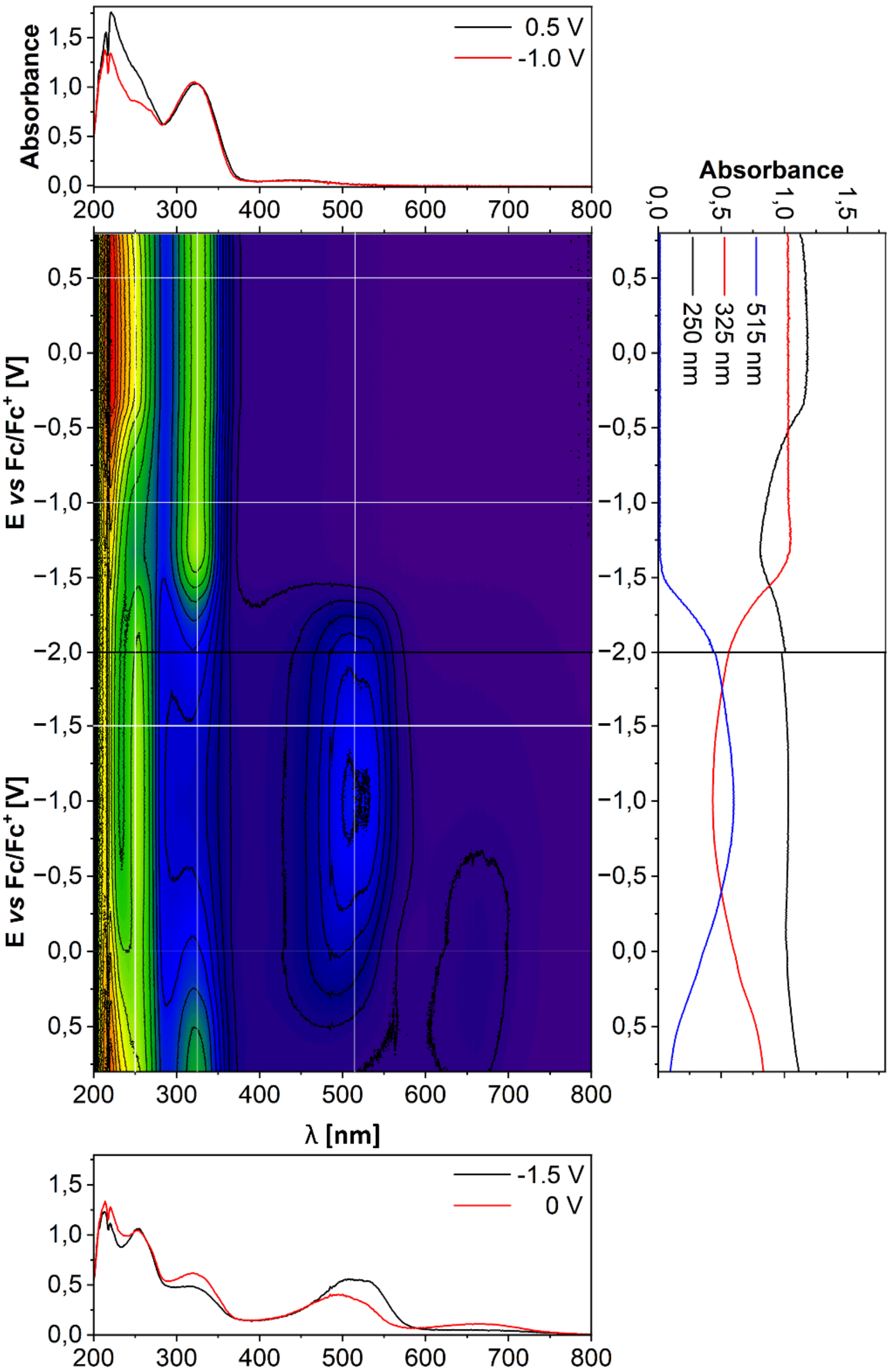

Fig. S21: UVvis-SEC of 1 mM **1-Co^{III}** in 50 mM TBANTf$_2$ in abs. MeCN at 0.02 V/s over the full potential range of the system.

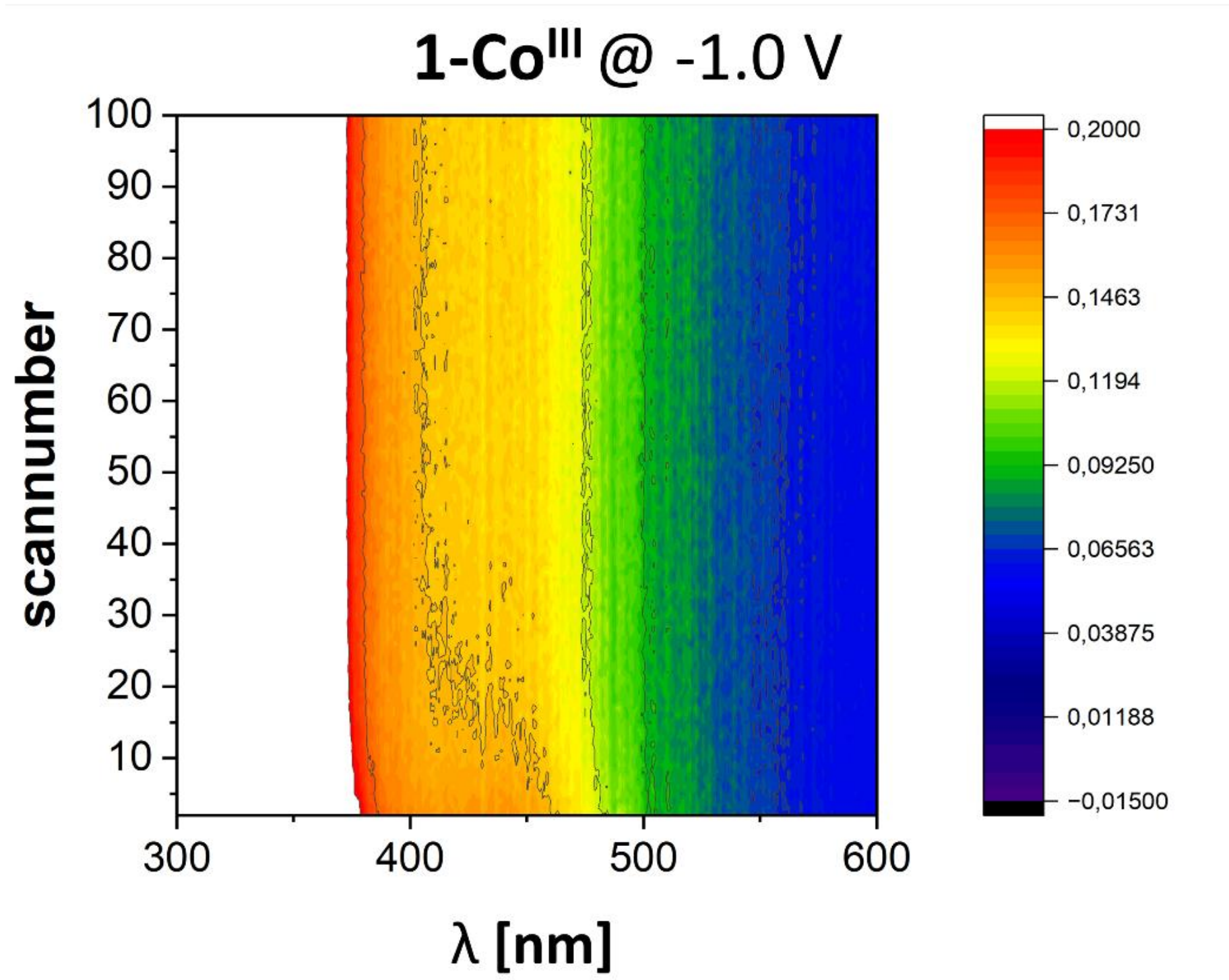

Fig. S22: UV/vis-SEC of **1-Co^III** in 50 mM TBANTf$_2$/abs. MeCN under CPE conditions at -1.0 V.

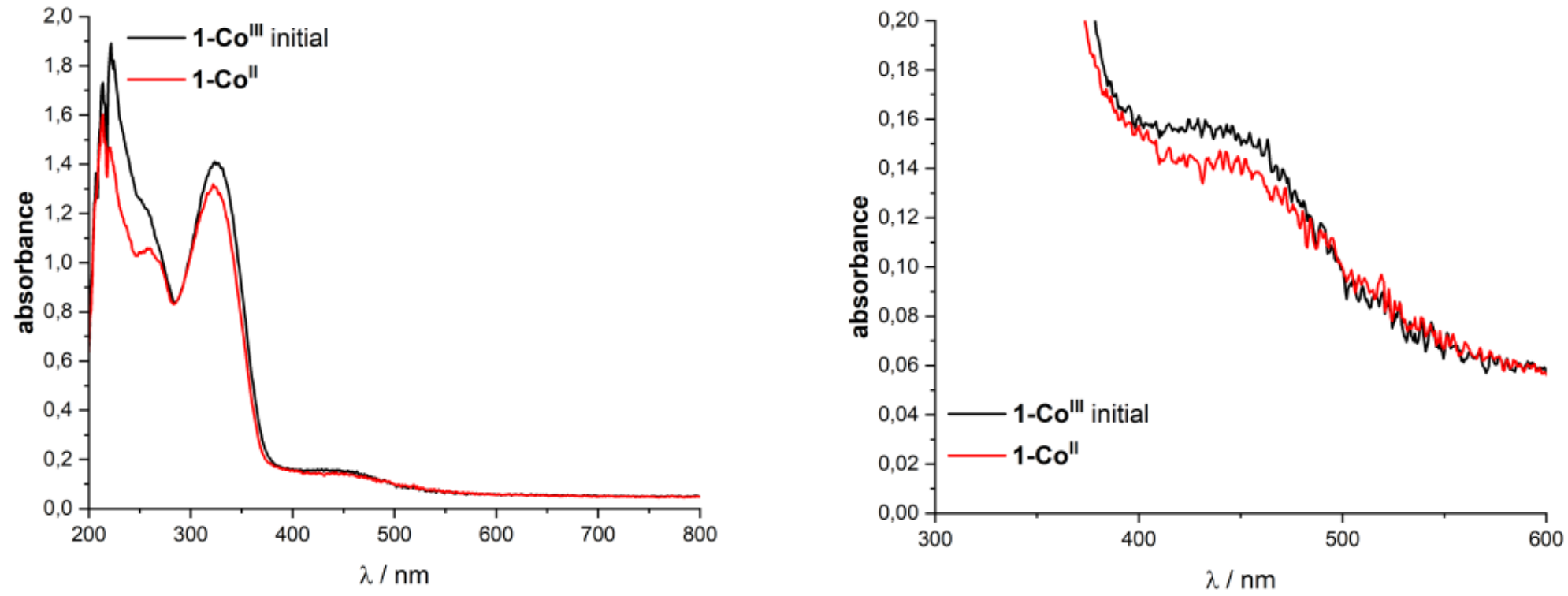

Fig. S23: UV/vis-SEC of **1-Co^III** in 50 mM TBANTf$_2$/abs. MeCN under CPE conditions at -1.0 V.

Steady-State Emission Spectra

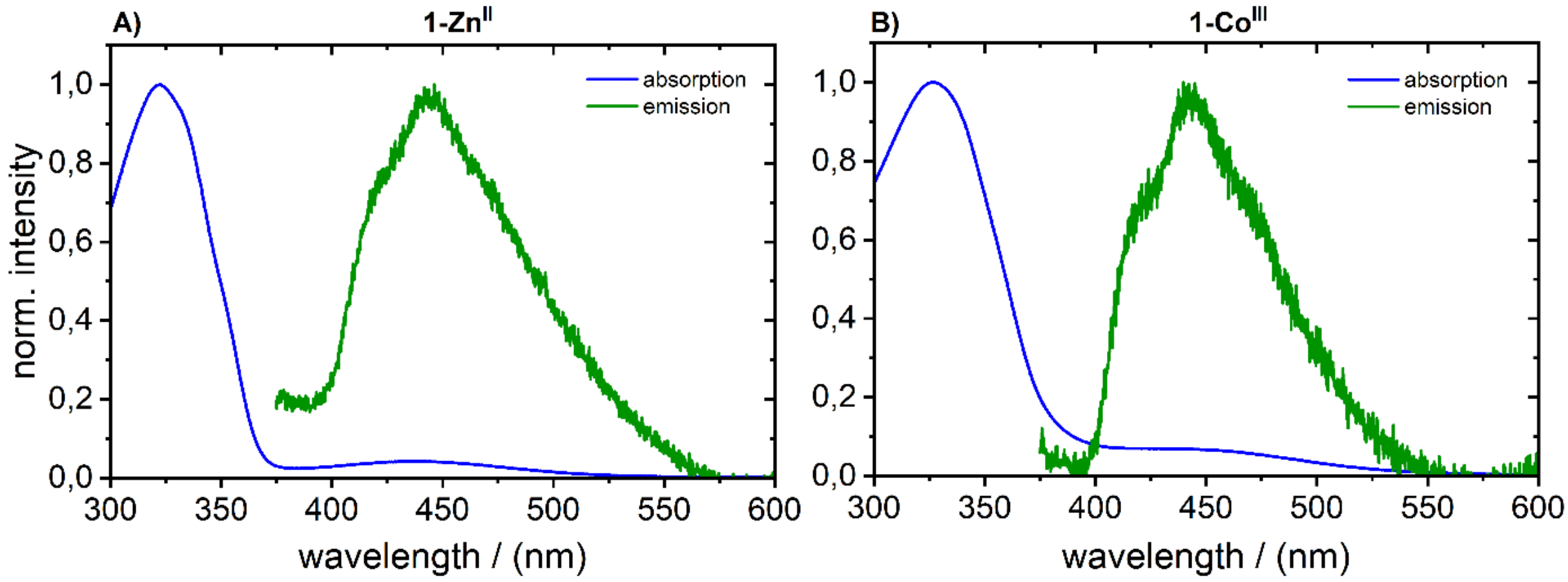

Fig. S24: Steady-state absorption (blue) and emission spectra (green, $\lambda_{exc.}$ = 315 nm) of A) **1-Zn^{II}** and B) **1-Co^{III}** in acetonitrile. Emission spectra were recorded using a Horiba JobinYvon FluoroMax-4 spectrofluorometer with slit width of 2 nm. Sample concentrations were approximately 1.5 µM, corresponding to optical densities below 0.05 at the excitation wavelength, in order to minimize inner-filter and self-absorption effects.

DFT calculations

Tab. S7: Coordinates of the DFT-calculated structures of [Co(3AzoN4Py)(MeCN)]$^{2+}$ d^7 S= 3/2 state used to compare the bond lengths between **1-Co^{III}** and **1-Co^{II}** redox-states (Tab. S4).

[Co(3AzoN4Py)(MeCN)]$^{2+}$ d^7 S= 3/2			
	x	y	z
Co	3.23405694204190	11.65976586842050	8.04464506081909
N	1.81921121804877	10.41494499128984	8.70994553044689
N	4.26763382803793	11.16822077443759	9.67962977402745
N	2.25396742478546	13.00594993442959	9.46588376774280
N	4.40945413434670	13.22895855144329	7.74520837525990
N	1.95020502460465	12.45547154962824	6.77166701937731
N	1.27268819298941	12.65290443088122	3.21161150139739
N	0.45580693209037	13.25946941524150	2.47653381635816
C	1.23887363916871	9.38190322373853	8.09613525105014
H	1.61263603352175	9.13623294580922	7.11191794262982
C	0.21002252233277	8.66315258167568	8.68703623208805
H	-0.23564772569243	7.83029702001118	8.16184934892779
C	-0.22849196478377	9.03585118561416	9.94984373521798
H	-1.03110268933665	8.49643632106902	10.43476253063545
C	0.37215400999396	10.11992605363566	10.58360372300888
H	0.04721986562240	10.44372906093245	11.56312375784079
C	1.39230993269371	10.78659380312135	9.93128004991367
C	2.16102578226047	11.96211823083754	10.51302103234099
H	1.68804993461083	12.30682628551893	11.43691191421915
C	3.56667155321682	11.45527173868349	10.79276230796007
C	4.08984638213464	11.25900306440029	12.05695706419416
H	3.50460790858499	11.50290608221376	12.93323661405278
C	5.38085871402589	10.75128199408459	12.17377821772757
H	5.81715822165396	10.58932019478918	13.15035723999639
C	6.10034460715195	10.46315743721732	11.02264525377844
H	7.10606088449365	10.07068131019684	11.07365679558193
C	5.50793839562335	10.68832879719178	9.78857270305339

H	6.02700943290249	10.48377727551275	8.86248586105152
C	3.20464822787630	14.09732038623079	9.69924306608233
H	2.68157979240081	15.03899168358505	9.88076355413734
H	3.77719149331626	13.88805565510820	10.60451393964994
C	4.18541909672390	14.27816816230235	8.55556792219570
C	4.86467803556449	15.47604873357991	8.38378718563589
H	4.65985060089564	16.30913068016422	9.04326335566338
C	5.79969585251193	15.59096838688939	7.36563864967537
H	6.33720000115787	16.51809492221849	7.21797116313635
C	6.02731828604007	14.49994368340745	6.53652843343925
H	6.74217614099957	14.54607687975005	5.72733921352955
C	5.30727139640079	13.34015963025665	6.75693730842486
H	5.43506008236828	12.46798534786740	6.13188512272545
C	1.00001197847125	13.41522831771506	8.82190997043774
H	0.18693999245771	12.78640851975354	9.18974265949249
H	0.73761845974459	14.43941360066514	9.09754108587684
C	1.04915006274380	13.29483683028338	7.31165319772013
C	1.98414393567192	12.28586449777324	5.44664299461462
H	2.72953956644184	11.61205706786166	5.04940481564171
C	1.11294841277642	12.93931550120311	4.58220473004153
C	0.17619318837089	13.81888896873078	5.13607544336749
H	-0.50536233037980	14.34735781851641	4.48485440661256
C	0.15476943737248	13.99266916240370	6.50681074524119
H	-0.55549133343975	14.67039063042845	6.96296918102941
C	0.54757813716182	13.03177325774138	1.10224867517313
C	1.49093154076128	12.18679236784236	0.49467240442071
H	2.20912813701037	11.66376260974856	1.10890894222524
C	1.48296090955215	12.04214474735402	-0.87779204791428
H	2.20468216188707	11.39498837501823	-1.35850021875097
C	0.54473259151195	12.72955218066210	-1.65429736306249
H	0.54794078442097	12.60824241138694	-2.72966586746685
C	-0.38984966564879	13.56678964377205	-1.05538503698701
H	-1.11291316478606	14.09624740911471	-1.66068076921696
C	-0.38958304066890	13.71963769349880	0.32198106420641
H	-1.10299995998269	14.36401368953386	0.81782613396134
N	4.19969232220312	10.23144631380680	6.71422139856629
C	4.66117452977053	9.38841638664625	6.08434459971180
C	5.24107480781313	8.32456598446125	5.28656223979084
H	4.68936933701063	8.21996151561234	4.35132023917048
H	5.19590162352842	7.38243495949896	5.83456200264790
H	6.28304943484251	8.55463526557871	5.06016303845392

Tab. S8: Coordinates of the DFT-calculated structures of **1-Co^{III}** in its singlet ground state ($^1S_0$) and the in its triplet excited state ($^3T_1$) used to model the difference spectrum of the ^{3}MC state.

[Co(3AzoN4Py)(MeCN)]$^{3+}$ S=0				[Co(3AzoN4Py)(MeCN)]$^{3+}$ S=1			
N	-0.22604	0.11342	1.81808	N	-0.06448	0.12713	2.01853
C	-1.54929	-4.12330	0.96868	C	-1.35985	-4.22510	0.65338
C	-1.04615	-2.83137	0.96630	C	-0.91554	-2.91704	0.76223
N	-1.69426	-1.84278	0.34564	N	-1.61620	-1.90928	0.23435
C	-2.84760	-2.09403	-0.30524	C	-2.76281	-2.14548	-0.42743
C	-3.40315	-3.35583	-0.34865	C	-3.25754	-3.42457	-0.58270
C	-2.73843	-4.38849	0.30616	C	-2.54146	-4.48110	-0.02741
C	-4.49618	1.39219	2.54362	C	-4.52945	1.06155	2.70505
C	-5.54420	0.87303	1.79841	C	-5.55789	0.54358	1.93326
C	-5.26995	0.14940	0.64153	C	-5.26075	-0.08485	0.72734
C	-3.94986	-0.02750	0.28264	C	-3.93985	-0.17251	0.33720
N	-2.93652	0.47925	1.01271	N	-2.94813	0.33407	1.09901
C	-3.19329	1.18175	2.11873	C	-3.22353	0.94203	2.25611
C	-0.04462	-0.73352	-2.24955	C	-0.07228	-0.54722	-2.25284
N	0.31578	-0.40677	-0.99007	N	0.31492	-0.28653	-0.98329
C	1.60359	-0.36460	-0.64600	C	1.60654	-0.25753	-0.65297
C	2.61029	-0.66066	-1.56208	C	2.60170	-0.49775	-1.59693
C	2.24723	-1.01096	-2.85972	C	2.22015	-0.77112	-2.90795
C	0.90755	-1.03686	-3.20765	C	0.87658	-0.78589	-3.23491
N	-0.94418	1.94657	-0.31888	N	-1.06912	2.02894	-0.29277
C	-0.08782	2.80688	0.25230	C	-0.23017	2.91318	0.26981
C	0.05030	4.10327	-0.20508	C	-0.17022	4.22437	-0.15904
C	-0.70958	4.51823	-1.29051	C	-0.98979	4.62288	-1.20796
C	-1.58713	3.62332	-1.88296	C	-1.84679	3.70040	-1.78756
C	-1.68695	2.34043	-1.37065	C	-1.87156	2.40156	-1.30262
C	5.63159	-0.10092	0.20635	C	5.65289	-0.09043	0.16257
C	6.66015	-0.68448	-0.54755	C	6.66755	-0.57756	-0.67450

C	7.96266	-0.61267	-0.08759	C	7.97997	-0.54281	-0.23982
C	8.25265	0.03521	1.11589	C	8.29359	-0.02692	1.02030
C	7.23311	0.61332	1.86427	C	7.28798	0.45607	1.85062
C	5.92284	0.54477	1.41142	C	5.96773	0.42390	1.42364
N	4.27056	-0.10899	-0.15709	N	4.28431	-0.07679	-0.16852
N	3.98896	-0.64962	-1.24750	N	3.98394	-0.50162	-1.30346
C	-1.51834	-0.80451	-2.51643	C	-1.54792	-0.63019	-2.56197
N	-2.27869	-0.05316	-1.45804	N	-2.38594	-0.00536	-1.50736
C	-2.65462	1.33094	-1.91172	C	-2.82769	1.36804	-1.84429
C	-3.44619	-0.83632	-0.88949	C	-3.44290	-0.87853	-0.90980
C	0.28902	0.10157	2.84112	C	0.47057	0.09629	3.03323
C	0.94135	0.09033	4.12810	C	1.14705	0.06186	4.30677
H	1.85060	-0.10141	0.36958	H	1.86535	-0.04666	0.37247
H	6.42584	-1.18354	-1.47666	H	6.41451	-0.97521	-1.64668
H	8.76185	-1.06122	-0.66325	H	8.76850	-0.91785	-0.87922
H	9.27554	0.08574	1.46614	H	9.32414	-0.00464	1.35041
H	7.45819	1.11465	2.79621	H	7.53187	0.85511	2.82617
H	5.11160	0.98630	1.97505	H	5.16688	0.79324	2.05043
H	-1.76559	-0.40920	-3.49906	H	-1.74686	-0.16203	-3.52500
H	-1.83716	-1.84646	-2.50717	H	-1.82227	-1.67991	-2.67161
H	-3.65578	1.56290	-1.55005	H	-3.81285	1.54826	-1.41346
H	-2.69639	1.36343	-2.99779	H	-2.93794	1.47889	-2.92224
H	-4.21147	-1.03079	-1.63740	H	-4.25534	-1.09740	-1.60125
H	1.91405	-0.39304	4.03667	H	1.99877	-0.61589	4.24700
H	0.32493	-0.45972	4.83951	H	0.45609	-0.28459	5.07533
H	1.07500	1.11543	4.47449	H	1.49638	1.06425	4.55593
H	-2.19177	3.90934	-2.73189	H	-2.49461	3.97643	-2.60774
H	-6.06111	-0.26432	0.03294	H	-6.03884	-0.49361	0.09872
H	-0.12022	-2.58423	1.46262	H	0.00153	-2.66958	1.27696
H	-1.00648	-4.90126	1.48500	H	-0.78018	-5.02146	1.09631
H	0.49179	2.44479	1.08640	H	0.39221	2.55565	1.07624
H	-0.61689	5.52463	-1.67466	H	-0.95764	5.64001	-1.57369
H	0.74907	4.76606	0.28364	H	0.51372	4.91019	0.31877
H	-3.14624	-5.38969	0.29200	H	-2.90411	-5.49439	-0.13151
H	-4.32719	-3.52736	-0.88174	H	-4.17560	-3.58989	-1.12802
H	-2.34996	1.57268	2.66705	H	-2.39048	1.32950	2.82258
H	-6.56785	1.02928	2.10932	H	-6.58480	0.62724	2.26103
H	-4.67310	1.95758	3.44669	H	-4.72165	1.55388	3.64688
H	0.59776	-1.29488	-4.21036	H	0.55391	-0.98917	-4.24632
H	3.01696	-1.24945	-3.58104	H	2.97966	-0.96214	-3.65386
Co	-1.20478	0.06247	0.15429	Co	-1.18079	0.07938	0.25278

References

(1) Megow, S.; Fitschen, H.-L.; Tuczek, F.; Temps, F. Ultrafast Photodynamics of an Azopyridine-Functionalized Iron(II) Complex: Implications for the Concept of Ligand-Driven Light-Induced Spin Change. *J. Phys. Chem.* **2019**, *10*, 6048–6054. DOI: 10.1021/acs.jpclett.9b02083.

(2) Matsumiya, M.; Kamo, Y.; Hata, K.; Tsunashima, K. Solvation structure of iron group metal ion in TFSA-based ionic liquids investigated by Raman spectroscopy and DFT calculations. *Journal of Molecular Structure* **2013**, *1048*, 59–63. DOI: 10.1016/j.molstruc.2013.04.023.
(3) Williams, D. B.; Stoll, M. E.; Scott, B. L.; Costa, D. A.; Oldham, W. J. Coordination chemistry of the bis(trifluoromethylsulfonyl)imide anion: molecular interactions in room temperature ionic liquids. *Chemical communications (Cambridge, England)* **2005** (11), 1438–1440. DOI: 10.1039/b416830e. Published Online: Jan. 21, 2005.
(4) Sahu, S.; Widger, L. R.; Quesne, M. G.; Visser, S. P. de; Matsumura, H.; Moënne-Loccoz, P.; Siegler, M. A.; Goldberg, D. P. Secondary coordination sphere influence on the reactivity of nonheme iron(II) complexes: an experimental and DFT approach. *J. Am. Chem. Soc.* **2013**, *135* (29), 10590–10593. DOI: 10.1021/ja402688t. Published Online: Jul. 15, 2013.
(5) Yamaguchi, K.; Kume, S.; Namiki, K.; Murata, M.; Tamai, N.; Nishihara, H. UV-Vis, NMR, and Time-Resolved Spectroscopy Analysis of Photoisomerization Behavior of Three- and Six-Azobenzene-Bound Tris(bipyridine)cobalt Complexes. *Inorganic chemistry* **2005**, *44* (24), 9056–9067. DOI: 10.1021/ic0513538.
(6) Savéant, J. M.; Costentin, C. *Elements of molecular and biomolecular electrochemistry*:*An electrochemical approach to electron transfer chemistry*, Second edition; Wiley, 2019.
(7) Klopman, G.; Doddapaneni, N. Electrochemical behavior of cis and trans azobenzenes. *J. Phys. Chem.* **1974**, *78* (18), 1825–1828. DOI: 10.1021/j100611a011.
(8) Otsuki, J.; Omokawa, N.; Yoshiba, K.; Yoshikawa, I.; Akasaka, T.; Suenobu, T.; Takido, T.; Araki, K.; Fukuzumi, S. Synthesis and structural, electrochemical, and optical properties of Ru(II) complexes with azobis(2,2'-bipyridine)s. *Inorg. Chem.* **2003**, *42* (9), 3057–3066. DOI: 10.1021/ic026040g.
(9) Xie, Y.; Li, M.; Ma, Y.; Lin, F.; Zhu, H.; Li, W.; Jiang, S.; Shen, C.; Jia, Z.; Zhang, K. Azopyridine Aqueous Electrochemistry Enables Superior Organic AZIBs. *ACS applied materials & interfaces* **2024**, *16* (44), 60132–60141. DOI: 10.1021/acsami.4c09801. Published Online: Oct. 23, 2024.
(10) Lo, W. K. C.; Castillo, C. E.; Gueret, R.; Fortage, J.; Rebarz, M.; Sliwa, M.; Thomas, F.; McAdam, C. J.; Jameson, G. B.; McMorran, D. A.; Crowley, J. D.; Collomb, M.-N.; Blackman, A. G. Synthesis, Characterization, and Photocatalytic H2-Evolving Activity of a Family of Co(N4Py)(X)(n+) Complexes in Aqueous Solution. *Inorganic chemistry* **2016**, *55* (9), 4564–4581. DOI: 10.1021/acs.inorgchem.6b00391. Published Online: Apr. 11, 2016.
(11) Schnaubelt, L.; Petzold, H.; Speck, J. M.; Dmitrieva, E.; Rosenkranz, M.; Korb, M. Redox properties and electron transfer in a triarylamine-substituted HS-Co2+/LS-Co3+ redox couple. *Dalton transactions (Cambridge, England : 2003)* **2017**, *46* (8), 2690–2698. DOI: 10.1039/C6DT04748C.
(12) Bykov, D.; Petrenko, T.; Izsák, R.; Kossmann, S.; Becker, U.; Valeev, E.; Neese, F. Efficient implementation of the analytic second derivatives of Hartree–Fock and hybrid DFT energies: a detailed analysis of different approximations. *Molecular Physics* **2015**, *113* (13-14), 1961–1977. DOI: 10.1080/00268976.2015.1025114.
(13) Caldeweyher, E.; Bannwarth, C.; Grimme, S. Extension of the D3 dispersion coefficient model. *The Journal of Chemical Physics* **2017**, *147* (3), 34112. DOI: 10.1063/1.4993215.
(14) Caldeweyher, E.; Ehlert, S.; Hansen, A.; Neugebauer, H.; Spicher, S.; Bannwarth, C.; Grimme, S. A generally applicable atomic-charge dependent London dispersion correction. *The Journal of Chemical Physics* **2019**, *150* (15), 154122. DOI: 10.1063/1.5090222.
(15) Caldeweyher, E.; Mewes, J.-M.; Ehlert, S.; Grimme, S. Extension and evaluation of the D4 London-dispersion model for periodic systems. *Physical chemistry chemical physics : PCCP* **2020**, *22* (16), 8499–8512. DOI: 10.1039/D0CP00502A.

(16) Garcia-Ratés, M.; Neese, F. Effect of the Solute Cavity on the Solvation Energy and its Derivatives within the Framework of the Gaussian Charge Scheme. *Journal of computational chemistry* **2020**, *41* (9), 922–939. DOI: 10.1002/jcc.26139. Published Online: Dec. 30, 2019.
(17) Grimme, S.; Antony, J.; Ehrlich, S.; Krieg, H. A consistent and accurate ab initio parametrization of density functional dispersion correction (DFT-D) for the 94 elements H-Pu. *The Journal of Chemical Physics* **2010**, *132* (15), 154104. DOI: 10.1063/1.3382344.
(18) Grimme, S.; Ehrlich, S.; Goerigk, L. Effect of the damping function in dispersion corrected density functional theory. *Journal of computational chemistry* **2011**, *32* (7), 1456–1465. DOI: 10.1002/jcc.21759. Published Online: Mar. 1, 2011.
(19) Helmich-Paris, B.; Souza, B. de; Neese, F.; Izsák, R. An improved chain of spheres for exchange algorithm. *The Journal of Chemical Physics* **2021**, *155* (10), 104109. DOI: 10.1063/5.0058766.
(20) Neese, F. An improvement of the resolution of the identity approximation for the formation of the Coulomb matrix. *Journal of computational chemistry* **2003**, *24* (14), 1740–1747. DOI: 10.1002/jcc.10318.
(21) Neese, F. Software update: The ORCA program system—Version 5.0. *WIREs Comput Mol Sci* **2022**, *12* (5). DOI: 10.1002/wcms.1606.
(22) Neese, F. The SHARK integral generation and digestion system. *Journal of computational chemistry* **2023**, *44* (3), 381–396. DOI: 10.1002/jcc.26942. Published Online: Jun. 9, 2022.
(23) Neese, F.; Olbrich, G. Efficient use of the resolution of the identity approximation in time-dependent density functional calculations with hybrid density functionals. *Chemical Physics Letters* **2002**, *362* (1-2), 170–178. DOI: 10.1016/S0009-2614(02)01053-9.
(24) Neese, F.; Wennmohs, F.; Hansen, A.; Becker, U. Efficient, approximate and parallel Hartree–Fock and hybrid DFT calculations. A 'chain-of-spheres' algorithm for the Hartree–Fock exchange. *Chemical Physics* **2009**, *356* (1-3), 98–109. DOI: 10.1016/j.chemphys.2008.10.036.
(25) Wittmann, L.; Gordiy, I.; Friede, M.; Helmich-Paris, B.; Grimme, S.; Hansen, A.; Bursch, M. Extension of the D3 and D4 London dispersion corrections to the full actinides series. *Physical chemistry chemical physics : PCCP* **2024**, *26* (32), 21379–21394. DOI: 10.1039/D4CP01514B. Published Online: Aug. 14, 2024.
(26) Sinha, N.; Wellauer, J.; Maisuradze, T.; Prescimone, A.; Kupfer, S.; Wenger, O. S. Reversible Photoinduced Ligand Substitution in a Luminescent Chromium(0) Complex. *J. Am. Chem. Soc.* **2024**, *146* (15), 10418–10431. DOI: 10.1021/jacs.3c13925. Published Online: Apr. 8, 2024.
(27) Wegeberg, C.; Häussinger, D.; Kupfer, S.; Wenger, O. S. Controlling the Photophysical Properties of a Series of Isostructural d6 Complexes Based on Cr0, MnI, and FeII. *J. Am. Chem. Soc.* **2024**, *146* (7), 4605–4619. DOI: 10.1021/jacs.3c11580. Published Online: Feb. 9, 2024.
(28) Witas, K.; Nair, S. S.; Maisuradze, T.; Zedler, L.; Schmidt, H.; Garcia-Porta, P.; Rein, A. S. J.; Bolter, T.; Rau, S.; Kupfer, S.; Dietzek-Ivanšić, B.; Sorsche, D. U. Beyond the First Coordination Sphere─Manipulating the Excited-State Landscape in Iron(II) Chromophores with Protons. *J. Am. Chem. Soc.* **2024**, *146* (29), 19710–19719. DOI: 10.1021/jacs.4c00552. Published Online: Jul. 11, 2024.
